# Selective Estrogen Receptor β Agonist LY500307 as a Novel Therapeutic Agent for Glioblastoma

**DOI:** 10.1038/srep24185

**Published:** 2016-04-29

**Authors:** Gangadhara R. Sareddy, Xiaonan Li, Jinyou Liu, Suryavathi Viswanadhapalli, Lauren Garcia, Aleksandra Gruslova, David Cavazos, Mike Garcia, Anders M. Strom, Jan-Ake Gustafsson, Rajeshwar Rao Tekmal, Andrew Brenner, Ratna K. Vadlamudi

**Affiliations:** 1 The Department of Obstetrics and Gynecology, University of Texas Health Science Center at San Antonio, San Antonio TX 78229, USA; 2 Cancer Therapy & Research Center, University of Texas Health Science Center at San Antonio, San Antonio TX 78229, USA; 3 Hematology & Oncology, University of Texas Health Science Center at San Antonio, San Antonio TX 78229, USA; 4 University of Houston, Houston, TX 77004, USA.

## Abstract

Glioblastomas (GBM), deadly brain tumors, have greater incidence in males than females. Epidemiological evidence supports a tumor suppressive role of estrogen; however, estrogen as a potential therapy for GBM is limited due to safety concerns. Since GBM express ERβ, a second receptor for estrogen, targeting ERβ with a selective agonist may be a potential novel GBM therapy. In the present study, we examined the therapeutic effect of the selective synthetic ERβ agonist LY500307 using *in vitro* and *in vivo* GBM models. Treatment with LY500307 significantly reduced the proliferation of GBM cells with no activity on normal astrocytes *in vitro*. ERβ agonists promoted apoptosis of GBM cells, and mechanistic studies using RNA sequencing revealed that LY500307 modulated several pathways related to apoptosis, cell cycle, and DNA damage response. Further, LY500307 sensitized GBM cells to several FDA-approved chemotherapeutic drugs including cisplatin, lomustine and temozolomide. LY500307 treatment significantly reduced the *in vivo* tumor growth and promoted apoptosis of GBM tumors in an orthotopic model and improved the overall survival of tumor-bearing mice in the GL26 syngeneic glioma model. Our results demonstrate that LY500307 has potential as a therapeutic agent for GBM.

Glioblastomas (GBM) are the most aggressive tumors accounting for 45.6% of primary malignant brain tumors^1^. The standard therapy for GBM comprises surgery followed by radiation therapy with adjuvant chemotherapy^2^,^3^,^4^. Despite advances in multimodal therapies, the median survival of patients with GBM is approximately 15 months with a 5-year survival rate of 5.0% after diagnosis^5^. The highly infiltrative, heterogeneous and mutable nature of GBM^6^ often contributes to tumor recurrence and resistance to therapies. Current cytotoxic chemotherapeutic agents used to treat GBM include carmustine, lomustine, and carboplatin^2^. A recent trial of combining bevacizumab with standard chemotherapy and radiation therapy only marginally improved overall survival^7^. Therapy regimens are currently being developed to target EGFR, VEGFR, PDGFR, Ras pathway, mTOR, histone acetylation and integrins^8^, and thus far these molecular-targeted therapies have produced poor-to-modest clinical responses^6^. Identification of more effective therapeutic agents that work as a single agent or in combination with existing drugs are clearly needed.

The incidence rate of GBM and other glial tumors is higher in males than females^1^. Females of reproductive ages demonstrate a survival advantage over both males and post-menopausal women. Usage of exogenous hormones reduces the risk of glioma development^9^,^10^,^11^,^12^. Estrogen improves the survival in a glioblastoma orthotopic animal model^13^. Estrogen mediates its effects through the estrogen receptor α (ERα) and estrogen receptor β (ERβ). ERβ has quite different function than ERα, and ERβ is considered as a tumor suppressor. Recent studies demonstrated that ERβ reduces proliferation and induces apoptosis in several cancer cells^14^,^15^,^16^,^17^,^18^,^19^,^20^ and that its expression declines during tumor progression^21^,^22^,^23^,^24^. Recent studies including ours demonstrated that GBM cells express ERβ, and high expression of ERβ was an independent favorable prognostic factor^25^,^26^,^27^. Collectively, these correlative findings suggest that estrogen and ERβ play a significant role in suppression of GBM; however, the mechanisms are poorly understood. Furthermore, estrogen as potential therapy for GBM has limited therapeutic application due to the risk of breast and uterine cancers in women, prostate cancer and feminization in men, and may increase risk of heart disease in both sexes^28^,^29^,^30^,^31^.

Even though ERα and ERβ are structurally similar, their ligand-binding domains differ enough to be selective for different ligands^32^. Recent studies identified a number of selective synthetic and natural ERβ agonists which are currently being investigated for therapeutic use^33^. One synthetic compound LY500307 is a highly potent and selective ERβ agonist; it has a 12-fold higher affinity for ERβ than ERα and exhibits 32-fold more functional potency. Further, preclinical studies showed that LY500307 treatment dose dependently reduced the prostate weight in a mouse model of benign prostatic hyperplasia^34^. LY500307 was well tolerated in BPH patients with no side effects^35^ and more importantly, LY500307 is currently being tested in phase 2 clinical trials for improving negative symptoms and cognitive impairment associated with Schizophrenia. However, it remains unknown whether LY500307 has efficacy in treating GBM.

Here, we tested the efficacy of the ERβ agonist LY500307 as a novel therapeutic agent for treating GBM using *in vitro* and *in vivo* preclinical models. Our results demonstrate that LY500307 selectively kills established and patient-derived primary GBM cells with minimal toxicity on normal cells. Mechanistic studies showed that LY500307 modulates cell cycle, apoptosis and DNA damage response pathways and sensitizes GBM cells to current chemotherapeutic agents. Further, LY500307 reduced GBM growth *in vivo* in orthotopic models and prolonged the survival of tumor-bearing mice. This represents the first report demonstrating specificity of ERβ ligand LY500307 on GBM cells and suggests that LY500307– ERβ-mediated inhibition may be an effective strategy for targeted therapy.

## Results

### Selective ERβ agonist LY500307 reduces the cell viability and survival, and induces apoptosis of GBM cells

**Figure 1:**
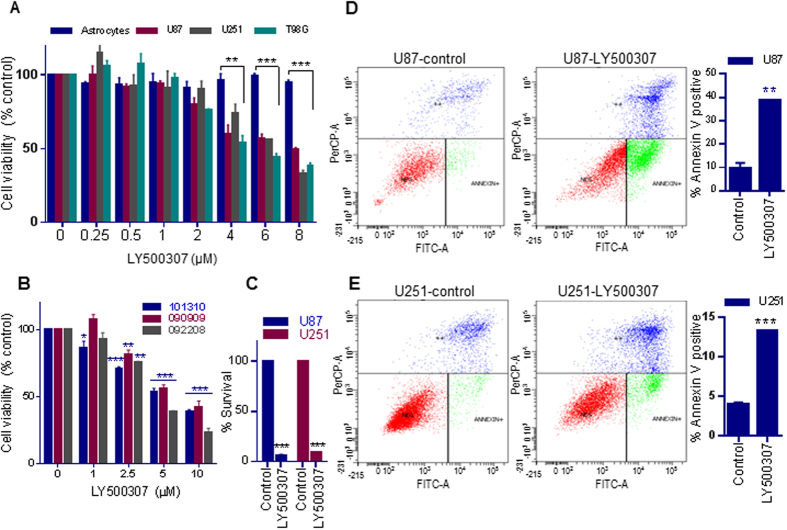
ERβ agonist L500307 reduces proliferation and induces apoptosis in GBM cells. (**A**) U87, U251, T98G and normal astrocytes were treated with either vehicle or LY500307 for 72 h and the cell viability was measured using an MTT assay. (**B**) Patient-derived primary GBM cells were treated with either vehicle or LY500307 for 72 h and the cell viability was measured using the CellTiter-Glo luminescent assay. (**C**) U87 and U251 cells were treated with either vehicle or LY500307 for 72 h and then cultured for 7 subsequent days. The number of colonies for each group was counted. (**D–E**) U87 and U251 cells were treated with either vehicle or LY500307 for 48 h followed by Annexin V-FITC and Propidium Iodide (PI) staining for 15 min. The Annexin V–positive apoptotic populations were determined using flow cytometry. Data are represented as mean ± SE. *p < 0.05, **p < 0.01, ***p < 0.001.

To test whether LY500307 reduces cell viability of GBM cells, MTT cell viability assays were performed. Treatment with LY500307 significantly reduced the viability of various GBM cell lines in a dose-dependent manner. In contrast, viability of normal astrocytes was not affected at the tested doses, suggesting that LY500307 has tumor cell–specific activity (Fig. 1a). Further the effect of LY500307 on cell viability of several patient derived GBM cells was examined. As shown in Supplementary Fig. 1a, all patient derived primary GBM cells tested expressed ERβ but not ERα. Cell Titer-Glo luminescent cell viability assays revealed that LY500307 reduced the viability of various patient-derived GBM cells in a dose dependent manner (Fig. 1b). We next examined the effect of LY500307 on survival of GBM cells using colony formation assays. As shown in Fig. 1c, LY500307 significantly reduced the colony formation of U87 and U251 GBM cells. We then tested whether LY500307 induces apoptosis of GBM cells using the Annexin V assay. LY500307 significantly induced Annexin V-positive apoptotic cells in U87, U251 (Fig. 1d,e) and patient derived primary GBM (GBM10) cells (Supplementary Fig. 1b). Collectively, these results suggested that LY500307 has potential to selectively reduce cell viability, decrease survival and induce apoptosis of GBM cells.

### LY500307 enhances ERβ signaling in GBM cells

**Figure 2:**
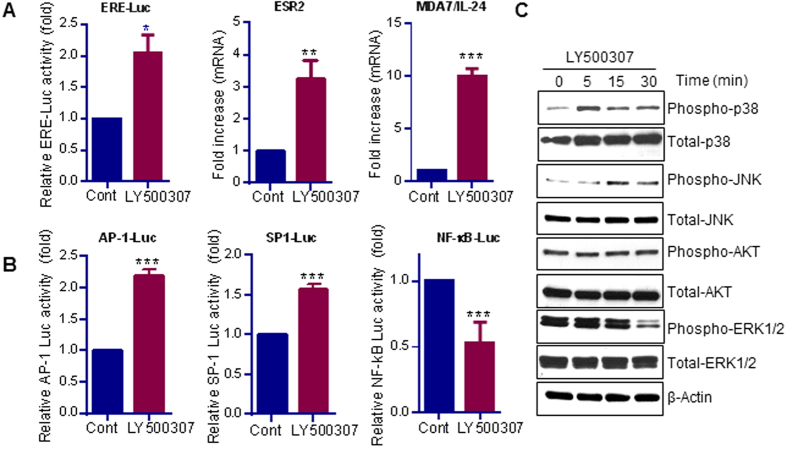
ERβ agonist LY500307 activates ERβ signaling in GBM cells. (**A**) U87 GBM cells were transfected with ERE-Luc plasmid. After 24 h, the cells were treated with either vehicle or LY500307 for additional 24 h, and then reporter activity was measured (left panel). U87 cells were treated with vehicle or LY500307 for 24 h, and the mRNA expression of ERβ (ESR2) and MDA7/IL-24 was measured using qRT-PCR (middle and right panels). (**B**) U87 cells were transfected with AP-1 luc, SP-1 luc and NF-kB luc plasmids and 24 h after transfection, the cells were treated with either vehicle or LY500307 for 24 h and reporter activity was then measured. (**C**) U87 cells were treated with either vehicle or LY500307 for 5 min, 15 min and 30 min and lysed in RIPA buffer. The lysates were subjected to Western blotting with the indicated antibodies, and β-actin used as loading control. Data are represented as mean ± SE. *p < 0.05, **p < 0.01, ***p < 0.001.

To determine whether LY500307 promotes activation of classical ERβ–ERE signaling, U87 GBM cells were transfected with the ERE-luciferase reporter and treated with LY500307 for 24 h. LY500307 significantly increased the ERE luciferase activity in GBM cells (Fig. 2a, left panel). In addition, LY500307 stimulation enhanced the expression of ERβ and its target gene MDA7/IL-24 in GBM cells (Fig. 2a, middle and right panel). To determine whether LY500307 modulates the non-classical ERβ signaling, GBM cells were transfected with AP-1, SP-1 and NF-κB-Luc luciferase reporter plasmids followed by treatment with vehicle or LY500307. As shown in Fig. 2b, LY500307 significantly increased AP-1 and SP-1 luciferase activities and reduced NF-κB-Luc activity in GBM cells. To determine whether LY500307 has an effect on ERβ-mediated rapid extra-nuclear signaling, GBM cells were treated with LY500307 for short periods of time (5, 15, and 30 min) and the phosphorylation status of p38MAPK, JNK, ERK1/2 and Akt was examined. Western blot analysis showed that LY500307 significantly increased the phosphorylation of proapoptotic stress activated kinases p38MAPK and JNK in GBM cells. However, LY500307 treatment reduced phosphorylation of ERK1/2, and Akt phosphorylation was not affected (Fig. 2c). To examine the ERβ selectivity of LY500307, cell viability assays were performed using ERβ knockdown in GBM cells. LY500307-mediated reduction in cell viability was significantly compromised in ERβ shRNA cells than in control shRNA cells, suggesting the specific requirement of ERβ for LY500307 actions (Supplementary Fig. 2). We also confirmed the ERβ-mediated GBM suppression using GBM cells that overexpress ERβ. The effect of ERβ expression on GBM cell proliferation was studied using Cell Titer-Glo assay. ERβ overexpression significantly reduced the proliferation of U87 and U251 GBM cells (Supplementary Fig. 3). Collectively, these results suggest that LY500307 has potential to modulate both classical and non-classical ERβ signaling and that LY500307 can enhance ERβ expression in GBM cells.

### Analysis of LY500307 induced global transcriptional changes in GBM cells

**Figure 3:**
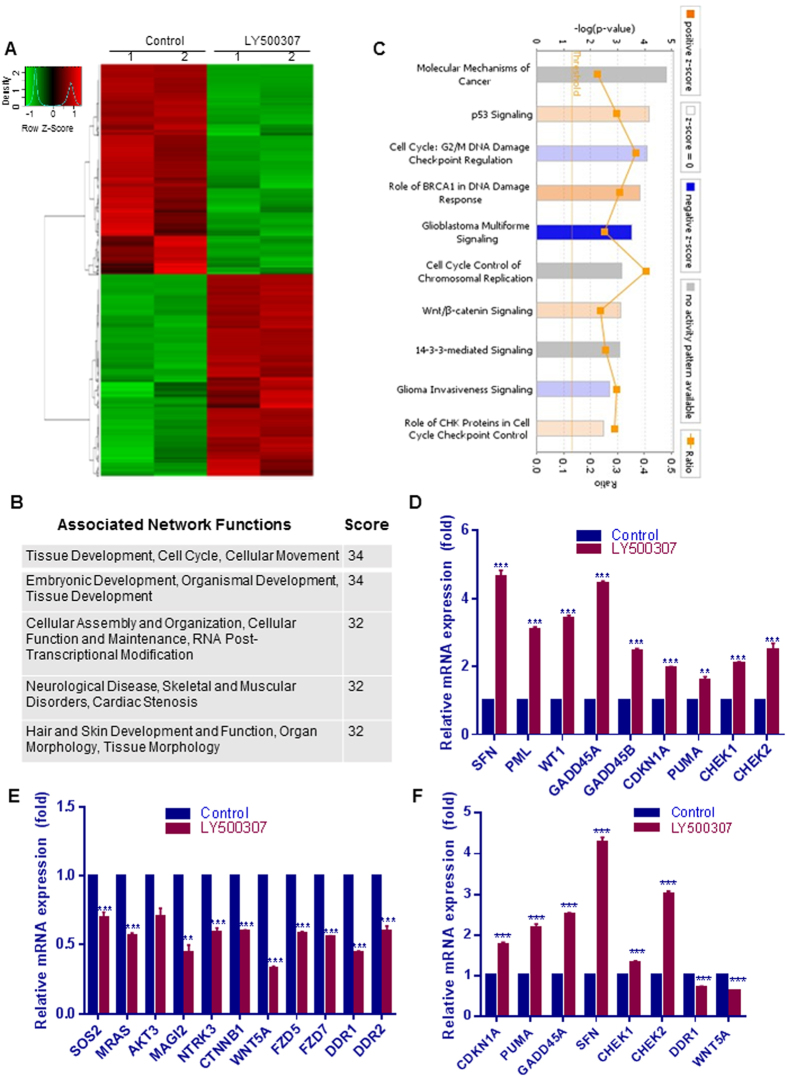
Analysis of global transcriptome changes modulated by LY500307. Total RNA was isolated from U87 cells that were treated with either vehicle or LY500307 for 48 h using RNeasy mini kit and the quality of RNA was tested using a Bio-analyzer and subjected to RNA sequencing. (**A**) heat map of differentially expressed genes between vehicle and LY500307 is shown. (**B**) Differentially expressed genes were subjected to pathway analysis using IPA software and the top five associated network functions of differentially expressed genes are shown. (**C**) The selected top canonical pathways are shown. (**D**–**E**) U87 cells that were treated with either vehicle or LY500307 for 48 h and differentially expressed genes that were upregulated or downregulated were validated using qRT-PCR. F, U251 cells were treated with either vehicle or LY500307 for 48 h and differentially expressed genes were validated using qRT-PCR.

To identify the global changes in gene expression following LY500307 treatment in GBM cells, we performed global transcriptome analysis. U87 cells were treated with either vehicle or LY500307 for 48 h, and the isolated RNA was subjected to RNA-seq analysis. Genes that had at least a 1.5-fold change in expression (p < 0.01) were chosen for analysis. Overall, 3204 genes were differentially expressed in LY500307-treated U87 cells; 1568 genes were upregulated and 1636 genes were downregulated. A representative heat map was shown in Fig. 3a. The complete list of differentially expressed genes is given in Supplementary Table 1. To further examine the biological significance of the differentially expressed genes, IPA analysis was performed. The top networks that were differentially expressed after LY500307 treatment were related to tissue development, cell cycle, cellular movement, cellular assembly and organization, cellular function and maintenance (Fig. 3b). Analysis of molecular and cellular functions of differentially expressed genes revealed that they are involved in cellular growth, proliferation, cell death and survival (Supplementary Fig. 4). The canonical pathways that were modulated by LY500307 include cell cycle, DNA damage, p53 signaling, and checkpoint regulation (Fig. 3c). In addition, pathways related to cancer such as molecular mechanism of cancer, GBM signaling, Wnt signaling and glioma invasion were also altered. Ability of LY500307 to modulate these pathways were independently validated by using qRT-PCR on selective genes in U87 (Fig. 3d,e), U251 (Fig. 3f) and patient derived GBM (GBM10) cells (Supplementary Fig. 5). Collectively, these results suggest that LY500307 modulates the expression of genes involved in cell cycle, cell death, survival, and DNA damage response.

### LY500307 induces G2/M cell cycle arrest

**Figure 4:**
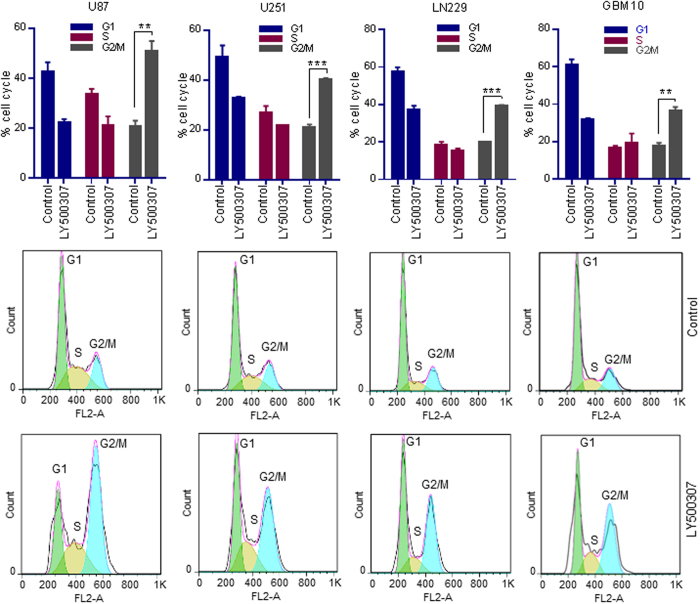
LY500307 promotes G2/M accumulation of GBM cells. U87, U251, LN229 and primary GBM10 cells were treated with either vehicle or LY500307 for 24 h, and the cells were fixed in 70% ethanol and subjected to PI staining for 20 min. Cell cycle distribution was analyzed using flow cytometry. Data are represented as mean ± SE. **p < 0.01, ***p < 0.001.

Since RNA-seq results demonstrated cell cycle as the top network modulated by LY500307, we examined the effect of LY500307 on cell cycle distribution of GBM cells. Flow cytometry analysis of PI-stained cells revealed that LY500307 treatment significantly increased the percentage of cells in G2/M phase in U87, U251, LN229 and GBM10 cells when compared to vehicle (Fig. 4), further supporting the RNA-seq pathway analysis of cell cycle and G2/M DNA damage checkpoint regulation.

### LY500307 sensitizes GBM cells to chemotherapeutic agents

**Figure 5:**
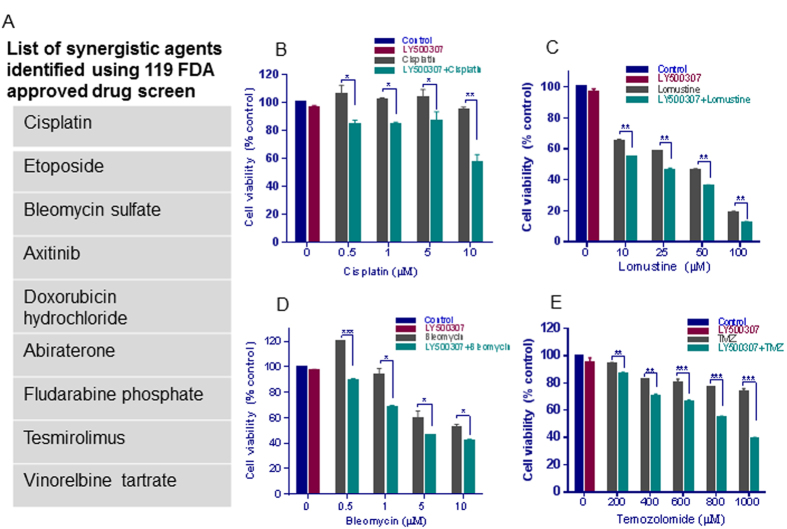
LY500307 sensitizes GBM cells to chemotherapeutic agents. (**A**) U87 GBM cells were treated with either vehicle or LY500307 for 24 h and then treated with one of 119 different FDA-approved drugs for an additional 72 h. Cell viability was determined using an MTT assay, and the list of the drugs that had synergistic activity in the presence of LY500307 are represented in Fig. 5A. U251 GBM cells were pretreated with LY500307 for 48 h followed by treatment with varying doses of cytotoxic drugs cisplatin (**B**) lomustine (**C**) or bleomycin (**D**) for an additional 96 h. Cell viability was determined using MTT assay. (**E**) U138 cells were pretreated with LY500307 for 72 h followed by treatment with varying doses of temozolomide for additional 5 days. Cell viability was determined using an MTT assay.

Emerging data provide the evidence that using a multi-targeted approach has an advantage over using a single agent for GBM therapy, and that sensitizer drugs that enhance the utility of chemotherapy are advantageous. We performed an initial *in vitro* screen of 119 FDA-approved drugs in combination with LY500307 on the cell viability of U87 cells. Our results demonstrated that LY500307 sensitized U87 cells to several FDA approved drugs and nine drugs that showed synergism with LY500307 are presented in Fig. 5a. Interestingly, many of the chemotherapeutic agents that LY500307 sensitized cause DNA damage and apoptosis. We independently validated some of the potent compounds from the initial screen including cisplatin, bleomycin. We also tested potent GBM chemotherapeutic agents including lomustine, temozolomide. Our results revealed that LY500307 sensitized GBM cells to cisplatin, bleomycin and lomustine (Fig. 5b–d). Importantly, we also found that LY500307 significantly sensitized temozolomide-resistant cells U138 to temozolomide-mediated reduction in cell viability (Fig. 5e).

### LY500307 reduces GBM progression in an orthotopic model

**Figure 6:**
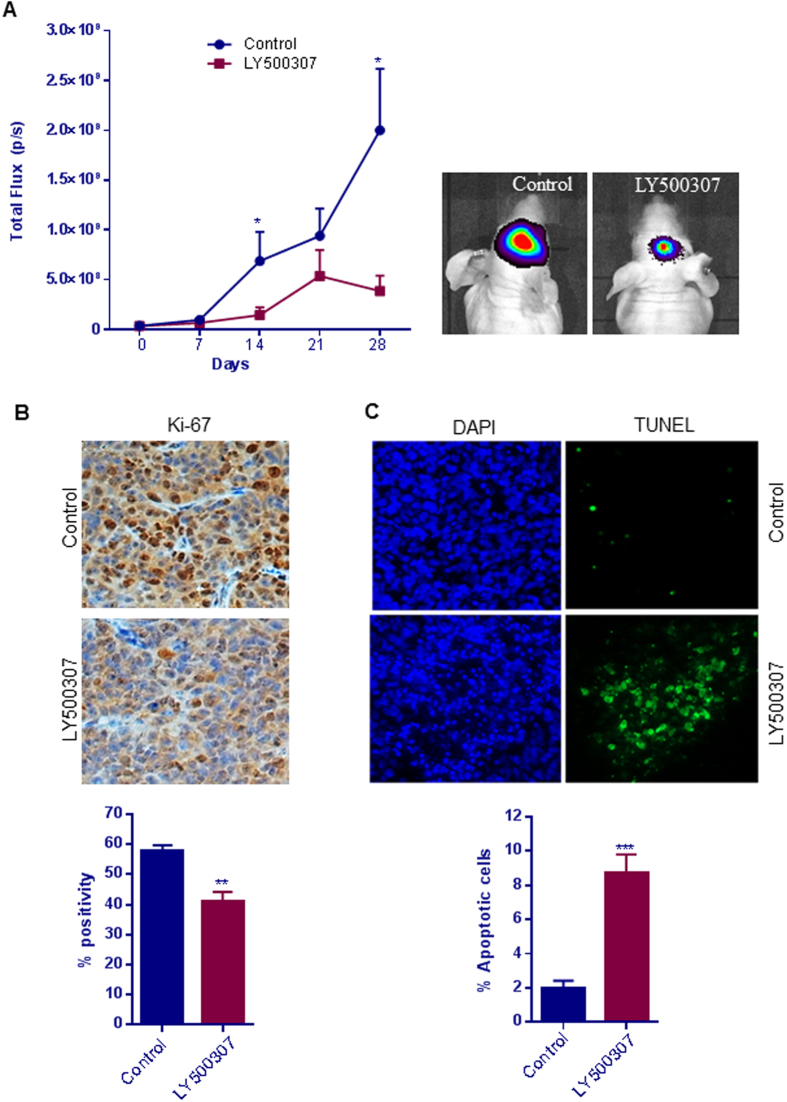
LY500307 reduces GBM progression and induces apoptosis *in vivo*. (**A**) U251 GBM-luc cells were implanted intracranially into the right striatum of nude mice. The mice were then randomized to either the control or the treatment group and received either vehicle or LY500307 (5 mg/kg body weight/day), respectively, for 28 days. Tumor growth in terms of luciferase intensity was measured using Xenogen IVIS imaging (n = 5). (**B**) Mouse brains collected from both the control and LY500307-treated mice were fixed in formalin and subjected to immunohistochemical staining for Ki-67. For quantitation, Ki-67-positive cells from five different fields were counted and plotted as histogram. (**C**) Brain sections were subjected to a TUNEL assay, and the number of TUNEL-positive cells was counted in five different fields and plotted as histogram. DAPI was used to visualize the nuclei. Data are represented as mean ± SE. *p < 0.05, **p < 0.01, ***p < 0.001.

To evaluate the effect of LY500307 on *in vivo* tumor growth, U251 cells that express luciferase reporter were injected orthotopically into the brain of mice. After tumors were established, the mice were randomized into a control group, which received vehicle, and a treatment group, which received LY500307 through oral gavage. GBM progression was measured by monitoring luciferase intensity using Xenogen-IVIS imaging system weekly. Compared to vehicle, LY500037 treatment significantly reduced the GBM progression in the U251 tumor model (Fig. 6a). Immunohistochemical analysis revealed that LY500307 treatment significantly reduced the number of proliferation marker Ki-67-positive cells (Fig. 6b) and increased the number of TUNEL-positive apoptotic cells (Fig. 6c). To further confirm the effect of LY500307 on apoptosis, the expression of Cleaved Caspase-3 and Bcl-2 was examined. As shown in Supplementary Fig. 6a, LY500307 treatment significantly increased the expression of Cleaved Caspase-3 and reduced the Bcl-2 expression in tumors compared to controls. Collectively, these results suggest that LY500307 inhibits the GBM progression *in vivo* and induces apoptosis.

### LY500307 increases survival in syngeneic glioma mouse model

**Figure 7:**
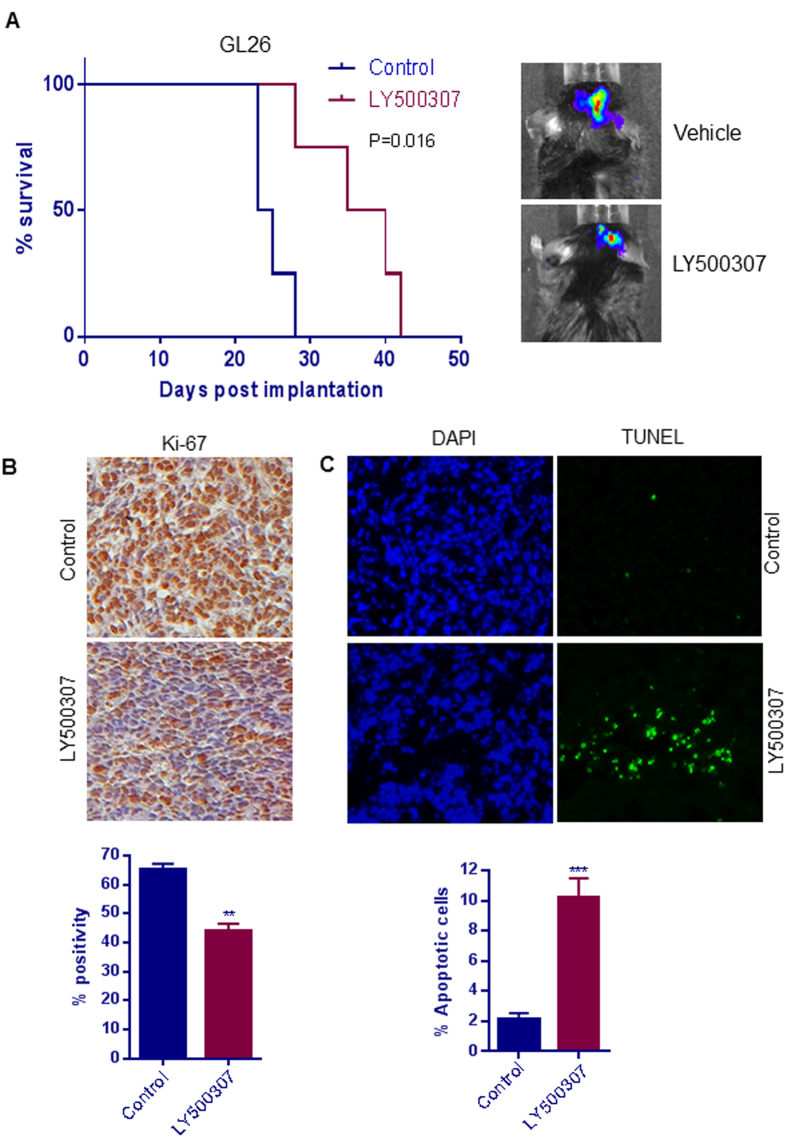
LY500307 prolongs survival of tumor-bearing mice in a syngeneic glioma model. (**A**) GL26 glioma cells were implanted orthotopically into C57BL6 mice that were treated with either vehicle or LY500307 (5 mg/kg body weight/day) orally. Survival of the mice was plotted using Kaplan-Meier curve (n = 4). (**B**) Mouse brains collected from the control and LY500307-treated mice were fixed in formalin and processed for immunohistochemical staining for Ki-67. The number of Ki-67–positive cells from five different fields were counted and plotted as histogram. (**C**) Brain sections were subjected to a TUNEL assay, and number of TUNEL-positive cells was counted for five different fields and plotted as histogram. DAPI was used to visualize the nuclei. Data are represented as mean ± SE. **p < 0.01, ***p < 0.001.

Immune effects during tumor progression play key roles in GBM progression, and GBM-mediated immunosuppression acts as a barrier to the efficacy of chemotherapeutic agents^36^,^37^. To study the anti-tumor activity of LY500307 in the presence of an intact immune system, GL26 cells stably expressing luciferase were implanted into C57BL/6 mice. After establishment of tumors, the mice were randomized into two groups based on luciferase intensity and treated with either vehicle or LY500307 daily by oral gavage. Survival was calculated using Kaplan Meier analysis. Compared to vehicle, LY500307 treatment significantly increased the overall survival of the mice (Fig. 7a). Further, immunohistochemical analysis of tumor sections revealed that LY500307 treatment significantly reduced the expression of the proliferation marker Ki-67 and increased the number of TUNEL-positive apoptotic cells (Fig. 7b,c). Further, the expression of Cleaved Caspase-3 was increased and Bcl-2 expression was decreased significantly in LY500307 treated tumors compared to controls (Supplementary Fig. 6b).

## Discussion

ERβ was initially discovered as second receptor of estrogen^38^, and several studies demonstrated that ERβ expression was downregulated during tumor progression. Ligands that increase ERβ expression or activity will have therapeutic utility. Recently, several groups including ours showed that ERβ expression is reduced during the progression of gliomas and that plant-derived ligands of ERβ exhibit anti-tumor activities^25^,^39^,^40^. However, plant-derived ligands have low efficacy and are difficult to synthesize in large quantities. Thus, more potent synthetic ERβ ligands that work more effectively are urgently needed for clinical application. Our work provides the evidence that LY500307, a synthetic ERβ agonist has potential to specifically reduce the proliferation of GBM cells with high potency, induce apoptosis, promote G2/M cell cycle arrest and sensitize GBM cells to chemotherapeutic agents. Further, our results also demonstrated that LY500307 has the ability to reduce GBM progression *in vivo* and improve the survival of mice.

Selective killing of cancer cells without affecting normal cells is critical for successful chemotherapy. Our results demonstrated that LY500307 selectively reduces the viability of GBM cells with little effect on normal astrocytes. Furthermore, our *in vivo* studies showed no LY500307-related toxicities in various organs (data not shown). Previous studies demonstrated that overexpression of ERβ or activation with ligands results in a decrease in proliferation and induction of apoptosis of cancer cells, and that depending on the cell type, activation of ERβ can promote either G_2_ or G_1_ arrest 14,41. Our results suggested that activation of ERβ with LY500307 induces apoptosis of GBM cells. Earlier studies showed that overexpression of ERβ or activation with ligands causes G2/M arrest in several cancer cells, and that ERβ increases the expression of several genes, including p21 and GADD45A^14^,^41^,^42^,^43^. Our results demonstrate that LY500307 treatment causes cell cycle arrest of GBM cells in G2/M phase and increases the expression of p21 and GADD45A in GBM cells also confirms the earlier observations in other model cells.

ERβ functions as a transcription factor that modulates both classical estrogen response element (ERE)–containing genes and non-ERE genes via non-classical signaling by interacting with AP1, SP1, NF-κB and KLF5 transcription factors^44^,^45^,^46^. Our RNA-seq analysis revealed that LY500307 modulates several genes involved in cell cycle regulation, apoptosis and DNA damage response. Further, LY500307 treatment also down regulated several genes involved in tumor development and progression including Wnt signaling, GBM signaling and glioma invasion signaling. Since ERβ regulates multiple pathways directly or indirectly by binding to the key mediators, it is not surprising to observe the modulation of multiple pathways by LY500307 that includes induction of cell cycle arrest and cell death molecules and suppression of oncogenic signaling molecules. In addition, our results showed that LY500307 promotes ERβ extra-nuclear signaling, leading to activation of the proapoptotic p38 and JNK pathways. Our results showing ERβ knockdown reduced the efficacy of LY500307 to inhibit GBM proliferation further confirms that specific role of ERβ in LY500307 actions.

The current treatment options for GBM are poor, and the mortality rates are very high. Multiple challenges remain, including difficulty of complete resection of GBM, rapid and aggressive tumor relapse and resistance to external radiation and chemotherapy. Therefore, sensitizer drugs that enhance the utility of chemotherapy are urgently needed. Recent studies showed that selective targeting of ERβ with agonists can sensitize malignant pleural mesothelial cells to cisplatin toxicity^19^ and that inhibition of ERβ increased DNA repair that contributes to cisplatin resistance in medulloblastoma cells^47^. Further, ERβ expression influences the malignant pleural mesothelial cell responsiveness to gefitinib^19^. Our results using 119 FDA-approved drugs in combination with a low dose of LY500307 revealed sensitization of GBM cells to several chemotherapeutic agents, including axitinib, doxorubicin, cisplatin, bleomycin and etoposide. Our RNA-seq studies revealed potential of ERβ agonists to down-regulate a number of genes involved in DNA repair and DNA damage response. Most importantly downregulation of DNA repair genes such as DDR1 and DDR2 may provide mechanistic explanation for LY500307-mediated sensitization effects. Further, our studies also revealed that LY500307 sensitizes GBM cells to currently used chemotherapeutic agents temozolomide and lomustine. ERβ agonists’ ability to suppress pathways involved in DNA repair can be exploited in future to promote apoptosis of GBM cells.

We previously reported that the plant-derived ERβ agonist liquiritigenin reduces the growth of subcutaneous glioma xenograft tumors^25^. Recently, salicylketoxime-based estrogen receptor β agonists also reduced the glioma growth in subcutaneous models^48^. However, lack of drug testing using an orthotopic model is a limitation of these studies. Further, the tumor microenvironment and the presence of intact immune system must be considered in efficacy testing of chemotherapeutic drug on GBM growth. In this study, we have tested the effect of LY500307 using both orthotopic tumor models and syngeneic model with intact immune system. Our results demonstrate that the selective ERβ agonist LY500307 reduced GBM progression as well as enhanced survival in syngeneic mouse models. Since LY500307 readily crosses the blood–brain barrier, it is currently being tested in clinical trials and it is well tolerated, it can be readily transferred to clinical use with current chemotherapies, thereby providing an additional tool for enhancing survival in GBM patients with limited toxicity.

## Materials and Methods

### Cell culture, reagents and generation of stable ERβ overexpression and ERβ shRNA cells

Human glioblastoma cell lines U87, U251, LN229 and T98G and the mouse glioma cell line GL26 were obtained from the American Type Culture Collection (ATCC). Cell lines were maintained in DMEM supplemented with 10% fetal bovine serum (Sigma Chemical Co, St. Louis, MO). LY500307 was purchased from Selleckchem (Houston, TX) and Apex Biosciences (Durham, NC). ERβ antibodies were obtained from Santa Cruz Biotechnology (Dallas, TX),GeneTex (Irvine, CA) and Millipore (Billerica, MA), Ki-67 was obtained from Abcam (Cambridge, MA) and Bcl-2 was purchased from Dako (Carpinteria, CA) and Santa Cruz Biotechnology (Dallas, TX). The p-ERK1/2, ERK1/2, p-AKT, AKT, p-p38MAPK, p38MAPK, p-JNK,JNK, Cleaved Caspase-3 and GAPDH antibodies were obtained from Cell Signaling Technology (Beverly, MA). ERβ-specific short hairpin RNA (shRNA) lentiviral plasmids, β-actin and all secondary antibodies were purchased Sigma from Chemical Co (St. Louis, MO). Normal human astrocytes were obtained from ScienCell Research Laboratories (Carlsbad, CA) and maintained in astrocyte medium. Stably ERβ-expressing GBM cells were generated by infecting them with pLenti6/V5-D-FLAG ERβ and empty control vectors and positive cells were selected with blasticidine (5 μg/mL). GBM cells stably expressing ERβ-shRNA were generated by infecting cells with human-specific lentiviral ERβ-shRNA particles and selected with puromycin (1 μg/mL), and pooled clones were used for all the studies. Lentiviral particles expressing nontargeted shRNA were used to generate control.

### Primary GBM cells

Primary GBM cells were isolated from discarded GBM tumor specimens after approval from Office of the Institutional Review Board (IRB) at UT Health Science Center at San Antonio. All the discarded tumor specimens were obtained from patients undergoing surgery after informed consent, and no clinical linkers or codes for the specimens accessible to any research personnel or the PI. All the methods were carried out in accordance with IRB approved guidelines. Briefly, fresh tumor tissue was collected intraoperatively, dispersed into single cells, and cultured briefly in a neurobasal media for expansion and purification followed by intracranial injection in nude mice. Patient-derived GBM neurosphere lines were maintained in Neurobasal Medium supplemented with B27 serum-free supplement and growth factors EGF (20 ng/mL), bFGF (20 ng/mL) and LIF (10 ng/mL) as described^49^. The resulting tumors were characterized for consistency by histology with the primary tumor, growth attributes including time to animal demise, and for gene expression profiling for the molecular subtype. GBM line 101310 (GBM10) used in the assays is grade IV GBM, MGMT hypomethylated and average survival time in mice is 108 days.

### Cell viability and colony formation assays

The effect of LY500307 on cell viability of GBM cells and normal astrocytes was assessed by using MTT assays. For LY500307 treatment, GBM cells were maintained in phenol red–free DMEM containing 5% dextran-charcoal treated FBS. Briefly, GBM cell lines and normal astrocytes were seeded in 96 well plates (2 × 10^3^ cells/well). After overnight incubation, cells were treated with either vehicle or varying concentrations of LY500307 for 72 h. MTT was added to each well and incubated for 4 h. Formazan crystals were solubilized in DMSO and the optical density was measured using micro-plate reader. The effect of LY500307 on the viability of patient-derived primary GBM lines and the cell proliferation rates of control and ERβ-overexpressing GBM cells were measured using CellTiter-Glo Luminescent Cell Viability Assay (Promega, Madison, WI) in 96-well, flat, clear-bottom, opaque-wall microplates according to manufacturer’s instructions. Total ATP content as an estimate of total number of viable cells was measured on an automatic Fluoroskan Luminometer. For colony formation assays, U87 and U251 cells (500 cells/well) were seeded in 6 well plates. After overnight incubation, the cells were treated with vehicle or LY500307 (5 μM) for 72 h. Then, cells were washed with PBS and the cells were allowed to grow for additional 7 days. The cells were fixed in ice-cold methanol and stained with 0.5% crystal violet solution. Colonies that contained ≥50 cells were counted.

### Annexin V apoptosis assay

The apoptotic effect of LY500307 on GBM cells was analyzed using the Annexin V/PI kit as per the manufacturer’s instructions (BioLegend, San Diego, CA). Briefly, U87 and U251 GBM cells were seeded in 60-mm culture plates and treated with either vehicle or LY500307 (5 μM) for 48 h. Cells were harvested at a density of 2.5 × 10^6^ cells/mL in Annexin V binding buffer and 100 μL of cell suspension was incubated with Annexin V FITC and propidium Iodide (PI) for 15 min at room temperature in the dark. Then, 400 μL of Annexin V binding buffer was added to each sample and stained cells were analyzed using flow cytometry.

### Cell cycle analysis

U87, U251, LN229 and GBM10 (101310) cells were seeded in 100-mm culture plates, and after overnight incubation, cells were treated with either vehicle (0.1% DMSO) or LY500307 (5 μM) for 24 h. Cells were then trypsinized and harvested in PBS, followed by fixation in ice-cold 70% ethanol for 30 min at 4 °C. Cells were washed again with PBS and stained with a mixture of 50 μg/mL propidium iodide and 50 μg/mL RNase A. The PI-stained cells were subjected to flow cytometry using a FACS Calibur (BD Biosciences).

### Reporter gene assays

GBM cells were maintained in phenol red–free DMEM supplemented with 5% dextran-charcoal–stripped serum for 48 h prior to transfection. Cells were transiently transfected with 250 ng of ERE-Luc, AP1-Luc, SP1-Luc, or NF-κB-Luc reporter plasmids using Turbofect transfection reagent (Thermo Scientific, Waltham, MA). After 24 h, cells were treated with either vehicle or LY500307 (1 μM) for additional 24 h. β-galactosidase reporter (50 ng) plasmid was co-transfected and used for data normalization. Cells were lysed in Passive Lysis Buffer, and Luciferase activity was measured using the luciferase assay system (Promega, Madison, WI) in luminometer.

### Western blotting

Whole cell lysates were prepared from GBM cells using RIPA buffer (Sigma, St Louis, MO) containing protease and phosphatase inhibitors. Total proteins (50 μg) were mixed with SDS sample buffer and separated on SDS-PAGE gels. Resolved proteins were then transferred onto nitrocellulose membranes, blocked with 5% non-fat dry milk powder for 1 h at room temperature and incubated with respective primary antibodies over night at 4 °C followed by secondary antibody incubation for 1 h at room temperature. Blots were developed using the ECL kit (Thermo Scientific, Waltham, MA).

### *In vitro* screening of FDA-approved drugs in combination with LY500307 on GBM cells

All the FDA-approved anti-cancerous drugs were obtained from NCI at 10 mM concentration (https://dtp.cancer.gov/organization/dscb/obtaining/available_plates.htm). The initial screen of the combination treatment of the 119 FDA-approved drugs and LY500307 was performed on U87 cells using MTT assay. U87 cells (1 × 10^3^/well) were seeded in 96 well plates. After an overnight incubation, the cells were treated with LY500307 (1 μM) and varying doses of each FDA-approved drug at final concentrations of 0.1 μM, 1 μM, and 10 μM for 72 h in phenol red–free DMEM that contains dextran-charcoal treated FBS. Some of the compounds that sensitized GBM cells with LY500307 were further validated using U251 cells. U251 cells were seeded in 96 well plates (1 × 10^3^ cells/well) in phenol red–free DMEM containing dextran-charcoal treated FBS. After an overnight incubation, the cells were pretreated with vehicle or LY500307 (1 μM) for 48 h followed by wash-off and replenished with varying concentrations of each drug alone or in combination with LY500307 for 96 h. Cell viability was then measured using the MTT assay as described above. The temozolomide-resistant cell line U138 was subjected to pretreatment with LY500307 for 72 h followed by varying doses of temozolomide for 5 days, and then cell viability was measured using the MTT assay.

### RNA sequencing and qRT-PCR

U87 GBM cells were treated with either vehicle or LY500307 (5 μM) for 48 h, and total RNA was isolated using RNAesy mini kit (Qiagen) according to the manufacturer’s instructions. The purity of prepared RNA was determined using Agilent 2100 BioAnalyzer. Illumina TruSeq RNA Sample preparation was performed following manufacturer’s protocol, and the samples were run on an Illumina HiSeq 2000 in duplicates. The combined raw reads were aligned to UCSC hg19, and the genes were annotated by Tophat. Genes were annotated and quantified by HTSeq-DESeq pipeline. Differential expression analysis was performed by DEseq and significant genes with at least 1.5-fold change with p < 0.01 were chosen for analysis. The interpretation of biological pathways using RNA-seq data was performed with ingenuity pathway analysis (IPA) software using all significant and differentially expressed genes. To validate the selected genes, reverse transcription (RT) reactions were performed by using SuperScript III First Strand kit (Invitrogen, Carlsbad), according to the manufacturer’s protocol. Real-time PCR was done using SYBR Green (Thermo Scientific) on an Illumina Real-Time PCR system with the following primers. Results were normalized to β-actin transcript levels, and the difference in fold expression was calculated using delta-delta-CT method.

Actin-F-5′- GTGGGCATGGGTCAGAAG-3′; Actin-R-5′- TCCATCACGATGCCAGTG-3′

ESR2-F-5′- GGC AGA GGA CAG TAA AAG CA -3′; ESR2-R-5′- GGA CCA CAC AGC AGA AAG AT -3′

IL24-F-5′- CTTTGTTCTCATCGTGTCACAAC-3′; IL24-R-5′- TCCAACTGTTTGAATGCTCTCC-3′

CDKN1A-F-5′- CTGGAGACTCTCAGGGTCGAAA-3′; CDKN1A-R-5′-GATTAGGGCTTCCTCTTGGAGAA-3′;GADD45A-F-5′- GGTGTACGAAGCGGCCAA-3′; GADD45A-R-5′- GCAGGCACAACACCACGTTA-3′

PUMA-F-5′- ATGCCTGCCTCACCTTCATC-3′; PUMA-R-5′- TCACACGTCGCTCTCTCTAAACC-3′

CTNNB1-F-5′- AAAATGGCAGTGCGTTTAG-3′; CTNNB1-R-5′- TTTGAAGGCAGTCTGTCGTA-3′

SFN-F-5′- TGACGACAAGAAGCGCATCAT-3′; SFN-R-5′- GTAGTGGAAGACGGAAAAGTTCA-3′

AKT3-F-5′-TGTGGATTTACCTTATCCCCTCA-3′; AKT3-R-5′-GTTTGGCTTTGGTCGTTCTGT-3′

PML-F-5′- CGCCCTGGATAACGTCTTTTT-3′; PML-R-5′- CTCGCACTCAAAGCACCAGA-3′

CHEK1-F-5′- ATATGAAGCGTGCCGTAGACT-3′; CHEK1-R-5′- TGCCTATGTCTGGCTCTATTCTG-3′

CHEK2-F-5′- TGAGAACCTTATGTGGAACCCC-3′; CHEK2-R-5′- ACAGCACGGTTATACCCAGC-3′

GADD45B-F-5′- TACGAGTCGGCCAAGTTGATG-3′; GADD45B-R-5′- GGATGAGCGTGAAGTGGATTT-3′

WT1-F-5′- CACAGCACAGGGTACGAGAG-3′; WT1-R-5′- CAAGAGTCGGGGCTACTCCA-3′

WNT5A-F-5′- ATTCTTGGTGGTCGCTAGGTA-3′; WNT5A-R-5′- CGCCTTCTCCGATGTACTGC-3′

SOS2-F-5′- ATGTAGAGGAGCGAGTTCAGAA-3′; SOS2-R-5′- ATGGTAGTCCACTTTGTACCCT-3′

FZD5-F-5′- CATGCCCAACCAGTTCAACC-3′; FZD5-R-5′- CGGCGAGCATTGGATCTCC-3′

FZD7-F-5′- GTGCCAACGGCCTGATGTA-3′; FZD7-R-5′- AGGTGAGAACGGTAAAGAGCG-3′

NTRK3-F-5′- ACGAGAGGGTGACAATGCTG-3′; NTRK3-R-5′- CCAGTGACTATCCAGTCCACA-3′

DDR1-F-5′- AAGGGACATTTTGATCCTGCC-3′; DDR1-R-5′- CCTTGGGAAACACCGACCC-3′

DDR2-F-5′- GCTATATGCCGCTATCCTCTGG-3′; DDR2-R-5′- ACTCTGACCACTGACTGGAAG-3′

MAGI2-F-5′- TCCGGCTCAAGTGTGTCAAG-3′; MAGI2-R-5′- AGGTTGTCACGAATGATTTGCT-3′

MRAS-F-5′ TTCCTCATCGTCTACTCCGTC-3′; MRAS-R-5′ AGGATCATCGGGAATGACTCC-3′

### Immunohistochemistry

Immunohistochemical studies were performed as described previously^25^. Coronal brain sections were incubated in xylene and passed through series of graded alcohols and then subjected to antigen retrieval using the antigen retrieval solution (Vector Lab, Inc. CA). Tissue sections were incubated in 3% H_2_O_2_ solution for 20 min and then subjected to blocking using the vector lab blocking kit. Tissue sections were incubated overnight with Ki-67 (1:100), Cleaved Caspase-3 (1:200) and Bcl-2 (1:50) and then with secondary antibodies for 45 min at room temperature. Immunoreactivity was visualized by using the DAB substrate and counterstained with haematoxylin (Vector Lab, Inc. CA). The proliferative index was calculated as percentage of Ki-67-positive cells in five randomly selected microscopic fields at 20X per slide. TUNEL analysis was performed using the *In situ* Cell Death Detection Kit (Roche, Indianapolis, IN) as per the manufacturer’s protocol, and five randomly selected microscopic fields in each group were used to calculate the relative ratio of TUNEL-positive cells. DAPI was used to visualize the nuclei.

### *In Vivo* Orthotopic Tumor Models

All animal experiments were performed after obtaining UTHSCSA IACUC approval, and all the methods were carried out in accordance with the IACUC approved guidelines. U251 and GL26 cells labelled with GFP-Luciferase (1 × 10^6^) were injected orthotopically into the mouse brain at 2 mm right, 2 mm posterior and 3 mm deep from bregma into the striatum. GL26 mice were ovariectomized bilaterally to avoid the effects of circulated estrogen. Five days after injection, the mice were imaged using Xenogen IVIS system and randomized to receive either control or treatment. The control group received vehicle (30% captisol) and the treatment group received LY500307 in 30% captisol (5 mg/Kg body weight/day) orally. Tumor growth was measured weekly using Xenogen IVIS system. After treatment, the mice were euthanized, and their brains were isolated and processed for histological studies.

### Statistical analyses

Statistical differences between groups were analyzed with either t-test or ANOVA as appropriate using GraphPad Prism 6 software (GraphPad Software, SanDiego, CA). Student t-test was used to assess the statistical difference between control and LY500307-treated groups. Mice survival was determined using Kaplan-Meier survival curve. All the data represented in bar graphs are shown as means ± SE. A value of p < 0.05 was considered as statistically significant. RNA-seq data was analyzed using IPA software.

## Additional Information

**How to cite this article**: Sareddy, G. R. *et al.* Selective Estrogen Receptor β Agonist LY500307 as a Novel Therapeutic Agent for Glioblastoma. *Sci. Rep.* **6**, 24185; doi: 10.1038/srep24185 (2016).

## Supplementary Material

Supplementary Information
