# Supplementary material for: Selective Estrogen Receptor β Agonist LY500307 as a Novel Therapeutic Agent for Glioblastoma
Source: Sci Rep. 2016 Apr 29;6:24185. doi: 10.1038/srep24185 (PMC4850367; doi:10.1038/srep24185)
Supplement: Supplementary Information [file srep24185-s1.pdf]

Selective Estrogen Receptor β Agonist LY500307 as a Novel Therapeutic Agent for Glioblastoma

Gangadhara R Sareddy[1], Xiaonan Li[1], Jinyou Liu[1], Suryavathi Viswanadhapalli[1], Lauren Garcia[1], Aleksandra Gruslova[2], David Cavazos[2], Mike Garcia[2], Anders M Strom[3], Jan-Ake Gustafsson[3], Rajeshwar Rao Tekmal[1], Andrew Brenner[2, 4], and Ratna K. Vadlamudi[1,2*]

[1]The Department of Obstetrics and Gynecology, [2]Cancer Therapy & Research Center, [4] Hematology & Oncology, University of Texas Health Science Center at San Antonio, San Antonio TX 78229
[3]University of Houston, Houston, TX 77004

Supplementary figure 1

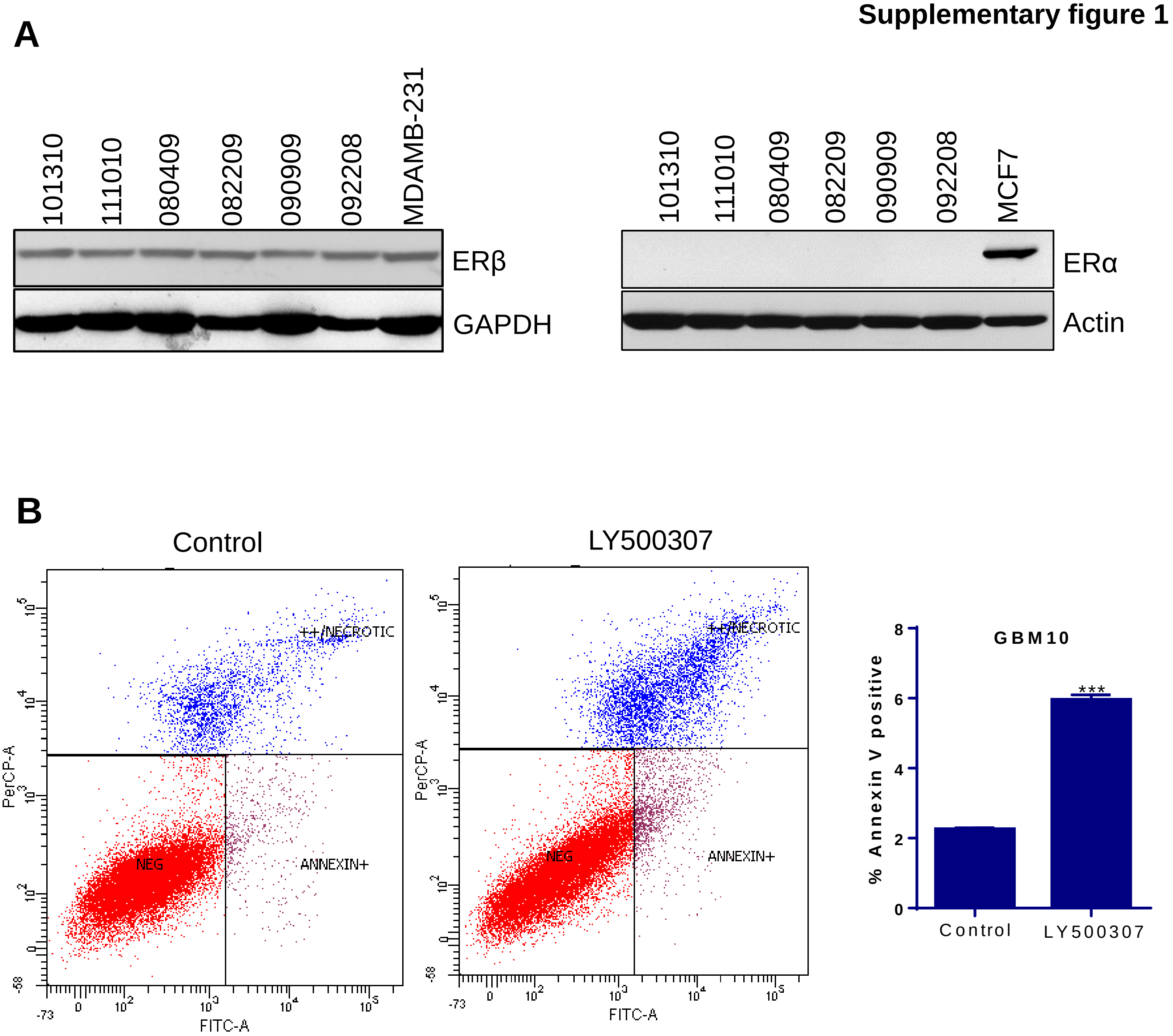

Supplementary figure 1. A, The expression of ERβ and ERα was examined using western blotting in various patient derived glioblastoma cell lines. MDAMB-231 and MCF7 cells were used as positive controls for ERβ and ERα respectively. B, Primary glioblastoma GBM10 (101310) cells were treated with either vehicle or LY500307 for 72 h followed by Annexin V-FITC and Propidium Iodide (PI) staining for 15 min. The Annexin V–positive apoptotic populations were determined using flow cytometry. Data are represented mean ± SE. ***$p<0.001$.

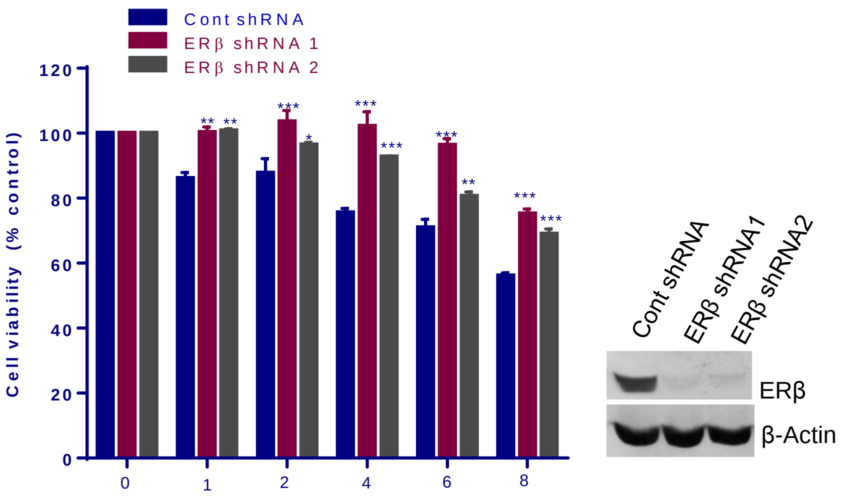

Supplementary figure 2. U251 cells were stably transfected with either control shRNA or ERβ shRNA1 or ERβ shRNA2 and the efficiency of ERβ knockdown was confirmed by Western blotting. U251 control shRNA and ERβ shRNA cells were treated with either vehicle or LY500307 for 72 h, and the cell viability was determined using an MTT assay. Data are represented mean ± SE. **p<0.01, ***p<0.001.

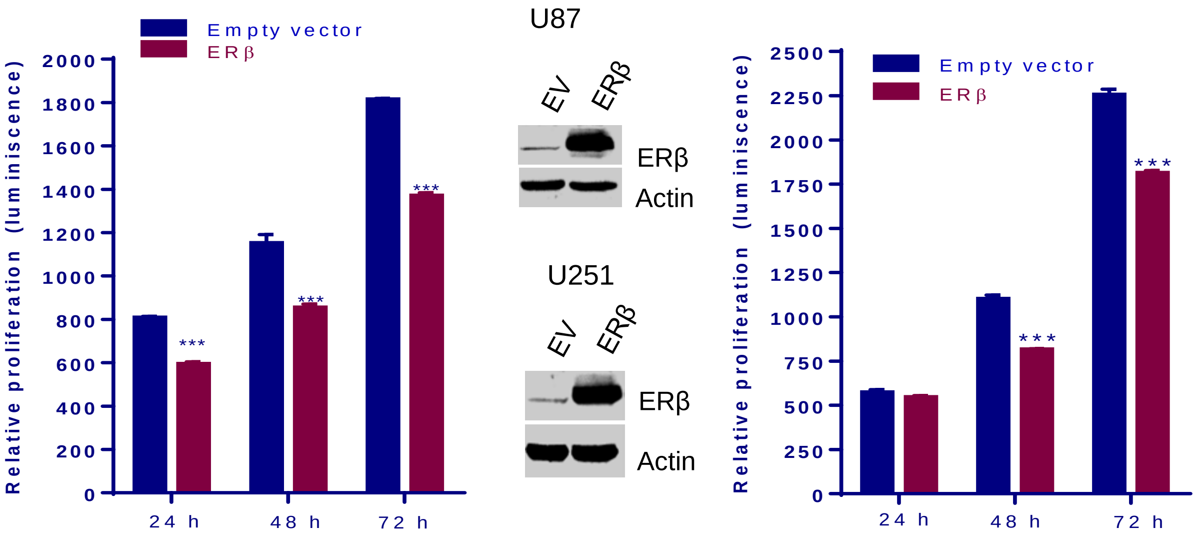

Supplementary figure 3. U87 and U251 cells were transfected with either empty or ERβ expression plasmids and cell proliferation rates were measured using CellTiter-Glo luminescent assay. The expression of ERβ was confirmed by western blotting. Data are represented mean ± SE. ***p<0.001.

Top Molecular and Cellular Functions	p-value	#molecules
Cellular Growth and Proliferation	5.69E-06 – 4.65E-31	1058
Cell Death and Survival	5.39E-06 – 4.19E-25	950
Cellular Movement	5.43E-06 – 1.86E-24	627
Cellular Development	5.84E-06 – 1.76E-21	996
Cellular Assembly and Organization	4.90E-06 – 9.05E-18	467

Supplementary figure 4. The differentially expressed genes were subjected to IPA analysis and the top five molecular and cellular functions of differentially expressed genes were shown.

Supplementary figure 5

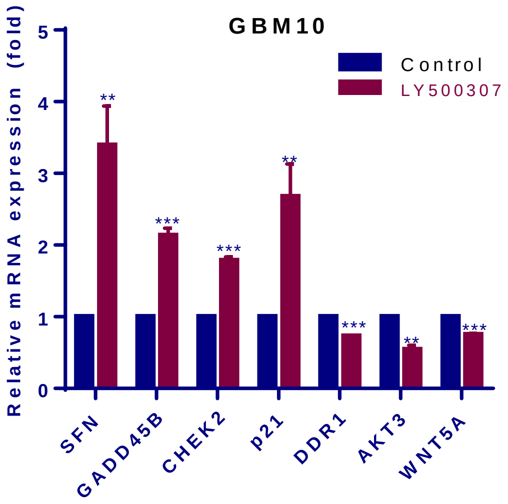

Supplementary figure 5. GBM10 cells were treated with either vehicle or LY500307 for 48 h and differentially expressed genes that were upregulated or downregulated were validated using qRT-PCR. Data are represented mean ± SE. **p<0.01, ***p<0.001.

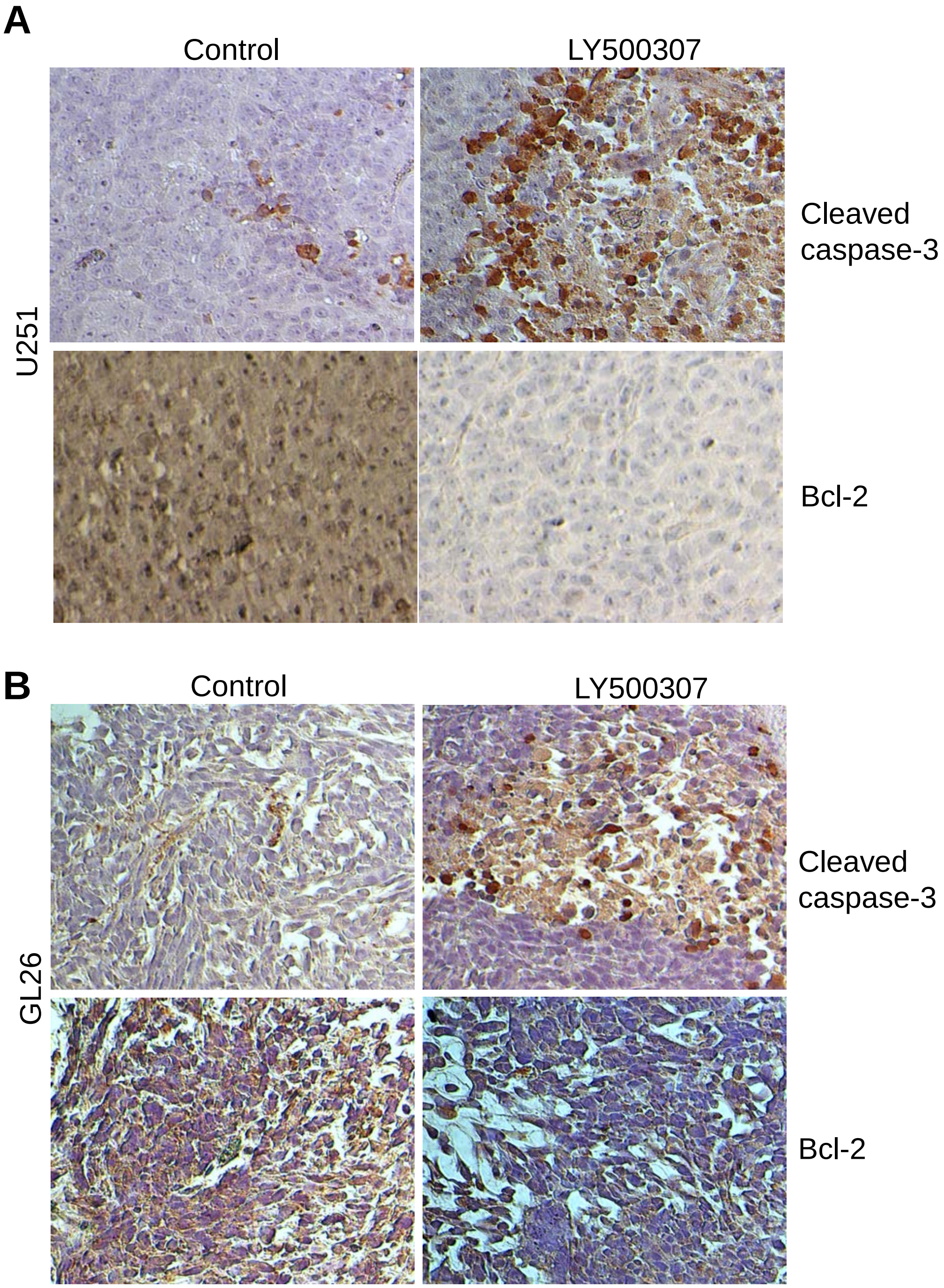

Supplementary figure 6. Mouse brains collected from both the control and LY500307-treated mice implanted with U251 (A) and GL26 (B) were fixed in formalin and subjected to immunohistochemical staining for Cleaved caspase-3 and Bcl-2 as described in materials and methods.

Table 1: Differentially regulated genes upon LY500307 treatment

gene id	U87-control	U87-LY500307	foldChange	p-value
AIM2	0	17.43040604	Inf	7.49E-06
CSF2	0	15.66046389	Inf	2.70E-05
IL1RL1	0	17.06461913	Inf	1.01E-05
LOC100216	0	12.72943558	Inf	0.0002015
PNLIPRP3	0	22.95498866	Inf	2.41E-06
ROBO4	0	20.42279325	Inf	9.91E-07
LRRC15	0.526822718	53.97148844	102.447155	1.68E-15
IL8	19.94705199	1472.505933	73.8207297	5.48E-262
IL33	0.523384085	26.86302641	51.3256463	1.02E-07
MSMP	0.523384085	25.03172535	47.8266842	3.49E-07
FABP4	2.10385224	97.13688071	46.1709615	1.19E-24
NCRNA003	0.523384085	21.0623287	40.2425853	8.19E-05
CXCL11	6.308118085	249.7343439	39.5893578	2.43E-41
ABCC9	17.83288385	603.5744519	33.8461495	6.52E-118
DNAH17	0.526822718	16.1489686	30.653516	0.0001199
DMBT1	2.100413606	55.68007168	26.5090987	1.78E-13
ARC	0.523384085	13.70644501	26.1881196	0.0005597
UGT3A2	0.523384085	13.30997866	25.430614	0.0007423
MMP1	1.570152254	38.92224708	24.7888362	1.37E-09
C3AR1	6.290924917	152.9703497	24.3160349	3.79E-33
MMP3	2.627236324	62.94628354	23.959125	1.02E-14
MYPN	0.526822718	12.33296922	23.4100937	0.0013647
CXCL10	129.7056981	2950.824395	22.7501524	8.94E-301
RP1	0.523384085	11.81378505	22.5719226	0.0018646
CYP26A1	0.523384085	11.75242614	22.4546876	0.0018936
CCL20	3.150620409	70.39420559	22.3429663	4.78E-16
MUC13	0.523384085	11.44799815	21.8730345	0.0024477
CXCL9	0.526822718	11.41731869	21.6720318	0.0024642
KCNF1	3.674004494	79.00321379	21.5032981	1.22E-17
TNFAIP6	6.304679451	133.1280995	21.1157602	3.28E-28
CALB1	9.978683945	191.6164817	19.2025805	3.29E-38
EREG	26.77167689	496.2096516	18.5348738	5.98E-86
HSPA6	3.667127227	63.86193407	17.4147037	9.27E-14
C2orf78	1.570152254	26.86302641	17.1085488	2.03E-06
RCSD1	1.573590888	25.42819171	16.159341	4.76E-06
SELE	1.050206803	16.60679387	15.8128797	0.0003204
MC4R	1.053645437	16.42271715	15.5865689	0.0003341
POSTN	1.050206803	15.72182279	14.9702161	0.0005578
DLL4	1.046768169	14.10291137	13.4728126	0.0013583
CCL5	14.20014296	190.1509675	13.3907784	1.02E-33
FRAS1	1.050206803	13.70644501	13.0511867	0.0017642
ZBP1	3.150620409	40.5411585	12.8676747	7.20E-05
OASL	790.774775	10071.5138	12.7362608	0
MMP13	3.667127227	46.4905204	12.6776404	2.17E-07

CXCL2	7.358324888	92.86068953	12.6198137	2.02E-17
KCNE1L	1.053645437	13.18726084	12.515843	0.002351
VGF	4.193949945	52.01746957	12.4029782	2.57E-10
CA9	1.050206803	12.85215339	12.2377358	0.0030118
TGM2	2.616920424	31.62535577	12.0849513	1.14E-06
CXCL3	11.01857485	127.3557147	11.5582747	2.60E-22
RGS4	650.4982361	7085.104033	10.8918113	8.75E-292
HSH2D	3.677443127	39.7151798	10.7996721	8.82E-08
HES7	3.150620409	33.57937464	10.6580198	1.02E-06
EVI2B	8.408531691	89.29012578	10.6189914	1.07E-15
RSAD2	130.7318345	1348.574484	10.3155784	5.52E-139
HBEGF	92.41819866	943.6939157	10.2111265	1.42E-109
IDO1	28.35558368	277.3974026	9.78281406	6.34E-41
LOC286002	1.570152254	15.17195917	9.6627312	0.0019221
CMPK2	116.5385688	1119.253467	9.60414632	7.74E-119
NTS	3.147181775	29.76337526	9.45715163	8.15E-06
LOC152225	3.147181775	29.27487054	9.3019319	1.06E-05
CEACAM1	2.093536339	19.38442491	9.25917766	0.0004278
AQP3	2.620359057	23.68892901	9.04033703	9.61E-05
ETV4	50.90924019	460.0367226	9.03640912	7.44E-60
BEST3	3.677443127	32.08318103	8.72431739	5.33E-06
SLCO4A1	7.358324888	63.98465188	8.69554591	1.05E-10
GPRC5A	57.7235492	501.923044	8.69529076	1.01E-62
LAMC2	9.441545326	82.02391392	8.68755178	2.46E-13
GBP4	59.29714008	512.0825051	8.63587189	1.30E-63
SERPIND1	2.627236324	22.55852231	8.58640774	0.0001649
MX2	92.41819866	781.6968782	8.45825703	2.41E-86
LY6E	38.36521533	322.2713781	8.40009304	5.67E-43
LOC10049[illegible]	2.10385224	17.55312385	8.34332541	0.0011737
BCL2A1	2.107290873	17.43040604	8.27147607	0.0011995
NGFR	7.358324888	60.80818795	8.26386289	4.75E-10
F2RL1	2.623797691	21.58151287	8.22529608	0.0002744
BATF2	21.01101333	168.3830114	8.01403572	4.55E-24
PTGS2	10.50206803	83.94725333	7.99340217	4.58E-13
XDH	5.770979466	46.03269514	7.97658273	6.35E-07
FYB	8.914722608	69.69331123	7.81777676	6.25E-11
GDF15	169.6857679	1316.786265	7.7601456	6.60E-95
TNFRSF9	14.68226344	112.9483753	7.69284489	1.49E-16
CD68	183.734611	1406.147216	7.6531428	4.00E-119
KIF21B	3.147181775	23.50485229	7.46853978	0.0002139
TRPV2	12.60248164	93.99109623	7.45814189	6.94E-14
KDR	6.814309002	50.58263487	7.42300281	4.05E-08
OAS2	2061.72172	15205.61114	7.37520053	4.83E-253
KRT34	2.634113591	19.29238655	7.32405262	0.0009306
DHRS2	25.22215644	184.1072007	7.29942347	1.33E-24
NT5E	1084.695134	7917.312721	7.29911334	1.62E-225
ELOVL3	3.674004494	26.70962914	7.26989561	0.0004462

TMEM171	5.777856733	41.66919867	7.21187814	8.34E-07
FST	127.6259163	917.5671962	7.18950526	3.17E-87
CPA4	123.3838255	885.6019999	7.17761827	4.17E-85
CLEC7A	7.878270339	56.41401203	7.16071036	9.97E-09
ISG15	618.058027	4397.00288	7.1142234	6.55E-38
SIDT1	4.72764993	33.45665683	7.07680503	1.16E-05
CGA	2.620359057	18.49945383	7.05989272	0.0014776
KCNJ12	4.724211297	33.3056261	7.04998655	1.39E-05
OAS1	714.6365011	4980.3671	6.96909141	2.40E-193
IFIT2	1316.387806	8986.323393	6.82650155	8.30E-219
SLC14A1	17.31981567	117.7107047	6.7963024	5.30E-16
NGF	5.261349916	35.53339351	6.75366476	8.72E-06
SHC4	51.45669471	347.2417446	6.7482326	4.50E-40
CCDC73	5.247595381	35.16760661	6.70166125	1.08E-05
SOX30	3.67056586	24.51254118	6.67813686	0.0002614
RPSAP52	18.89340655	125.9232465	6.66493076	9.58E-17
IFIT3	2434.903465	16184.99176	6.64707738	3.42E-233
EOMES	7.358324888	48.56725709	6.60031431	2.07E-07
TNFSF10	510.8413631	3344.120134	6.54629867	3.70E-135
DNAH3	3.150620409	20.30007544	6.44319937	0.0011196
SYT16	3.140304508	19.75021181	6.28926646	0.0014338
NCF2	8.411970324	52.04814902	6.18739095	1.51E-07
SCG2	2986.427091	18473.89962	6.18595367	1.80E-221
ARHGDIB	8.921599875	55.00985678	6.16591839	7.34E-08
PHLDA2	31.51995862	193.8135696	6.14891574	4.06E-23
COL13A1	31.51651999	191.2152822	6.06714454	7.36E-17
HMGA1	2927.046	17649.06346	6.02965019	3.95E-215
RAET1L	6.838379437	39.89688998	5.83426093	0.0001221
DIRAS3	231.0454967	1340.423301	5.80155563	2.69E-94
EVI2A	7.881708973	45.54655696	5.77876665	1.76E-06
LOC14977	4.207704479	23.75028791	5.64447623	0.0007444
MAB21L1	62.45463776	350.3851625	5.61023448	1.88E-35
GBP5	150.196766	840.9239338	5.59881518	6.28E-64
C1orf116	3.674004494	20.36143434	5.54202761	0.0020277
FOSL1	983.6132334	5448.727249	5.53950177	4.63E-162
UNC80	3.674004494	20.33075489	5.5336772	0.0020365
CTSS	14.17951116	78.33063235	5.52421247	6.27E-10
SPHK1	71.45188757	394.6738619	5.52363101	3.57E-30
CLDN4	35.7173472	196.5322083	5.50243015	1.36E-21
IFIT1	4462.184283	24511.0319	5.49305684	7.92E-202
USP18	353.9678335	1940.856701	5.48314428	1.83E-109
GCM1	4.72764993	25.82465806	5.46247257	0.0004538
IL4I1	4.734527197	25.82465806	5.45453791	0.0004534
GAP43	80.81434433	438.946081	5.43153675	2.76E-41
TGFBI	54.62106966	294.7688163	5.39661376	4.45E-28
MPP2	6.817747636	36.78415149	5.39535246	2.85E-05
SPOCD1	26.77855416	142.6503917	5.3270386	4.25E-16

IRAK2	130.7490277	695.8191858	5.32179243	1.66E-57
SNORA10	3.67056586	19.41510436	5.28940362	0.0031611
ABCG2	4.193949945	22.10069704	5.26966162	0.0014942
SOCS1	5.770979466	30.25187998	5.24207029	0.0001899
WNT7B	194.3707858	1015.737712	5.22577356	5.11E-73
NR1H4	16.79643158	86.63521255	5.15795347	2.86E-10
NMB	23.1079883	119.0841805	5.15337722	1.81E-13
OAS3	2863.745459	14680.02029	5.126161	3.90E-179
LMO2	6.308118085	31.96046322	5.06656071	0.0001415
CHD7	5.7744181	29.09079382	5.03787452	0.0002992
HIST1H2BJ	6.301240818	31.56399686	5.00917165	0.0001732
PRIC285	661.4645197	3279.118241	4.95736074	3.09E-70
SH2D5	17.87414745	87.3361069	4.8861691	5.50E-10
PDE2A	5.251034015	25.3668328	4.83082622	0.0009586
DHRS9	16.78267705	80.80383539	4.8147167	3.14E-09
PROCR	63.55298543	303.3707248	4.77350864	8.56E-28
GBP1P1	17.31637703	82.38970082	4.75790638	2.44E-09
LGALS9	119.1898756	552.4136405	4.63473628	5.77E-43
C11orf96	4.72764993	21.8883074	4.62984944	0.0029896
LOC64410(	14.70289524	67.85964364	4.61539326	8.94E-08
GREM2	6.297802184	28.97044254	4.60008773	0.0005834
AOX1	142.2806707	651.7476092	4.58071786	6.67E-48
DDX58	2259.677106	10167.18508	4.4993973	1.26E-147
ITK	8.394777157	37.30333566	4.4436362	0.0001062
DUSP5	154.9381705	683.4343239	4.41101326	8.71E-48
MMP24	9.465615761	41.73055758	4.4086469	4.45E-05
C12orf39	9.469054395	41.69987812	4.40380595	4.46E-05
SLC29A1	309.3735886	1358.958167	4.3926121	2.02E-73
4-Mar	33.57223136	146.4640245	4.36265385	3.87E-10
MICAL2	45.66508345	196.6808725	4.30702974	8.82E-18
S100A2	231.0317421	986.1348338	4.26839544	5.74E-59
PTX3	223.170665	952.241565	4.26687605	8.33E-58
RHOV	17.34732473	73.50931061	4.23750127	1.03E-07
NAV3	329.7133566	1356.3363	4.11368321	2.69E-68
IFI35	933.6647956	3802.441088	4.07259769	2.45E-105
SFN	20.47731334	82.75785426	4.04144103	3.49E-08
MMP7	14.17607252	57.08422693	4.02680128	4.46E-06
RPL13AP6	13.14305889	52.35257702	3.98328711	1.22E-05
RTP4	295.0565321	1174.08398	3.97918315	9.03E-61
ASPHD1	9.45529986	37.60776366	3.97742686	0.0002158
ADAMTS2(	10.5089453	41.69987812	3.96803646	0.0001073
IL15RA	28.36589958	112.4315577	3.96361686	2.66E-10
TNFSF13B	30.98282001	122.5650724	3.95590435	4.50E-11
PTGES	90.84460778	357.3752593	3.93391824	2.40E-26
MX1	2256.226613	8869.922439	3.93130831	1.38E-122
RND1	46.73248342	182.7950838	3.91152086	3.20E-15
SYTL5	380.6329127	1482.030591	3.89359549	1.72E-67

AFAP1L1	32.02958817	124.6394426	3.89138449	4.33E-11
CCDC86	306.7704227	1193.746887	3.89133632	8.19E-60
TRAF1	166.5076385	646.3645911	3.88189153	4.66E-38
SP110	736.7733711	2848.339909	3.86596479	1.35E-89
WNT10B	12.07222028	46.55424585	3.85631183	5.70E-05
HMOX1	897.3724848	3450.198678	3.84477877	3.76E-95
SLC25A19	190.6555177	724.1170469	3.79803877	1.17E-42
IL1B	7.361763521	27.90139475	3.79004225	0.0021109
DUSP4	436.8722755	1652.926951	3.78354738	6.97E-69
SLC37A2	28.34870641	106.5388216	3.75815461	2.13E-09
KMO	11.02545211	41.11933504	3.72949196	0.0001955
STX11	24.68501782	91.91435955	3.72348767	2.56E-08
S100A3	21.52408151	79.91886431	3.71299766	2.23E-07
IL11	825.548513	3064.138558	3.71163961	2.31E-87
VEGFC	89.79096234	332.652695	3.70474585	3.43E-23
ADCY8	10.5089453	38.83020872	3.69496725	0.0003251
ANXA13	7.34113172	26.83234695	3.65506954	0.003107
VDR	86.16166008	313.9951108	3.64425558	5.29E-22
RRP9	274.7030096	997.6040453	3.63157305	9.91E-50
RAET1E	12.07565892	43.50049973	3.60232928	0.0001716
LOC100505	25.72834736	92.64593336	3.60092827	1.09E-06
IER3	97.65891678	350.8382547	3.59248563	1.21E-23
10-Mar	33.6066177	120.641733	3.58982073	5.85E-10
TP63	17.84663838	63.86193407	3.57837329	5.67E-06
C17orf96	46.74967658	167.2242918	3.57701494	5.57E-13
VGLL2	11.03232938	39.4107518	3.57229651	0.0003939
ADRB2	12.61623617	44.96601388	3.56413857	0.0001461
INPP4B	12.599043	44.3854708	3.52292399	0.0001761
SLC16A6	24.14100193	84.8652704	3.51539968	2.53E-07
HERC5	1245.937984	4376.028028	3.51223583	1.28E-90
RAET1K	28.87896777	100.7688034	3.48934921	2.28E-08
BOP1	45.17264706	156.477188	3.46398093	6.30E-12
PNPT1	1319.948336	4557.053084	3.45244807	1.31E-89
CHRM4	12.61279754	43.53117918	3.45135003	0.0002472
RAB3IL1	14.69945661	50.64162724	3.44513601	0.0027498
CYB5R2	61.96564001	213.4103842	3.44401162	3.42E-15
KCNE4	182.2504246	627.6503811	3.44388982	4.94E-24
THSD1	49.88310383	171.1016501	3.4300522	1.25E-12
RHEBL1	36.23385402	123.6624331	3.41289759	1.11E-09
ISG20	60.41611956	205.9931416	3.40957253	1.35E-14
APOL3	324.5139021	1104.886273	3.4047425	1.40E-48
C3	21.01101333	71.52224575	3.404036	2.84E-06
BATF3	54.0701765	182.8564427	3.38183551	4.49E-13
CD70	714.9631712	2416.475963	3.37986075	3.38E-57
RAB39	29.39891322	99.2136174	3.37473759	5.99E-08
AEN	271.4870552	915.825567	3.37336735	4.99E-43
CREG2	28.8720905	96.73804782	3.35057303	8.49E-08

HERC6	1022.271444	3417.957452	3.34349303	1.38E-78
SNORD17	15.22284069	50.64399377	3.32684253	0.0001112
EXOSC5	189.6878381	629.2692925	3.31739398	1.71E-17
IFIH1	1479.097649	4885.389528	3.30295267	1.69E-85
C10orf47	11.03576802	36.41836459	3.30002992	0.0011372
C12orf34	36.24760855	119.4476009	3.29532363	6.10E-06
IFRD2	434.3825845	1428.410776	3.28837027	4.72E-51
SCO2	142.4147774	467.2982014	3.28124799	5.01E-18
KBTBD8	66.68297404	217.4694526	3.26124405	1.64E-14
SLC43A2	86.69192143	282.0960065	3.2540057	9.45E-18
CD82	14.69945661	47.46989638	3.22936403	0.0002517
DGAT2	11.55571347	37.30333566	3.22812916	0.0011455
KALRN	11.5591521	37.27265621	3.22451473	0.001148
ZNF488	24.67470192	79.39968014	3.21785772	2.17E-06
PGF	47.27306067	151.9296148	3.21387303	1.05E-10
SYP	19.41679064	62.36574046	3.21194896	2.67E-05
PTPRH	19.97112242	64.07669024	3.20846715	2.23E-05
C11orf9	20.46699744	65.47847895	3.19922251	1.69E-05
PNP	620.5415525	1975.358826	3.18328212	1.58E-59
C7orf40	140.2352752	446.1155214	3.18119332	1.91E-24
ATF3	363.9912197	1152.620452	3.16661609	2.68E-45
KRT14	20.49794514	64.80826405	3.16169565	2.27E-05
NFKBIZ	458.3654093	1446.195136	3.15511404	7.50E-51
SPSB1	155.9574296	487.9994763	3.12905565	1.66E-25
ALDH1A3	5650.023907	17676.97424	3.12865477	1.01E-95
GCH1	65.10250588	203.5812974	3.1270885	4.53E-13
ADAMTS4	168.0193339	524.4697336	3.12148442	2.81E-14
SLC19A1	92.452585	288.3828424	3.11925126	3.60E-17
CCRN4L	138.6410525	431.4013875	3.11164247	3.54E-23
BMP6	37.28749946	116.0021215	3.11101906	2.83E-08
DAB1	18.38033837	56.87183729	3.09416705	9.50E-05
IFI6	6798.613755	20878.3522	3.07097196	9.07E-63
HAS3	198.4856471	609.0447752	3.06845751	3.67E-17
GRPR	34.6671404	106.2037142	3.06352681	1.43E-07
GPR85	13.66644297	41.85090885	3.06231175	0.000822
HECW2	15.75997931	48.20147018	3.05847294	0.0003525
KRT16	152.3040569	462.6916358	3.03794689	1.27E-23
KCNMB2	40.4415585	122.5957519	3.03142996	2.15E-08
IFI44L	3552.84001	10769.1926	3.03115045	2.23E-86
TRMT61A	163.4395453	493.5500053	3.01977104	8.19E-13
LOC100506	12.08941345	36.44904404	3.01495554	0.0021896
FBLN2	12.599043	37.73048147	2.99471011	0.0018626
SPP1	2583.684817	7729.137071	2.99151701	1.60E-80
TAF4B	105.5234326	315.3709532	2.98863433	2.42E-17
TEX15	32.54609499	97.10383472	2.98357867	6.64E-07
C12orf45	143.4099661	427.0119447	2.97756116	4.16E-14
TMEM79	87.69054873	258.409444	2.94683347	1.40E-14

GADD45A	566.0511509	1667.475855	2.9458042	2.47E-49
BIRC3	283.9898164	835.8760232	2.94333097	9.94E-34
PLA2G4A	232.6087716	684.5883104	2.94308897	1.15E-29
PADI3	223.6837331	653.6379026	2.92215215	2.02E-28
TRIM21	558.7168965	1631.097636	2.91936336	4.66E-48
IL7	66.09769455	192.5911246	2.91373437	6.41E-10
YDJC	161.2909908	468.7967615	2.90652788	1.88E-22
EMILIN2	184.2923814	535.3159754	2.90471028	1.37E-24
SERTAD1	212.8027036	617.3658357	2.90111838	2.24E-17
CCDC19	26.77167689	77.66041745	2.90084247	1.35E-05
KCNQ5	284.5303937	823.0899618	2.89280154	1.66E-32
C21orf70	21.03164513	60.74682904	2.88835365	0.0002054
IFI44	3934.781027	11337.0353	2.88123665	6.14E-80
HES4	19.45117697	55.95382023	2.87662902	0.0009623
NLE1	277.3302459	796.5596763	2.87224235	1.42E-31
CD3EAP	385.993983	1108.386012	2.87151111	1.98E-38
PCDH1	131.8267435	378.50368	2.87122074	8.67E-19
SERPINB5	14.69945661	42.09634448	2.86380277	0.0014871
PLAUR	1175.756376	3362.161677	2.85957342	1.16E-61
C5orf56	43.59217891	124.6394426	2.85921571	5.90E-08
LOC541471	126.0626413	360.3983259	2.85888287	4.65E-18
C1QL4	14.19326569	40.56947142	2.8583606	0.0017785
XAF1	1833.630842	5234.83546	2.85490151	5.26E-69
IRF1	693.697699	1980.003085	2.85427368	1.01E-50
TNFRSF12A	2545.910335	7216.393731	2.83450428	8.71E-73
STMN3	25.20496327	71.40189447	2.83285057	3.96E-05
CHGB	140.6933253	398.2491587	2.83061871	3.40E-19
F12	13.13962025	37.11925894	2.82498719	0.002973
MGP	48.79507205	137.6449932	2.82087898	2.06E-08
SEMA7A	202.7311766	569.6221908	2.80974145	1.49E-24
KHDRBS3	1058.994296	2975.056088	2.80932211	1.54E-57
TNFAIP3	332.9086793	934.2967079	2.80646545	1.45E-33
MAFF	234.6988694	658.0674911	2.80388011	8.30E-27
DGKH	30.44912002	85.35377512	2.80316065	9.19E-06
GBP1	980.7384155	2740.91375	2.79474497	1.73E-55
HPD	14.18638842	39.56178253	2.78871418	0.0024329
SPRY1	175.3639043	489.0047986	2.78851455	8.93E-22
LY6K	38.33770626	106.8715625	2.7876358	7.12E-07
CITED4	16.79987021	46.79731494	2.78557598	0.0010179
SGK1	689.3249401	1915.310525	2.77853072	1.23E-47
MET	3532.283608	9812.589819	2.77797338	1.43E-73
NT5C3	835.2589835	2314.295991	2.77075259	3.22E-51
EBF1	22.55365651	62.45777882	2.76929724	0.0001607
PLSCR1	3806.34217	10488.15372	2.75544164	3.37E-73
CMTM7	121.3349914	333.9293994	2.75212777	3.41E-16
NR4A2	59.34184232	163.316254	2.75212645	2.37E-09
HOMER3	386.5792625	1063.427098	2.75086431	4.93E-35

LHX1	14.16575662	38.95292654	2.74979499	0.0032309
IPCEF1	18.90372245	51.95611066	2.74845924	0.0006008
ACOX2	74.0584921	203.3382284	2.74564365	5.07E-11
LOC654433	26.27236324	71.9517581	2.73868618	5.90E-05
C14orf80	43.11005843	118.0434456	2.73818802	0.0013094
LOC100287	108.6431053	296.0148412	2.72465372	1.44E-14
NR4A3	21.00757469	57.2376242	2.72461838	0.0003537
DOHH	67.24074446	183.1301913	2.72350035	4.87E-10
PINX1	117.1307256	318.6064095	2.72009251	2.40E-15
SSTR2	97.67267131	264.7576388	2.71066241	2.91E-13
FXYD5	339.7023565	920.2834683	2.70908768	1.80E-31
IFI30	359.1707266	972.8508015	2.7086027	1.67E-32
MYH15	17.85007702	48.23214964	2.70206955	0.0011294
MAK	14.68914071	39.59246198	2.69535589	0.0032145
ULBP2	202.6624039	544.9916648	2.68916017	3.33E-22
LOC401010	80.33222385	214.1443245	2.6657338	5.28E-11
C19orf48	814.2025562	2170.28387	2.66553311	6.29E-47
RGS17	72.99109212	193.5091416	2.65113367	4.38E-10
FOXQ1	21.02820649	55.74143059	2.65079338	0.0005479
SMOX	379.1762354	1004.11037	2.64813635	6.72E-32
AGTRAP	380.3536716	1006.621352	2.64654038	7.51E-22
NKX3-1	240.4320238	635.9030686	2.64483515	6.08E-24
ICOSLG	42.54884937	112.5212295	2.64451874	1.22E-06
MRTO4	1301.650524	3442.149	2.64444944	3.43E-54
METTL12	21.53095878	56.68776057	2.63284887	0.0005352
C6orf191	30.9415564	81.32301956	2.62827825	3.74E-05
SLC30A3	40.44499713	106.1093093	2.62354597	2.61E-06
FAM86EP	29.40235185	77.04919491	2.62051129	6.04E-05
S1PR1	448.886039	1174.565385	2.61662267	3.90E-34
SMTN	411.8564371	1077.555956	2.61633875	1.48E-32
SAMHD1	1653.773115	4324.222948	2.61476191	1.87E-56
P2RX7	97.09770772	253.7698325	2.61355122	3.95E-12
PKMYT1	210.70229	550.2070864	2.61130093	3.31E-12
SAMD9	4358.641624	11361.35438	2.60662733	1.32E-66
SLC25A28	472.5793068	1231.604819	2.60613362	1.34E-34
SNHG1	724.0711691	1885.641555	2.60422129	2.52E-42
STX1A	224.761449	585.0112726	2.60280967	3.88E-22
DHX58	685.5161171	1782.847907	2.60073813	2.48E-41
EMR1	231.0833216	600.6103776	2.59910743	1.76E-22
PDCD2L	245.8068487	638.7113791	2.59842792	2.36E-23
COL6A3	88.76138734	230.5977211	2.59795084	3.14E-11
RNF122	61.95532411	160.3545463	2.58822867	1.76E-08
CORO1A	38.37896986	99.33160214	2.58817791	0.0008764
IL20RB	41.48832667	107.1783571	2.58333767	3.24E-06
TMEM158	102.9580917	265.7346483	2.58099819	2.26E-12
KLF4	108.1334757	278.7095195	2.57745825	9.18E-13
WDR4	125.0055573	322.1486603	2.57707471	3.06E-14

TMOD1	17.86383155	45.94302332	2.57184598	0.0023171
IMP4	435.9217891	1120.629309	2.57071185	2.94E-32
C9orf167	84.02686014	215.6711976	2.56669352	1.84E-10
HLX	21.02820649	53.87945008	2.56224658	0.0009646
PYCRL	163.3879658	418.5516007	2.56170397	4.36E-17
METTL1	178.5523497	457.3133509	2.56122841	3.22E-15
S100A16	3571.301452	9132.948	2.55731646	2.08E-62
HK2	376.5524377	962.1957361	2.55527687	2.55E-29
CD55	954.9894363	2439.7118	2.5547003	2.01E-45
CCDC85B	62.02753541	158.4005274	2.55371306	2.99E-05
ARID5A	107.1589189	273.4563189	2.55187643	1.89E-12
PKIB	210.599131	536.2009465	2.54607388	4.15E-20
ADAM11	27.31569278	69.50686797	2.54457643	0.0022872
CAV1	4459.946324	11336.75453	2.54190381	2.02E-63
PSMB9	632.3723566	1605.881834	2.53945609	8.39E-38
PDXP	576.5291485	1461.980684	2.53583134	4.85E-36
GRWD1	865.0602169	2192.908485	2.53497785	5.12E-43
GATA3	155.9539909	394.7092744	2.5309341	4.56E-16
EHD4	1178.858856	2980.63493	2.52840696	8.48E-48
PIK3AP1	259.9072713	656.6845491	2.52661092	8.97E-23
RCL1	318.7669931	804.8642565	2.52492973	8.34E-26
CEP72	65.10938315	164.2908969	2.523306	7.60E-07
CSRNP1	131.2655345	330.8756532	2.52065902	4.46E-14
ITPKA	37.31844716	94.0500886	2.5202037	0.0016873
MLKL	295.0427776	742.7769976	2.51752306	2.28E-24
KCNN4	1018.834706	2562.850316	2.51547214	5.45E-45
FRMD4B	25.18433147	63.28139099	2.51272864	0.0004431
WT1	47.29025384	118.4422785	2.50458115	1.97E-06
CDC42EP2	59.31777188	148.4817688	2.50315823	1.39E-07
PARP12	1246.73302	3120.401539	2.50286267	1.75E-47
TAP1	3089.442928	7722.418356	2.49961515	3.05E-58
NOP16	316.2291612	789.7890688	2.49752131	5.58E-25
LYAR	703.1289285	1755.038551	2.49604088	4.51E-38
CHTF18	148.1685637	368.964822	2.49016939	0.0009627
TOMM40	1168.235724	2900.718433	2.4829907	2.00E-34
CRABP2	108.674053	268.6680431	2.47223726	1.33E-11
MYB	100.2689599	247.6009813	2.4693682	5.93E-11
ATAD3B	67.22011266	165.7587776	2.46591044	4.38E-08
DDX60	1355.675999	3341.708204	2.46497556	4.07E-47
MAN1A1	771.3304752	1901.162735	2.46478364	1.57E-38
TAP2	2462.728076	6059.112369	2.46032537	3.34E-54
IFITM1	5511.587749	13555.53027	2.45946012	2.00E-60
GPR3	113.3741939	278.8275042	2.459356	6.34E-12
BMP4	36.76411537	90.2057763	2.45363653	4.22E-05
NRGN	71.43813304	175.2527569	2.4532102	2.29E-08
PITPNC1	40.95118805	100.0962219	2.44428127	1.79E-05
ADAMTS6	39.91129715	97.41062926	2.44067811	2.42E-05

IL12A	46.1850289	112.3701988	2.43304381	7.11E-06
AP1S3	502.9527769	1218.651161	2.42299321	2.82E-30
LANCL3	54.57980605	131.6932648	2.41285696	1.54E-06
RGS2	371.6838038	896.165027	2.41109518	2.27E-25
UCN2	32.03990408	77.2309051	2.41045994	0.0001778
HIST1H1C	311.9286137	751.7494262	2.4100047	9.15E-23
GUCA1B	28.35214505	68.19711762	2.40536007	0.0004742
PCOLCE2	76.14171253	183.0381529	2.40391432	2.36E-08
TONSL	284.7504662	684.0337137	2.40222158	1.10E-11
ARMC6	453.8681478	1089.761474	2.40105299	1.84E-18
CDT1	300.0523955	719.1092819	2.39661237	2.01E-08
RPF2	553.910158	1326.572925	2.39492435	8.05E-31
CYP2J2	260.9162145	624.3016732	2.39272854	5.91E-20
CD320	483.3358337	1153.415751	2.38636507	6.12E-20
NFIL3	164.8618363	393.057317	2.38416195	1.53E-14
PTPRR	34.63963133	82.57377754	2.38379493	0.000133
MSX1	78.2077398	186.2429298	2.38138745	2.44E-08
C14orf49	25.20152464	59.95389633	2.37897894	0.0011263
GLDC	128.6348595	305.7235766	2.3766775	4.98E-12
PVT1	225.2607627	534.2752405	2.37180783	8.93E-18
C3orf26	884.1365828	2094.155059	2.36858773	4.73E-37
UPP1	1305.69934	3090.199185	2.36670043	1.84E-42
NCRNA001	108.281337	255.9975999	2.3641895	1.46E-05
FAM46A	612.7354935	1448.581034	2.3641213	1.92E-31
FGFR4	39.41542213	93.16748406	2.36373173	5.99E-05
CNKSR2	148.0894751	349.0116867	2.35676226	4.91E-13
PFAS	648.5594383	1526.177726	2.35318097	5.85E-32
GNG4	75.60457391	177.9100366	2.35316499	6.78E-08
EIF5AL1	268.3845756	630.4728908	2.34913981	3.03E-16
TBL3	235.4216942	552.6189305	2.34735772	5.99E-08
IL22RA1	70.90443305	166.3393207	2.34596503	1.70E-07
MCTP1	358.5407449	839.5480915	2.34156955	5.31E-23
C19orf76	37.32188579	87.36678636	2.34089957	0.0001125
KCNJ2	351.819279	822.8752056	2.33891448	9.34E-23
PML	863.0835941	2013.792483	2.33325311	4.51E-15
C10orf2	516.1233449	1200.519861	2.32603286	9.15E-28
FOXL1	59.34871959	137.9187418	2.32387055	1.97E-06
EIF2AK2	2362.229439	5485.414629	2.32213456	4.64E-47
DHX37	655.3565541	1520.587051	2.32024391	4.67E-31
FAM86A	132.3501276	306.3985246	2.31506029	1.81E-11
ETS2	1309.456583	3030.429366	2.31426487	2.95E-40
NR2C2AP	307.2972455	710.9958781	2.31370729	2.10E-20
OSR2	97.66579404	225.8637047	2.31261832	3.15E-09
LAP3	2032.071075	4697.681084	2.31177007	4.26E-45
CTU1	42.02202665	97.04247582	2.30932403	5.88E-05
RFFL	180.6733951	416.8736969	2.30733306	3.18E-14
PPARGC1B	32.04334271	73.87273098	2.30540027	0.0004333

HAUS7	135.5316957	312.317207	2.304385	1.35E-11
RPL41	116.1011506	267.3228802	2.30249984	2.62E-10
STYK1	28.35214505	65.26608932	2.30198065	0.0010263
CFH	115.5777665	265.8880455	2.30051206	2.97E-10
VEPH1	137.0605844	315.1609301	2.29942789	1.52E-11
MPP7	51.47044925	118.2912478	2.29823616	1.25E-05
RPL17	48.33014474	111.0533488	2.29780708	0.0001145
MPV17L2	262.1314757	601.8941816	2.29615379	4.31E-18
PMAIP1	1794.59367	4109.290338	2.28981658	7.00E-43
TDRKH	134.3852072	307.5525111	2.28858903	2.37E-11
SYTL2	104.4697871	239.0533321	2.28825327	2.07E-09
TGM5	54.58324469	124.8848782	2.28797095	8.21E-06
NLRC5	696.8792671	1594.063316	2.28743111	7.68E-31
FAIM3	23.10111103	52.84108173	2.2873827	0.0032327
C11orf82	432.0827302	987.5083096	2.28546119	4.06E-24
PIM3	493.8379018	1127.952147	2.28405342	8.01E-15
POLR3G	375.3474924	855.9613425	2.28045041	3.22E-22
DPH2	932.7349409	2125.575125	2.27886298	2.13E-34
C16orf91	78.78614203	179.0711228	2.27287589	1.76E-07
PGBD5	68.80058081	156.2647983	2.2712715	8.35E-07
TMEM159	45.69946978	103.79187	2.27118324	5.12E-05
MYC	1342.229628	3041.749913	2.2661919	1.38E-38
XRCC3	390.711317	884.8114337	2.26461685	2.51E-22
THBS2	685.7781651	1552.403584	2.26371101	9.61E-30
NOL12	268.9457847	608.1786509	2.26134294	9.73E-18
DDX39A	1186.946883	2683.822509	2.26111425	9.51E-37
PLS1	693.5532764	1567.481138	2.26007315	8.70E-30
MIIP	170.677518	385.5504026	2.25894076	5.43E-13
CLDN1	220.5503059	498.1377241	2.25861271	2.05E-15
C19orf24	178.1596337	401.5436074	2.25384168	6.21E-06
AMPD3	64.10043995	144.4486467	2.25347356	0.0004555
MICB	555.5731533	1250.370922	2.25059637	1.61E-26
DDX60L	1060.109837	2379.517201	2.24459496	8.93E-35
IGFLR1	50.98489013	114.2628588	2.24111219	0.0006488
RAB20	99.266894	222.2600949	2.2390153	2.57E-06
NRARP	61.46632636	137.5529549	2.23785873	4.88E-06
NOC4L	145.0076274	324.4330535	2.23735164	6.10E-08
PTHLH	692.0725286	1548.132125	2.2369507	5.88E-29
SOCS2	148.0447729	331.0031041	2.23583108	2.23E-11
TRAF2	452.8660819	1011.617285	2.23381111	2.02E-14
DDX10	542.9190922	1211.36137	2.23120054	1.34E-25
IPO4	826.6778084	1843.526278	2.23004205	3.86E-31
C11orf83	101.3879394	225.8637047	2.22771768	1.17E-08
TBRG4	688.4260332	1533.507663	2.22755618	1.34E-28
OAF	85.08738284	189.5090655	2.22722875	1.42E-07
ANKRD37	30.97938137	68.99005034	2.22696669	0.0010898
DBF4B	531.4630991	1183.233386	2.22636979	2.59E-25

PDCD1LG2	285.09848	633.7342935	2.222861	1.57E-17
STAT1	11930.86792	26520.52689	2.22284976	9.02E-51
ETV5	3181.255927	7069.160439	2.22212881	1.21E-44
RRP7A	505.2767017	1122.670634	2.22189274	1.59E-24
TLR3	262.5551394	582.8118181	2.21976922	1.26E-16
TRAIP	188.0454745	416.9255896	2.21715301	3.98E-07
CCL26	164.8893453	365.3730449	2.21586813	4.68E-12
TIPIN	360.0799495	797.4824264	2.21473711	1.97E-17
NOP2	1556.878287	3447.397467	2.21430121	5.57E-38
GRB10	4069.98878	8994.897075	2.21005451	1.06E-45
RPUSD1	176.0489042	388.9085767	2.20909399	4.21E-07
NCRNA001	198.0447903	437.1430929	2.20729408	1.55E-13
SH3RF2	53.0199697	116.9484514	2.20574346	3.19E-05
ARG2	254.1397304	559.9771807	2.20342242	5.67E-16
MRM1	71.45532621	157.395205	2.20270781	1.73E-06
BCL2L12	346.1961608	762.3431327	2.20205542	6.64E-18
IKZF2	91.88449868	202.2385011	2.20100783	8.91E-08
UBE2L6	1870.852296	4116.457498	2.20031133	3.48E-39
SLC15A3	268.343312	590.3210991	2.1998726	1.80E-16
SNORA67	29.93949047	65.81595294	2.19829903	0.0016848
RNASEH2A	1087.466793	2390.54752	2.19827174	3.20E-33
EFNB1	63.5495468	139.6910505	2.19814393	6.64E-06
IQGAP2	194.2573109	426.9765321	2.19799466	3.27E-13
MYBBP1A	1190.114696	2614.544425	2.19688441	7.43E-14
WDR74	367.5792583	807.335093	2.1963565	6.79E-20
PSMB10	225.463642	494.4373429	2.19298038	6.93E-06
STRA13	630.271943	1381.32078	2.19162664	2.00E-26
SDF2L1	128.1871254	280.6894847	2.18968546	1.28E-09
KCNJ14	53.55710832	116.9791309	2.1841943	3.85E-05
CCND1	2398.314008	5230.7103	2.18099477	1.56E-40
PGAM5	1009.324388	2200.915736	2.18058313	1.13E-31
VAT1L	705.6839535	1538.458802	2.18009605	2.73E-27
DCTPP1	1016.744608	2213.144835	2.1766969	1.07E-31
TMCC3	157.9890705	343.6121884	2.17491113	6.09E-08
RASGRP3	74.05161483	160.9988148	2.1741432	2.02E-06
RRP1	315.6817067	686.1741758	2.17362667	1.11E-17
MRPS12	464.9795658	1010.541137	2.17330225	3.00E-07
LOC100125	38.88172215	84.4664375	2.17239445	0.000429
C9orf142	207.548231	450.0849181	2.16857988	1.72E-09
MON1A	124.5131209	270.0061064	2.16849521	2.28E-09
BYSL	569.7457872	1234.339938	2.16647488	2.36E-24
RGS16	39.88722671	86.26705911	2.16277406	0.0003994
EIF5A	5230.462435	11312.05581	2.16272575	1.73E-25
RPL22L1	565.4589942	1222.889573	2.16264943	3.63E-24
SP100	2543.312032	5495.309808	2.16069037	4.72E-40
GPATCH4	1107.923475	2392.829546	2.15974262	5.49E-32
C12orf24	398.9994966	861.5520171	2.15928096	6.55E-20

RHBDF2	189.6568904	408.9938959	2.15649374	2.08E-12
EPHA2	5748.496357	12385.91104	2.15463493	5.54E-43
LOC388796	248.4168918	535.157845	2.15427317	7.60E-15
DHODH	186.9987063	402.5867088	2.15288499	3.50E-12
COL19A1	83.48972152	179.5265815	2.15028363	7.60E-07
FAM46C	83.44845792	179.2221535	2.14769881	8.39E-07
SORD	259.4389053	557.0508855	2.14713705	4.60E-15
GPRIN1	215.8192173	463.3004918	2.14670638	2.28E-13
NEGR1	174.8130111	375.2634906	2.14665652	1.51E-11
SPRY4	610.6763435	1310.600933	2.14614656	1.61E-24
ORC1	656.7307043	1409.203328	2.14578566	2.40E-25
IFI16	5154.302938	11059.49633	2.14568225	9.75E-44
CTSC	1449.805334	3106.784766	2.14289787	3.37E-34
LOC728554	140.7277116	301.3931261	2.14167574	8.41E-10
CDR2L	410.2622144	877.2101144	2.1381694	9.54E-20
ZNF239	134.3886458	287.2878482	2.13773899	1.84E-09
EXOSC2	899.3525462	1921.012085	2.13599449	1.05E-28
DNLZ	62.49590136	133.3074431	2.13305897	1.75E-05
PLEKHA4	629.879227	1343.321283	2.13266484	1.54E-16
RIN1	95.10045312	202.4815702	2.1291336	2.33E-07
IL6R	38.86109034	82.6658159	2.12721298	0.0006779
CHAC2	214.2318719	455.5481418	2.12642562	6.01E-13
WRAP53	394.8846352	839.446587	2.1258021	5.02E-19
SAAL1	425.2959302	904.0188815	2.12562317	9.62E-20
PFKFB4	643.9177542	1366.979533	2.12291014	1.80E-24
TMEM51	181.1967792	384.5403471	2.12222507	1.54E-11
UBE2S	179.7022769	381.1869061	2.12121356	2.49E-08
LOC439990	76.66509662	162.4006035	2.11831212	3.28E-06
AVPI1	80.86936246	171.2833602	2.11802536	1.85E-06
TEAD4	102.3865667	216.648207	2.11598273	1.36E-07
ORC6	662.7389495	1401.760139	2.11510149	1.85E-24
FRMPD4	50.9401879	107.57719	2.1118334	0.0001397
C9orf140	628.8255816	1327.750491	2.11147658	3.16E-15
NPM3	307.7552955	649.7558112	2.11127419	4.37E-16
PNO1	799.0904635	1686.567685	2.1106092	1.78E-26
DCPS	519.8558061	1097.166969	2.11052172	1.56E-21
YRDC	710.9624966	1500.26103	2.11018308	3.64E-25
JMJD4	185.9828859	392.2926972	2.1092946	5.67E-07
PAX8	47.78269022	100.7074445	2.10761353	0.0002211
GNB1L	76.20704657	160.4465846	2.10540353	0.0001147
HMBS	482.7471156	1014.70881	2.10194691	7.84E-19
C15orf39	163.3982817	343.210989	2.10045654	2.09E-10
MAK16	713.4590648	1498.401416	2.10019255	7.53E-25
NMI	749.6791643	1574.386296	2.1000801	2.28E-25
FAM110A	35.73454037	74.94414531	2.09724666	0.0015105
TCEAL7	63.51172183	133.1564124	2.09656436	2.75E-05
C6orf138	36.75036084	76.98783601	2.09488653	0.0012575

SDCCAG3	1050.244628	2195.445413	2.09041337	9.23E-29
STEAP1	98.72287811	206.1158594	2.08782263	3.73E-07
ADPRHL2	814.5987108	1699.844617	2.08672638	6.50E-26
RPS2	3300.588802	6883.816766	2.08563295	3.04E-14
MCOLN1	264.2112575	551.0330651	2.08557754	3.94E-14
CLPB	203.2132971	423.7693888	2.08534281	7.42E-12
HTR7	501.9610269	1045.564813	2.08296014	1.70E-20
RNF19B	1483.277845	3088.601573	2.0822812	7.72E-32
LGI2	49.88998109	103.8815419	2.08221249	0.0002121
MTFP1	309.9623068	644.5073437	2.07930877	7.39E-13
EFNA3	43.59217891	90.63528865	2.07916399	0.0005805
HMGA2	510.8860654	1062.025309	2.07879091	1.66E-20
C19orf66	1138.647686	2366.787766	2.07859533	3.49E-29
RELT	191.7091631	398.1901663	2.07705339	2.98E-11
RBM28	579.669453	1202.8373	2.07504	7.82E-22
TRIB1	369.6487242	766.3384758	2.07315331	4.73E-17
UBASH3B	1065.667621	2204.800194	2.06893796	1.36E-19
ASPHD2	141.753848	293.2702561	2.06886981	5.14E-09
RAB27B	143.8611388	296.9045453	2.0638273	5.00E-09
LRRC2	163.8219454	337.3182529	2.05905413	7.08E-10
TNFRSF21	67.71598768	139.3535765	2.05791248	2.80E-05
RRP12	719.1261735	1479.245861	2.05700462	6.31E-12
FERMT1	81.912692	168.4750497	2.05676368	4.79E-06
TRAP1	1271.383652	2614.853672	2.05669915	2.23E-29
OCIAD2	2608.40766	5363.66607	2.056299	2.16E-35
IL1R1	197.4148085	405.850478	2.05582591	3.58E-11
KIAA0664	1096.845732	2254.139085	2.05511041	5.34E-17
IFI27	4124.528626	8464.966398	2.05234759	1.46E-23
NUDT1	321.3907908	659.3725083	2.05162228	3.05E-15
SNRPB	2269.051894	4652.254878	2.05030784	4.09E-34
SLITRK6	312.8962933	641.2435745	2.04938054	6.80E-15
C1orf135	319.7346727	655.0727373	2.04880106	4.48E-15
HRH1	378.0400627	774.1828642	2.04788577	1.06E-16
WDR12	825.7864905	1690.99254	2.04773578	1.06E-24
TRIM22	2887.290865	5904.046436	2.04483951	1.55E-35
PUS1	318.3123817	650.5794234	2.04383951	5.14E-15
BLOC1S3	164.3831544	335.6733951	2.04201821	1.24E-09
LOC43994[illegible]	77.19191934	157.362159	2.03858332	1.09E-05
CIRH1A	1850.963701	3773.289021	2.03855377	6.52E-32
IGFBP6	329.8405861	672.378059	2.03849401	3.38E-15
NOP56	2853.332795	5811.785222	2.036841	5.00E-35
ECE2	226.4416375	460.6762581	2.03441497	5.09E-10
PWP2	464.2773728	943.3942208	2.03196252	2.53E-18
CDCA5	1144.818258	2324.724467	2.03064936	2.19E-27
POC1A	407.50431	827.333107	2.03024382	5.54E-17
PDCD11	1184.612642	2403.845666	2.02922507	1.13E-27
ARL4D	74.068808	150.251711	2.02854231	1.99E-05

FAM83G	255.8680598	518.6785021	2.02713267	2.05E-09
C20orf27	472.2656794	957.0982993	2.0266099	9.82E-15
TIMM13	489.127445	990.8617506	2.02577418	1.70E-10
TCOF1	1360.723442	2753.947614	2.02388489	8.50E-29
NXT1	268.3639438	542.8889818	2.02295798	5.10E-13
CLN6	829.2981674	1677.590523	2.02290393	6.14E-24
PHB	2197.06358	4441.223292	2.02143594	1.47E-32
PES1	1798.797224	3635.039819	2.02081689	4.87E-31
TRANK1	940.3725069	1900.31081	2.02080643	4.11E-25
GEMIN4	1037.772814	2094.867786	2.01861887	6.05E-26
MRPL55	206.0124651	415.8589084	2.01861042	0.0005219
IL1A	36.20634495	72.95944699	2.01510114	0.0028343
PAK1IP1	772.3050321	1555.388871	2.01395667	6.21E-23
CDK18	100.3377326	201.8420347	2.01162643	1.44E-06
HSPBP1	748.7107729	1505.818658	2.01121543	1.41E-08
FAM84B	2760.821753	5543.626896	2.00796263	1.67E-33
GBP2	462.6040615	928.4346513	2.00697471	1.18E-17
AOC2	57.76137416	115.8204113	2.00515332	0.000216
NOL6	1148.392542	2302.519899	2.00499378	1.65E-26
ADM	366.0744401	733.9272869	2.00485805	2.44E-15
MRPL4	688.194933	1379.307769	2.00423994	1.79E-11
SLCO5A1	166.9656885	334.3541786	2.00253227	2.66E-09
TSEN54	234.7848352	469.9885272	2.001784	1.28E-11
KCNJ8	163.2848067	326.6962335	2.00077545	4.43E-09
FHL2	1939.800738	3878.157038	1.99925537	1.51E-30
POLR1E	977.6428132	1950.348314	1.99494978	1.52E-24
PLAU	7593.701529	15135.49068	1.99316376	2.81E-37
WDR54	546.1935034	1088.640533	1.99314076	7.09E-19
WDR43	1650.577792	3289.471245	1.99292106	3.58E-29
PARP10	1102.912433	2196.851849	1.99186425	4.88E-05
ORAI1	200.7029743	399.6533139	1.99126752	1.83E-08
ICAM3	118.7352641	236.4267317	1.99120904	3.20E-07
BAIAP2L1	82.9835306	164.9682114	1.98796328	1.56E-05
NFKBIE	152.293741	302.5825252	1.98683494	1.85E-08
C19orf70	353.0998743	701.4947992	1.9866753	1.66E-05
SNAP25	63.00209228	125.1586267	1.98657889	0.0001338
AXL	6255.592928	12423.31833	1.98595377	2.43E-36
IL27RA	147.0461456	291.2855578	1.98091257	3.25E-08
FLT3LG	63.04679451	124.8541987	1.98034174	0.0001469
TIMP1	1427.365152	2825.578463	1.97957647	2.31E-27
C9orf150	249.9767282	494.4727554	1.97807516	1.15E-11
SLC5A6	810.3806904	1602.792676	1.97782683	2.88E-22
DHX34	430.70858	851.1140743	1.97607875	3.35E-16
ITPRIP	540.3193649	1066.964616	1.97469253	2.82E-18
SNHG5	220.0956945	434.2734235	1.97311185	1.14E-10
CKS2	1319.517795	2601.418609	1.97149187	2.20E-26
EXOSC8	553.0800237	1090.358583	1.97143006	4.60E-14

ATP6V1C2	61.48351953	121.0688788	1.96912733	0.0002022
OSGIN1	102.4347076	201.6886375	1.96894824	2.67E-06
SSSCA1	103.5158621	203.7960536	1.96874227	4.07E-06
KCNK2	77.67747845	152.9113573	1.96854172	0.0022554
TMEM161/	252.6039645	496.9671718	1.96737677	1.71E-10
RNMTL1	334.007027	656.9016718	1.96673009	8.56E-14
THOC3	100.8129758	198.1180737	1.9652041	3.55E-06
LRAT	679.9246584	1335.663338	1.96442844	4.19E-20
ATOX1	823.985238	1617.518642	1.96304323	7.59E-22
AQP1	84.54680559	165.9098083	1.96234272	1.74E-05
UCK2	851.0877355	1667.492421	1.95924856	5.40E-22
ATAD3A	267.4306506	523.8656107	1.95888396	7.70E-08
EAF2	49.91405153	97.6843778	1.95705167	0.0008359
SLC45A3	91.89137595	179.8333761	1.95702126	1.03E-05
GRPEL1	993.8333335	1944.847311	1.95691495	2.20E-23
SAMD9L	3166.849466	6194.959193	1.95618998	7.77E-32
PRKCQ	51.47044925	100.676765	1.956011	0.0007331
IFIT5	1962.957579	3832.405106	1.95236267	1.09E-28
SOD3	76.68916705	149.6381219	1.95122894	0.0007655
ABHD6	226.221565	441.3248791	1.95085238	1.71E-10
TIMM10	468.440375	913.6025326	1.95030698	2.62E-16
DKK1	14508.61042	28277.07803	1.94898596	1.86E-36
PA2G4P4	617.9487025	1202.598964	1.94611455	1.14E-18
PRMT5	2899.397472	5636.405014	1.94399184	8.18E-31
BRIX1	1266.480632	2461.567062	1.94362788	5.12E-25
TROAP	1175.728867	2284.202155	1.94279669	2.12E-24
ZNF593	233.8584192	453.8985509	1.94091174	0.0001388
CDCA4	1009.892474	1959.441094	1.94024725	6.07E-23
ZNF787	445.0153206	862.802775	1.93881589	7.06E-08
IRAK1	2895.997846	5609.426284	1.93695803	1.41E-30
SMYD5	813.4109587	1573.498958	1.93444524	8.58E-21
MYO19	1727.962275	3340.88924	1.93342719	5.96E-14
RRS1	556.1206078	1074.867996	1.93279656	1.99E-17
TBC1D8	404.274601	781.2366864	1.93244068	1.25E-14
KIF11	5178.288489	10002.36089	1.93159591	6.79E-33
FBXL6	189.6672063	366.1069853	1.93025981	1.07E-07
EBNA1BP2	2466.171692	4751.050816	1.92648826	4.85E-29
REEP4	445.8557708	858.8616913	1.9263218	2.35E-15
HR	68.80745808	132.4861975	1.92546275	0.0001705
DCAF15	393.4623442	757.5147114	1.92525339	6.14E-12
TOE1	558.159126	1073.770636	1.92377153	3.39E-17
RRP7B	73.53854664	141.4303132	1.92321333	0.0001085
POLR3K	485.3468429	933.4141033	1.92318981	6.02E-16
EMG1	691.1014104	1329.046128	1.92308409	5.81E-19
AGTR1	50.42024244	96.95280399	1.92289444	0.0011932
DDX21	5627.129826	10814.22225	1.92180074	9.54E-33
IER5	426.8867143	819.8851849	1.92061537	8.60E-15

DNPEP	917.4845912	1760.987913	1.91936511	2.16E-21
ZNFX1	3715.666767	7130.520858	1.91904207	7.21E-31
UNC5D	115.5021165	221.5922466	1.91851243	2.32E-06
METTL11A	574.1151075	1101.060808	1.91783981	2.67E-17
NME1	1538.680196	2950.760755	1.91772193	4.87E-16
ALKBH2	358.1170812	686.11755	1.91590289	2.86E-13
ITGA5	1605.225625	3073.110986	1.91444177	1.06E-25
SIX1	292.9698731	560.8597853	1.91439406	8.54E-12
IMPDH2	4119.834059	7886.079098	1.91417397	3.44E-31
NOC2L	1176.50671	2251.57621	1.91378102	2.62E-23
TTPA	43.58186301	83.36907679	1.91293054	0.0028481
PGAM1	467.9341841	895.0440864	1.91275636	2.36E-15
ATP10A	360.3206538	688.6214325	1.91113505	3.20E-13
EPAS1	899.9618961	1719.318714	1.91043501	6.43E-21
LOC100506	97.66923268	186.5804038	1.91032937	1.43E-05
GPR153	76.72011475	146.5560629	1.91026908	0.0012129
RNF31	807.267895	1541.196288	1.90915097	5.78E-20
WDR18	271.1321641	517.1468959	1.90736093	5.13E-05
C1QTNF2	55.16508554	105.1960253	1.90693125	0.0008893
AARS2	418.5607098	797.7207624	1.90586633	2.57E-14
TTLL12	1226.527359	2337.050337	1.90542047	4.44E-17
SRPX2	193.7958222	369.2197238	1.9051996	7.36E-09
KIAA0754	796.952225	1515.57692	1.90171615	1.20E-19
MICA	964.4756839	1833.090702	1.90060852	3.63E-21
ALG3	1581.287502	3004.732158	1.9001808	5.01E-25
GMPR	588.721721	1118.526626	1.89992417	5.63E-17
CDC20	4121.98949	7825.606103	1.8985022	2.23E-30
PDRG1	398.593026	756.3890378	1.89764745	9.97E-14
NIP7	1317.871992	2500.399637	1.89730084	1.48E-23
HSPA14	358.5785699	680.3144858	1.89725361	6.49E-13
LAMA4	2091.134388	3966.510215	1.89682224	9.77E-27
COTL1	4501.312875	8534.957038	1.89610393	1.09E-30
RAD9A	143.9677365	272.7247451	1.89434627	2.75E-06
CECR5	599.8434548	1135.810735	1.89351192	1.17E-14
ITGA1	144.8597661	274.2822976	1.89343325	3.93E-07
POP7	384.5544988	727.9047334	1.89285195	1.45E-09
CYP26B1	53.0199697	100.3109781	1.8919471	0.001292
NUP35	489.8062794	926.5419913	1.89164988	3.05E-15
TMEM198	60.40580366	114.1708204	1.89006376	0.0006415
MAPKAPK3	233.1699807	440.6806106	1.88995431	8.70E-10
LOC728192	68.76963311	129.9516356	1.88966597	0.0002758
GATA2	194.8769767	368.2379813	1.88959203	3.61E-07
ZNF697	268.2573462	506.8930299	1.88957744	1.11E-10
MAP2K3	900.6984755	1701.907155	1.88954151	4.05E-19
MCM2	2975.166388	5621.36042	1.88942724	1.51E-28
HOMER1	424.2010212	801.4777695	1.88938199	5.21E-14
WDR75	1418.626511	2680.242479	1.88932214	9.50E-24

TRIM5	1177.774263	2224.814688	1.88899924	2.47E-22
FAM64A	1587.561234	2998.029924	1.88844994	1.16E-24
KLF16	563.7058825	1063.483724	1.88659327	2.69E-09
THOC6	490.1088791	924.186773	1.88567645	4.91E-10
GALE	630.3819793	1188.588091	1.88550455	1.60E-11
TIMM50	1265.757095	2386.441206	1.88538639	7.78E-23
APEX2	474.7037909	894.9166355	1.88521064	7.70E-15
CDC25A	324.9719522	612.211773	1.88389111	6.80E-12
PARD6A	121.3349914	228.5516639	1.88364182	2.92E-06
ODC1	12941.09738	24372.38158	1.8833319	9.24E-33
ANK1	122.9257755	231.4826922	1.88310947	2.63E-06
AMH	46.22973113	86.84760219	1.87860929	0.0028043
NHP2	683.2540878	1282.366798	1.87685199	1.57E-17
BCS1L	350.331654	657.3311842	1.87631114	2.73E-12
NUDCD1	1218.636046	2285.004554	1.87505085	3.96E-22
PFDN2	1228.924207	2304.228482	1.87499641	3.00E-22
CABLES1	139.1988229	260.606532	1.87218919	9.98E-07
POLA2	450.5249639	843.2319069	1.87166522	3.94E-14
ECSIT	409.0916554	765.0499387	1.87011866	1.80E-12
PA2G4	3376.311713	6308.790172	1.86854494	4.16E-28
ZFAND2A	320.3233908	597.7359751	1.86603911	1.61E-11
EXOC6	1110.543834	2070.312733	1.86423324	4.98E-21
KLHL25	94.00554409	175.1937645	1.86365353	4.52E-05
CD97	2169.203871	4042.3062	1.8634976	1.14E-25
LPXN	98.24075763	182.9461146	1.86222215	3.08E-05
CTGF	10144.60299	18881.02198	1.86118885	2.79E-31
CCL2	385.9802285	718.2880363	1.86094516	1.02E-12
CENPH	713.9239921	1328.415973	1.86072465	9.42E-12
MTHFD1L	860.5911762	1601.091192	1.86045504	6.65E-19
TMEM109	1054.675844	1961.848205	1.8601433	1.57E-20
CCDC137	664.343488	1234.889802	1.85881223	7.63E-17
NOP58	2203.564973	4094.680076	1.8582071	1.87E-25
CENPW	651.1557269	1209.364839	1.85725901	1.13E-16
SNHG7	501.1721562	929.9898372	1.8556295	2.34E-09
PPM1K	251.4677919	466.4439098	1.8548853	1.05E-09
LRWD1	324.7408519	602.3472738	1.85485525	0.0001415
PTTG3P	300.3694616	557.0461524	1.85453657	7.58E-11
MUTYH	396.0517555	734.2812411	1.85400325	1.40E-10
ZNF598	482.3681541	894.2440541	1.85386213	3.56E-05
CTPS	3549.892269	6580.049403	1.85359129	1.22E-27
CMAHP	50.38241748	93.3798737	1.85342186	0.0023941
TESK2	101.2916577	187.5550467	1.8516337	2.94E-05
UTP15	582.3173211	1077.954788	1.8511467	1.43E-15
TMEM97	1094.687573	2025.629934	1.85041831	2.21E-20
IFITM2	245.2559555	453.5964895	1.84948206	2.00E-09
RECQL4	493.4314312	911.8231243	1.84792266	0.0003369
TPRN	188.097054	347.5744855	1.84784651	5.34E-06

TMEM140	224.7717649	415.2854649	1.84758733	6.97E-09
EMP1	4792.018704	8852.515698	1.84734582	1.62E-28
MAD2L2	1061.951641	1961.515464	1.84708549	4.14E-20
RPIA	458.9438115	847.6662284	1.84699348	1.28E-13
PRRG4	474.6659659	875.8342721	1.8451592	6.77E-14
NRIP1	1286.861663	2374.266367	1.84500513	2.06E-21
DKC1	2395.811275	4418.24709	1.8441549	2.83E-25
FANCG	740.8710393	1365.97421	1.84374086	3.19E-17
PCSK1	91.91200775	169.4237463	1.84332549	7.51E-05
SEPX1	451.2202797	831.1750527	1.8420605	9.32E-13
SRM	1047.892482	1930.166224	1.84195064	9.83E-20
PISD	619.7595591	1141.545341	1.84191647	8.73E-16
RANGAP1	3343.436812	6156.624503	1.84140597	1.30E-14
THOC4	1463.674656	2694.699345	1.84105076	3.38E-22
PPP2R2B	271.9691756	500.4882093	1.84023873	7.22E-10
FAM43A	438.9692505	807.2666345	1.83900497	4.04E-10
LSM2	813.7486565	1492.416641	1.83400197	1.25E-17
ADSL	2029.430084	3719.983014	1.83301856	7.35E-24
MYD88	1709.581937	3133.470815	1.83288718	8.01E-23
RNF126	372.5166649	681.0177466	1.82815377	3.99E-06
ABHD11	344.509095	629.6752251	1.8277463	3.46E-11
HLA-F	523.2058673	954.5283248	1.82438383	8.50E-08
MIS18A	281.4691777	513.4536144	1.82419126	6.68E-10
TRMT6	880.4488238	1605.664711	1.82368886	6.21E-18
C19orf47	248.0138599	452.0955628	1.8228641	0.000201
FARSA	1434.96762	2614.655396	1.82210062	2.30E-18
TDRD7	1328.260586	2420.126818	1.82202713	9.82E-21
WDR3	2143.090397	3903.438847	1.82140653	1.03E-23
MASTL	1568.200173	2855.341839	1.82077638	8.48E-22
MRPS2	764.6880979	1392.072617	1.82044499	8.61E-17
ACD	360.9024947	656.4108006	1.81880372	9.78E-09
DDIT3	1076.585764	1957.900107	1.81861973	3.99E-19
NOB1	895.3786688	1626.302261	1.81632902	7.96E-18
TRIM38	1159.290765	2105.121652	1.81587028	1.38E-19
CRISPLD2	68.24968766	123.9102353	1.81554289	0.0013415
IRF9	2025.752641	3677.631768	1.8154397	3.64E-23
NFKBIB	538.8007922	978.0402767	1.81521685	4.54E-14
C14orf23	1155.069306	2093.583982	1.81251806	1.96E-19
CCT5	12220.00289	22147.35932	1.81238577	5.37E-29
TEX10	707.2472285	1281.326063	1.81170885	6.43E-16
PEMT	248.4444009	450.0849181	1.81161224	6.41E-09
WDR77	1288.13882	2332.970055	1.81111695	3.68E-20
MCM7	4794.317846	8678.135994	1.81008775	7.72E-27
RGS7	200.5482358	362.9918803	1.80999787	9.53E-08
GPR172A	838.5120511	1517.160419	1.80934838	1.28E-05
WDR62	841.9728604	1522.626009	1.80840272	4.45E-12
NCAPH2	914.0444138	1650.449015	1.80565516	9.47E-11

PTRH2	632.1832318	1141.035622	1.80491282	4.02E-12
POLD1	780.2582406	1408.275845	1.8048843	5.02E-05
MRPL12	1110.106416	2003.010052	1.80434058	6.79E-19
NSUN5	168.0709134	303.2220607	1.80413169	8.01E-07
CCDC101	210.6403946	379.9337816	1.80370808	6.30E-08
DPM2	660.0463791	1190.478385	1.80362838	3.39E-15
POLR1C	671.6399175	1211.097087	1.80319403	3.10E-09
PKN3	138.7098252	250.0435049	1.8026373	1.23E-05
BAK1	213.7703832	385.0005389	1.80100037	5.70E-08
CDC45	1040.73431	1874.302075	1.80094195	2.25E-18
F2RL2	59.34528095	106.8739291	1.80088336	0.0019664
NRP1	6490.072436	11687.28363	1.80079402	3.11E-27
TK1	1881.663129	3387.837671	1.80044856	7.72E-11
SH3GL3	75.62176708	136.1511662	1.80042297	0.0005675
SPATS2L	6220.18992	11190.6044	1.79907761	5.18E-27
H1F0	1102.214391	1982.032662	1.79822789	1.15E-18
GLA	545.5532058	980.1524259	1.79662115	1.23E-13
NFKB2	566.1268008	1015.799071	1.79429603	6.53E-14
QTRT1	344.0957472	617.3658357	1.79416875	4.66E-07
DCAF4	54.61763102	97.92981343	1.79300734	0.003206
CCT3	12311.23464	22072.18833	1.79284929	4.10E-28
C1orf109	584.920487	1048.432116	1.79243528	4.41E-14
PLK1	2358.670925	4227.069502	1.79214042	4.58E-23
A4GALT	319.9031657	572.4942267	1.789586	4.75E-10
PPP1R14B	587.1997096	1050.782601	1.78948079	4.79E-14
C17orf53	236.8474238	423.4319148	1.78778349	2.51E-08
ZNF259	816.9921201	1460.425499	1.78756375	3.08E-16
CD83	273.5324506	488.7357832	1.78675613	5.25E-09
GADD45GI	589.9301049	1054.041637	1.78672292	6.20E-06
DDX55	601.7581822	1075.082753	1.7865694	4.03E-14
POLR1B	925.0568231	1652.037247	1.78587651	4.60E-17
DUSP7	538.8420558	962.0777514	1.78545409	2.64E-13
GPN3	751.4074937	1341.26576	1.7850045	1.53E-14
ULBP3	139.1094185	248.2381503	1.78448126	0.0001649
SLC45A4	112.3893211	200.468559	1.78369757	4.69E-05
CLMP	560.724467	999.4707588	1.78246326	1.66E-13
TMEM200I	74.04129893	131.9670133	1.7823433	0.0007733
TUBA4A	96.60183271	172.1093389	1.78163637	0.0001479
ALDH1B1	738.2885052	1314.815766	1.78089697	2.51E-15
APRT	710.3833825	1264.832521	1.78049283	9.66E-05
RPS6KA1	985.6517516	1754.526466	1.78006731	2.98E-17
RSL1D1	3888.396558	6921.384213	1.78000986	1.17E-24
C18orf45	60.39892639	107.4544722	1.77907918	0.002355
GSG2	376.4699105	668.7485029	1.77636641	9.72E-11
SGTA	1783.894176	3168.678482	1.77627043	1.87E-10
PRELID1	1014.613247	1800.134382	1.77420745	2.98E-17
CA2	65.09906725	115.4522578	1.77348559	0.0017407

THOP1	1031.19921	1828.757885	1.77342832	8.35E-10
URB2	624.3909273	1106.896918	1.77276266	5.69E-14
C7orf68	110.3164165	195.3994351	1.77126344	6.46E-05
USP36	1018.44199	1803.799351	1.77113608	2.74E-12
DDX31	397.0400669	703.1514897	1.77098371	5.68E-11
CLP1	199.5392926	353.3775497	1.77096724	3.41E-07
FAM100B	856.2727237	1516.322693	1.77084082	5.40E-09
MCM10	691.8696493	1225.159853	1.77079578	1.78E-14
S1PR2	69.28957856	122.6571108	1.77021009	0.0013434
TRIM25	4492.051562	7949.384154	1.76965559	1.17E-24
FBL	4311.846533	7623.07264	1.76793691	2.09E-24
MRPL38	1036.763871	1832.481846	1.76750164	3.05E-17
UBL7	597.5779868	1055.889504	1.76694846	1.68E-13
NOC3L	1298.279831	2293.488478	1.76655943	1.20E-18
DAZAP1	2081.767781	3677.029926	1.76630168	2.12E-21
DMD	187.95607	331.9470676	1.76608857	7.26E-07
NCLN	1197.813446	2114.967219	1.76569	6.21E-07
PRKCDBP	344.1060631	607.3857182	1.76511193	4.75E-07
SERPINE1	41087.01783	72493.92352	1.76439974	1.03E-27
ZBTB2	809.0038134	1426.086237	1.76276825	2.12E-15
ST3GAL4	612.8420911	1078.905852	1.76049568	1.64E-13
SURF2	274.1796255	482.5055731	1.75981557	1.27E-08
NDOR1	432.3715754	760.6817092	1.75932405	0.0004141
TRIP13	1314.301147	2311.997398	1.7591078	2.02E-18
BMP2K	457.3117639	803.681957	1.75740495	1.68E-11
HSPE1	684.3593127	1201.452077	1.75558666	2.44E-06
TGFBR3	142.8384411	250.6499944	1.75477968	1.55E-05
PRELID2	85.06675104	149.2463886	1.75446208	0.0005373
ERRFI1	6164.787568	10815.59345	1.75441462	7.73E-25
TFB1M	791.8421749	1388.837161	1.75393179	4.72E-15
RASL11A	74.03098303	129.8312843	1.75374254	0.0011836
CEP128	281.9341051	494.4113965	1.75364168	1.18E-08
THOC5	1107.386336	1941.439611	1.75317281	3.96E-17
ARNTL2	448.9685662	786.3671692	1.75149716	2.59E-11
ADD2	1004.004581	1758.307053	1.75129386	1.81E-16
PSMB3	2655.084414	4649.595318	1.75120433	8.86E-22
RDH13	110.8398006	194.0873182	1.7510616	9.27E-05
TMEM201	567.2698508	993.0895181	1.75064745	8.23E-13
SMAGP	657.5051086	1150.397418	1.74964027	1.06E-13
ZMAT5	302.518016	529.0244064	1.74873686	5.32E-09
VARS	1798.215383	3142.495136	1.74756326	7.80E-20
C6orf141	210.495972	367.7494766	1.74706182	7.33E-06
RNH1	1358.0783	2372.633257	1.74705189	1.55E-05
NCEH1	2060.361325	3599.539472	1.74704283	1.68E-20
ITGA2	3081.586713	5382.937243	1.746807	2.79E-22
FANCA	679.0051197	1185.83404	1.74642872	7.46E-14
GAMT	500.1494585	873.3988481	1.7462757	1.80E-06

EXOSC4	263.2917187	459.5151719	1.74527013	0.0032444
GLRX2	283.0462073	493.6491432	1.7440585	1.63E-08
ESRRA	289.4918707	504.4835523	1.74265188	1.38E-05
CPSF4	466.3193296	812.1021554	1.7415151	2.56E-11
PPRC1	1114.868452	1940.967587	1.74098351	7.57E-17
POLR3A	1324.266076	2304.875117	1.74049246	7.46E-18
IL6	108.6774916	189.1409121	1.74038717	0.0001274
ZSCAN12	120.8150459	210.085256	1.73889977	6.67E-05
TUBG1	1612.731811	2802.682467	1.73784782	7.79E-19
PRPF4	1699.682341	2951.353046	1.73641449	4.95E-19
STC2	453.6721457	786.7305896	1.73413906	5.08E-11
GAN	140.1940116	242.9613698	1.73303672	2.36E-05
PSMG3	744.1832755	1289.597597	1.73290322	4.84E-14
C9orf89	162.3102499	281.2393484	1.73272698	6.44E-06
C8orf55	323.1431906	559.5807144	1.73168035	0.0002589
PHC2	3704.677716	6415.152017	1.73163565	2.70E-22
LSG1	3389.403192	5868.180301	1.73133144	5.23E-22
DUSP6	1728.020732	2991.511852	1.73117822	5.60E-19
C16orf59	149.7490318	259.1716973	1.730707	0.0002136
FSD1	323.0434702	559.0001713	1.7304178	4.73E-09
RP9	189.9773951	328.6172064	1.72977004	5.90E-05
FKBP4	4126.169686	7132.000485	1.72847969	1.61E-22
LIF	1759.423065	3039.347535	1.72746828	6.06E-19
DCBLD2	24753.10768	42728.02781	1.72616822	1.96E-25
EED	458.9713206	792.1655004	1.72595861	6.64E-11
SLIRP	849.0320241	1464.451521	1.72484839	1.23E-14
MRPL14	729.408169	1257.455424	1.72393932	1.20E-13
CCDC58	384.3550581	662.3059032	1.72316167	8.07E-10
MRPL39	413.2202713	712.0059335	1.72306632	3.35E-10
AHCY	1922.525624	3312.553685	1.72302186	3.07E-19
MAPK8IP2	143.4237206	247.0794306	1.72272362	0.0002648
OXNAD1	223.0881377	384.1745602	1.72207525	4.32E-07
SERPINB8	649.0243656	1116.917181	1.72091718	8.49E-13
CCDC138	247.3804396	425.6006898	1.72042984	1.56E-07
LAMB3	631.3118339	1086.124818	1.72042525	1.04E-07
GINS4	922.1606616	1585.423628	1.71924882	5.25E-15
TICAM1	264.7243257	454.9959116	1.71875369	7.52E-08
RPA2	2138.166033	3674.250014	1.71841193	1.68E-19
MED12L	329.2071657	565.6858401	1.71832785	6.37E-09
KIF18B	839.4797307	1442.22574	1.71799948	1.67E-07
RAB3B	222.6438422	382.4117177	1.71759396	6.04E-07
TRAF4	1051.511469	1805.656598	1.71720105	1.18E-15
CISD3	548.9032669	941.5086604	1.71525425	7.16E-09
PRPF19	3221.996647	5526.446659	1.71522421	4.24E-21
DRAP1	1103.402143	1892.128948	1.71481355	6.62E-16
CH25H	147.0599001	252.1768675	1.71479014	2.19E-05
HIVEP3	1356.905014	2325.125667	1.7135508	5.40E-17

DIEXF	880.3594193	1506.616324	1.71136503	2.13E-14
AMDHD2	75.11213753	128.5168009	1.71099912	0.0018126
FBF1	243.6926805	416.5008104	1.70912319	2.48E-07
GEMIN5	938.2961638	1603.349639	1.70878844	9.67E-15
AFMID	391.2037534	668.4133954	1.70860681	1.30E-09
TJP2	389.5854603	665.5791385	1.7084291	3.87E-09
CDC42EP1	1016.241144	1734.662917	1.70694026	0.0012741
THAP7	315.2099021	537.9378426	1.70660198	2.78E-06
FAM195A	117.6816187	200.5322844	1.70402385	0.00137
FARSB	2227.705975	3795.127802	1.70360355	1.03E-15
ATRIP	184.8329587	314.7904101	1.70310756	4.76E-06
USP53	926.1689253	1576.529124	1.70220473	2.18E-14
KLF10	492.9500225	838.533303	1.70105135	9.00E-11
TSR1	2876.407109	4888.620251	1.69955784	5.28E-20
RIMKLA	224.6857991	381.8288081	1.69939004	9.14E-07
TMEM138	416.9011531	708.0931627	1.69846775	8.51E-10
CCDC99	3735.892348	6343.023282	1.69786029	8.18E-21
RBM15	540.804924	917.9565629	1.69738943	8.75E-11
REXO1	263.766962	447.4866307	1.69652267	0.0006478
C6orf174	184.2889428	312.5909556	1.69620028	5.68E-06
ELL2	905.7638233	1535.518308	1.69527449	3.90E-14
POLR3B	359.1776039	608.79224	1.69496158	6.29E-09
AATF	2638.977132	4472.52064	1.69479325	1.86E-19
PSME2	1554.395473	2634.028074	1.69456751	5.00E-17
UTP18	1501.437399	2543.836411	1.69426738	6.52E-17
ZNRD1	321.4182999	544.5291065	1.69414469	2.11E-08
KIF6	111.8315507	189.4477066	1.69404524	0.0002556
KHK	116.5695165	197.2307361	1.69195809	0.0001961
DOCK6	583.6804436	987.2274614	1.69138348	1.05E-05
GINS3	659.9329042	1115.838667	1.69083654	3.52E-12
SELRC1	622.221741	1051.950787	1.6906365	8.20E-12
SFR1	263.5950303	445.2612298	1.68918674	2.42E-07
DUSP10	284.5303937	480.5822337	1.68903655	1.15E-07
TRMT1	507.8420426	857.6722922	1.68885642	1.09E-10
GTPBP4	2009.837923	3392.9941	1.68819289	4.44E-18
TUFT1	782.9824705	1321.343304	1.68757712	4.38E-13
LTV1	1312.514361	2214.52541	1.68723899	6.63E-16
CCT4	6150.817814	10375.37066	1.68682783	1.11E-21
UBXN8	202.1287039	340.8015114	1.68606192	3.68E-06
KIAA0922	240.9932329	406.2728907	1.68582697	5.84E-06
DLL1	96.13690539	162.0678626	1.68580278	0.0007635
PLK3	236.8199148	399.0444579	1.68501225	8.22E-07
TREX1	372.9953467	628.4244671	1.68480511	9.56E-06
TMEM11	715.1908328	1204.916403	1.68474811	1.77E-12
LGMN	778.0863275	1310.478216	1.68423242	6.24E-13
CDKN1A	838.2369605	1411.702478	1.68413294	2.23E-13
GET4	370.7848969	624.358299	1.68388277	6.54E-09

APOL1	1805.102615	3039.555191	1.68386837	1.98E-17
ASRGL1	181.6788997	305.881707	1.68363914	7.10E-05
FOXRED2	1874.553809	3155.212825	1.68318072	1.69E-17
MAD2L1BP	790.9157589	1331.172391	1.68307734	5.05E-13
CASP1	205.7923925	346.2340557	1.68244342	3.38E-06
BCL2L1	3012.798463	5067.679627	1.68205065	2.05E-19
CD274	1224.937287	2060.273623	1.68194212	2.27E-15
CENPM	238.5035419	401.0834156	1.6816665	0.0007365
BCL3	248.9746622	418.3368445	1.68023863	5.73E-07
STAMBPL1	304.6012365	511.6648254	1.67978578	8.60E-05
BOLA3	353.4238176	593.6533268	1.67972077	1.50E-08
ATP5D	342.6081221	575.3615296	1.67935753	0.0027622
DSCC1	536.6350445	900.5356231	1.67811557	1.04E-10
NUP85	1314.462763	2205.406684	1.67780081	1.44E-15
EIF6	2231.503058	3741.927948	1.67686436	4.61E-18
RPS5	6740.988503	11302.81884	1.67673018	3.22E-10
BRMS1	676.5016742	1134.182357	1.67654036	5.48E-12
C1QBP	4577.163016	7669.473489	1.67559544	1.96E-20
CAMK4	147.5488979	247.1172097	1.67481569	0.0001778
MRPL24	1727.69335	2892.288854	1.67407535	8.86E-17
SDC1	4540.255781	7600.558825	1.67403759	8.63E-14
LOC401022	122.8535641	205.5659958	1.67326033	0.0002086
C11orf48	742.0278438	1240.390804	1.67162299	2.55E-12
PRMT1	5767.67932	9640.808573	1.67152299	7.27E-21
FLAD1	862.746608	1441.508365	1.67083632	4.48E-13
STIP1	5642.84783	9427.542035	1.67070641	8.15E-21
FAR2	168.0124567	280.5408205	1.66976203	2.56E-05
PXN	5461.656505	9116.088586	1.66910691	1.14E-20
PRKD2	939.576759	1567.901184	1.66873134	1.36E-13
PRMT7	368.040747	614.104433	1.66857729	1.49E-08
TOMM5	1680.950551	2803.874233	1.66802898	1.82E-16
EIF3B	5783.506057	9646.233933	1.66788689	9.47E-21
FAM54A	284.1101686	473.7714805	1.66756256	2.30E-07
RPS19BP1	722.3214961	1203.656179	1.66637181	6.15E-06
HIST1H2BD	145.975307	243.14308	1.66564527	7.39E-05
HPRT1	1387.16501	2309.649279	1.66501409	2.02E-15
TRIM14	2656.441371	4422.08667	1.66466564	2.84E-18
RUSC1	995.7714195	1657.528784	1.66456754	9.08E-14
TRAF3IP2	253.7091894	422.3015081	1.6645101	1.11E-06
HNRNPL	4095.767995	6813.285557	1.66349402	1.28E-19
SRFBP1	425.1515076	707.1137867	1.66320423	3.69E-06
GTPBP5	471.2051567	783.6744769	1.66312797	1.50E-06
RFC2	1190.783215	1980.267453	1.66299577	1.40E-14
SLC9A2	226.8309149	377.1585171	1.66272978	2.56E-06
C6orf1	437.1164185	726.2268296	1.66140369	5.22E-05
INTS5	265.2511484	440.6806106	1.66137117	5.58E-07
SPRY2	1255.534268	2085.234523	1.66083442	7.48E-15

WBSCR22	1467.228308	2435.758969	1.66010903	1.62E-15
GUSBP1	79.28545568	131.570547	1.65945375	0.0028777
STIL	1977.284951	3280.614521	1.65915111	7.80E-17
PRKCE	201.584688	334.2999193	1.65835968	8.32E-06
DDX56	1331.696613	2208.229108	1.65820735	4.80E-15
EXOSC9	749.8614119	1243.166069	1.65786111	5.07E-12
PPP1R15A	1782.307542	2954.3336	1.65758913	2.23E-16
PHLDB2	363.3027812	601.9838534	1.65697563	2.70E-08
CCDC22	142.3184957	235.7871963	1.65675723	0.0001091
CNNM4	703.0807876	1164.62068	1.65645357	1.22E-11
PTTG1	2041.212748	3380.005115	1.65588086	7.15E-17
B3GNTL1	92.96565318	153.9143131	1.65560406	0.0013403
CRSP8P	162.2345999	268.5712717	1.65545002	4.33E-05
PHF5A	1071.820289	1774.312091	1.65541939	9.13E-14
TMEM14B	662.6220359	1096.791716	1.65522976	2.76E-11
NDRG1	1285.439372	2125.759201	1.65372187	1.17E-14
DDX54	1079.928948	1785.457942	1.65331057	3.00E-08
DHRS7B	415.985053	687.5288049	1.65277286	9.47E-06
PRIM1	700.3160059	1157.137346	1.65230744	1.56E-11
TMEM48	2048.764348	3383.660618	1.65156165	9.67E-17
PELO	836.0608969	1380.38155	1.65105383	2.00E-12
TRIM26	1832.542099	3024.319592	1.65034113	3.69E-16
RPL39L	91.87762141	151.6251868	1.65029508	0.0015686
MAD1L1	410.696194	677.62416	1.6499402	8.53E-09
EHD1	331.8584725	547.4908143	1.649772	8.74E-08
TAGLN2	6142.214583	10131.45358	1.64947894	5.67E-20
C11orf84	458.0999227	755.3176234	1.64880539	7.78E-06
DGCR14	345.5902495	569.775588	1.64870273	5.95E-08
RFC3	1332.037749	2195.808833	1.64845841	1.08E-14
RFC4	2689.373304	4432.906879	1.64830478	1.05E-17
PTTG2	509.2402632	839.2955562	1.64813275	8.76E-10
ADAM15	1074.643527	1770.911404	1.6479059	1.28E-13
TRIM11	415.0070575	683.6679268	1.64736458	4.52E-05
NOP14	1538.711855	2534.085163	1.6468874	1.34E-09
FOXRED1	430.109546	708.0341703	1.64617172	6.76E-09
SH2B3	1287.980643	2120.038709	1.64601752	1.82E-14
POLE3	3122.715998	5138.040786	1.64537562	5.07E-18
ZNFX1-AS1	1556.544027	2561.075726	1.64536029	2.90E-15
GEM	1708.504221	2810.739245	1.64514621	1.02E-15
BCKDK	842.8889605	1386.460729	1.64489131	2.87E-12
KIF2C	3562.342739	5855.649142	1.64376355	2.14E-18
ATP5SL	827.7520856	1360.024849	1.64303403	3.97E-12
POLD2	1974.674196	3244.071072	1.64283864	3.02E-16
ZNF205	119.2758414	195.9186192	1.64256749	0.0021764
MECR	369.1975514	605.8942577	1.64111126	4.58E-08
ADRA1B	204.8109584	336.0981744	1.64101656	1.08E-05
ICT1	753.4597664	1236.206652	1.64070692	1.40E-11

SUV39H1	350.2422495	574.4175661	1.64005789	7.72E-08
CBR3	305.1349365	500.4221173	1.64000269	3.11E-07
MSTO1	154.8969069	253.8925503	1.63910665	9.79E-05
RAD51D	381.7037513	625.4273468	1.63851506	3.37E-08
ALG10	108.178178	177.2398217	1.63840642	0.000876
MACROD1	102.9752848	168.6898059	1.63815819	0.0010522
RCC1	3762.801571	6162.451147	1.6377295	2.41E-18
DGKG	543.9624217	890.7985747	1.6376105	7.53E-10
C8orf46	229.451274	375.6269109	1.63706614	4.43E-06
CCT2	8342.720476	13657.24769	1.63702568	5.55E-20
KAT2A	1579.762052	2585.784091	1.63681871	4.13E-15
MRPL46	427.9713074	700.465897	1.63671229	1.15E-08
PODXL2	570.8097485	933.8034701	1.63592768	4.16E-10
POC5	443.1288141	724.1524595	1.63418048	8.61E-09
NUP155	1107.9613	1810.270264	1.633875	2.84E-13
LOC100507	728.2066623	1189.775124	1.63384268	3.15E-11
ATG16L1	1032.459885	1686.688036	1.63365963	5.58E-13
DCLRE1B	541.8792013	885.2456791	1.63365871	1.01E-09
TMEM170A	160.698834	262.4968254	1.63347063	7.72E-05
CYC1	2271.152308	3709.483713	1.63330469	1.51E-12
C17orf61	347.6941017	567.7956228	1.63303208	1.14E-07
WDR46	1380.711757	2254.384521	1.63276984	2.25E-14
FRRS1	133.8687004	218.5691799	1.63271309	0.0002606
IFRD1	2763.525351	4511.086481	1.6323666	3.77E-17
RIPK2	956.2184521	1560.427316	1.63187325	1.48E-12
E2F2	534.6481058	872.3935258	1.63171536	1.27E-09
CCDC150	148.0963524	241.6445198	1.63167098	0.0003423
FAM98C	184.3611541	300.3854372	1.62933151	3.60E-05
GTPBP3	161.7937431	263.4455219	1.62828004	8.72E-05
B7H6	271.4595461	441.9030557	1.62787812	1.45E-06
RABGGTA	194.9010471	317.0819029	1.62688661	4.52E-05
EME1	601.6722164	978.165361	1.62574461	4.49E-10
HAUS5	538.3668126	875.0366063	1.62535391	9.17E-07
TRMU	444.2581095	721.648577	1.62439033	1.28E-08
RUVBL2	2781.693798	4518.270121	1.62428738	1.77E-13
CHEK1	1041.157974	1691.004373	1.62415735	1.16E-12
PSMC3IP	839.4838812	1363.128121	1.62376926	1.03E-11
RTTN	254.6837463	413.5131562	1.62363387	2.91E-06
MFSD2A	553.0318828	897.9113893	1.62361596	1.26E-09
COMMD4	463.3509568	751.6196088	1.62213889	0.000166
C13orf27	554.9603648	899.95508	1.62165649	1.34E-09
KRT7	106.119028	172.0456135	1.62125131	0.0016612
PSMB8	2423.563674	3929.074695	1.62119722	2.96E-16
CHEK2	387.4609763	627.5347629	1.61960765	6.55E-08
BCL7B	1090.796223	1766.63758	1.61958535	8.09E-13
MYBL2	1947.802799	3153.763791	1.61913916	2.39E-15
DNMBP	1663.218099	2692.934136	1.61911065	1.09E-14

MRPL1	469.4596341	759.8604636	1.6185853	8.15E-09
SH3D21	95.59976677	154.7072458	1.61828058	0.0019991
DDX51	469.5834249	759.586715	1.61757565	2.44E-06
DPF1	135.5076253	219.1520895	1.61726758	0.0003496
CIDECP	229.4925376	371.0227119	1.61670927	9.07E-06
CRY1	546.0146945	882.7394301	1.61669537	2.12E-09
RPSA	772.1908454	1248.176201	1.61640896	3.37E-07
ITGA3	21703.55161	35076.89294	1.61618216	4.60E-20
C2orf3	576.4672531	931.5190768	1.61590979	1.19E-09
RPAP1	470.6336317	760.4740526	1.61585148	6.32E-08
KCTD5	953.2879042	1540.065881	1.61553071	4.43E-12
PRR3	400.6796851	647.2283489	1.61532609	5.95E-08
HSPD1	10037.02455	16208.69506	1.61489045	3.54E-19
FREM2	275.0269529	444.1048767	1.61476856	0.0001346
ASF1B	2242.855892	3621.158764	1.61453029	1.06E-15
HYAL2	512.0635016	825.8345468	1.61275807	4.74E-09
DEDD2	648.1185814	1045.196659	1.6126627	3.59E-10
GADD45B	384.4891648	620.025482	1.61259546	9.25E-08
DNAJC2	1015.870483	1637.478877	1.61189729	3.06E-12
MKI67IP	1244.295621	2005.476156	1.61173609	3.56E-13
NOLC1	4367.611365	7034.172091	1.61053068	1.43E-17
DDI2	116.0289392	186.8211063	1.61012509	0.0008792
DVL1	621.5704157	1000.412356	1.60949159	0.0015929
DUS3L	282.1335458	454.0543147	1.60935954	0.000366
ACTR5	159.1114886	256.0259128	1.60909759	0.0001457
LOC100132	175.9182361	283.0140236	1.60878161	8.04E-05
RNF181	744.2761186	1196.767587	1.60796183	1.13E-10
SMPD2	173.831577	279.3490549	1.60700984	8.68E-05
PER1	278.9347845	448.1851585	1.606774	0.0010793
MRRF	597.0442868	959.2977538	1.60674472	1.29E-09
C1orf74	178.5695428	286.8276564	1.60625184	7.27E-05
E2F7	1330.598265	2136.716328	1.60583129	2.91E-13
BAG3	4227.412491	6782.375124	1.60437978	2.83E-17
AURKA	2504.602241	4017.947141	1.60422564	1.11E-15
E2F4	1692.605984	2715.001787	1.6040365	2.90E-14
DFFA	989.1194381	1586.433684	1.60388485	6.56E-12
UBE2T	1891.773905	3033.884397	1.60372463	6.74E-11
SLC39A3	376.5730695	603.784475	1.60336605	1.64E-07
CDCA8	2161.133037	3464.719355	1.60319578	4.17E-15
NUFIP1	146.9980047	235.6054861	1.60278016	0.0002881
PPIF	1926.192752	3086.430431	1.60234765	1.15E-14
SEMA6B	207.9650174	333.2261385	1.60231823	2.61E-05
PDK1	366.4430857	587.1210553	1.60221622	2.47E-07
RAN	5556.909681	8902.574416	1.6020729	1.03E-17
TATDN1	329.3137634	527.3795486	1.60145007	6.57E-07
CSTF2	696.1942673	1114.248069	1.60048441	3.40E-10
INO80E	775.2589386	1240.508789	1.60012188	1.11E-10

B4GALT3	954.768652	1527.496943	1.5998608	1.37E-11
RPLP2	1128.902829	1805.894934	1.59969033	1.79E-06
EEF1E1	413.7574099	661.7890855	1.59946159	8.49E-08
NME6	171.7655497	274.7094434	1.59932794	0.000113
DTYMK	868.775485	1389.28552	1.59913067	7.09E-07
OIP5	403.2897282	644.8424511	1.5989558	1.02E-07
PPDPF	591.6274866	945.9570957	1.5989066	0.0014852
ERAL1	1742.306841	2785.013725	1.5984634	3.99E-14
FGF13	495.060752	791.2828958	1.59835514	1.50E-08
ABHD8	234.8364147	375.1997651	1.59770692	0.0004075
FLYWCH1	965.5217403	1542.59571	1.59768097	2.89E-06
GMNN	1652.622476	2639.998649	1.59746021	6.17E-14
PMF1	260.4719189	415.9863593	1.59704878	5.64E-06
FAM86DP	344.4024973	550.0253762	1.59704236	4.78E-07
OGFR	821.0753219	1310.841636	1.59649377	3.51E-06
AURKAPS1	226.3006536	361.1912587	1.59606812	1.77E-05
RASSF1	246.805476	393.8266699	1.59569664	9.63E-06
SESN2	509.0194788	812.2012934	1.59561928	0.0005339
MED22	529.9961058	845.1552463	1.59464426	1.05E-07
ACTL6A	5037.591014	8032.998667	1.59461112	3.50E-17
ABL2	2066.900543	3295.059639	1.59420329	3.57E-14
DDX49	672.3902514	1071.911022	1.5941799	6.55E-07
POLR3C	719.9184828	1147.626886	1.59410671	4.12E-10
ADRM1	1825.554434	2908.890829	1.5934287	0.000128
NPTX1	128.6967549	205.0161321	1.59301711	0.0007009
TUBA1C	8681.362901	13828.60669	1.59290734	5.06E-18
SSNA1	606.778116	966.5026067	1.59284355	4.25E-05
LOC100507	142.3219343	226.6896834	1.59279513	0.0004015
FKBPL	200.0557994	318.5733635	1.59242254	4.51E-05
ACOT7	2539.609094	4043.43424	1.59214828	2.46E-07
RAD54L	782.893066	1246.073517	1.59162671	1.93E-10
EPHB2	1424.669143	2267.427851	1.59154696	4.16E-13
ZWINT	2409.52242	3834.668286	1.59146404	4.65E-15
MRPL17	1514.401649	2409.424508	1.59100758	2.17E-13
RFC5	1579.800589	2513.391074	1.59095464	1.86E-13
ZNF267	319.769059	508.3254981	1.58966443	1.19E-06
FANCB	197.9381926	314.6393793	1.58958398	5.75E-05
SLC25A39	2099.610269	3337.021433	1.58935279	3.12E-07
TNFAIP8	222.0826332	352.8866785	1.58898818	2.42E-05
DUS4L	209.9932197	333.6556508	1.58888773	3.72E-05
ABCF1	2504.343632	3978.427785	1.58861098	3.82E-15
HSD17B7	109.7483302	174.3394729	1.58853873	0.001833
DTX3L	6421.452088	10197.23412	1.58799505	1.07E-13
C12orf11	947.6207955	1504.320098	1.58747054	3.13E-11
C19orf28	741.3833957	1176.802619	1.58730641	0.0022864
MCM5	2283.699771	3624.719861	1.58721383	3.72E-13
ORC5	534.992681	848.7022302	1.58638101	1.19E-08

PELP1	1607.604567	2549.809353	1.58609238	6.36E-12
ZWILCH	2124.747883	3367.934062	1.58509821	1.98E-14
EIF3M	7733.320577	12258.02676	1.58509228	1.41E-17
HSPH1	7009.384226	11106.289	1.58448854	2.31E-17
PAK4	1840.322664	2915.493925	1.58422976	0.000136
PUS3	234.7195012	371.8156446	1.58408502	1.94E-05
RAD18	839.962563	1329.88622	1.58326844	1.31E-10
PARP14	9537.991348	15100.29704	1.5831737	9.83E-18
RNASEH2B	458.1625299	725.2828662	1.58302528	0.0004098
JMJD6	696.3696376	1102.125123	1.58267257	8.58E-10
AP1AR	732.2974532	1158.80815	1.58242821	2.30E-05
SLC16A3	784.9618201	1241.96957	1.58220379	0.0016128
SLC25A33	207.8962448	328.8343291	1.58172327	5.04E-05
CHAF1A	1268.636063	2006.044866	1.58126111	2.33E-12
C5orf62	546.1040989	863.2983794	1.58083117	1.36E-08
CDC6	3202.459504	5060.774469	1.58027743	1.73E-15
MRPL21	532.0002377	840.3127113	1.57953447	1.88E-08
CTNNAL1	2609.034203	4118.151882	1.57842004	7.19E-15
KLHDC4	318.1885909	502.1330671	1.57809891	2.06E-06
MRPL2	698.9246626	1102.958201	1.57807881	1.23E-09
TMEM206	729.3772213	1150.852877	1.57785689	7.92E-10
DPP9	1566.584607	2471.254584	1.57747917	5.01E-13
NAT10	2281.848363	3599.414387	1.57741174	2.11E-14
ARHGDIA	5748.646233	9066.723577	1.57719282	3.91E-05
AGPAT9	1117.399407	1759.151879	1.57432684	1.36E-11
FKBP5	692.5168242	1089.98333	1.57394491	1.69E-09
TMEM39B	440.0916686	692.5554166	1.57366173	1.30E-07
RRAS2	541.7382173	852.4356574	1.57351952	0.0001434
CWF19L1	475.2340522	747.5133806	1.57293733	9.73E-08
C1orf174	672.7073175	1057.409277	1.57187123	2.40E-09
C19orf53	1351.990967	2124.843551	1.57164035	1.58E-09
CHCHD8	768.3242774	1207.448599	1.57153514	6.65E-10
C12orf52	455.8585252	716.3411171	1.57141104	1.14E-07
HSPA8	39914.01557	62719.00063	1.57135281	3.65E-18
PPIH	643.3187202	1010.645008	1.57098647	4.05E-09
POLR2D	1225.966862	1925.894765	1.5709191	7.76E-12
OSBPL5	176.5172701	277.2416388	1.57062048	0.0001776
PPAT	707.2025262	1110.467482	1.57022556	1.71E-09
GPS2	1606.799215	2520.761071	1.568809	7.41E-13
SBDSP1	613.7169276	962.4742177	1.56827061	7.61E-09
SPATA5L1	194.2573109	304.597903	1.56801256	0.0001169
POLR2H	2337.353873	3664.82686	1.56793839	4.54E-14
LMNB2	5947.284604	9323.242813	1.56764699	5.56E-12
MRPS30	847.9199219	1328.328668	1.56657325	3.24E-10
CKS1B	352.8660472	552.6283967	1.56611383	1.44E-06
TMEM141	713.682576	1117.011586	1.56513781	3.41E-07
FADD	531.9761673	832.6098874	1.56512629	3.20E-08

CCNB1	6055.984862	9477.23277	1.56493667	2.98E-16
CLTB	1054.07681	1649.356388	1.56474023	4.90E-11
CEP164	595.3675369	931.5804357	1.56471487	1.20E-08
NGDN	742.933628	1162.185256	1.56431909	1.59E-09
IL13RA2	2863.691152	4478.507781	1.56389343	6.84E-13
WDR34	1475.584549	2306.765411	1.56328922	6.64E-06
DOLPP1	634.8001522	992.2021804	1.56301503	6.64E-09
ABCF2	1778.361886	2778.991172	1.5626691	5.38E-13
AUP1	2077.649481	3246.338985	1.56250562	1.73E-13
CENPB	1658.80953	2591.761766	1.56242276	9.62E-08
TXNL4B	658.4005769	1028.653591	1.5623522	5.06E-09
ESPL1	914.2308118	1428.122828	1.56210315	2.10E-10
STOML1	144.9973115	226.4442477	1.56171342	0.0012477
RPL14	2927.636142	4571.101822	1.56136268	1.72E-14
CYB561D2	202.2800038	315.7957324	1.56118117	0.0027174
BORA	528.7842833	825.3814546	1.56090391	4.62E-08
SP140L	456.1927844	712.0625593	1.5608808	1.70E-07
SHISA5	2817.793545	4398.178251	1.56085894	1.96E-14
NUDC	3229.092924	5039.384047	1.56061909	8.47E-15
HMGXB3	1338.493728	2088.354361	1.56022723	7.10E-12
ACP6	582.8888461	909.0596925	1.55957641	2.12E-08
MOCS1	295.1734457	460.2821582	1.55936167	7.25E-06
CCNB1IP1	909.5650572	1418.230016	1.55923978	2.55E-10
SAC3D1	138.6857548	216.2186947	1.55905482	0.0008775
IGF2BP3	914.6991777	1425.899794	1.55887294	2.75E-10
RTN4IP1	194.2676268	302.8232278	1.55879409	0.0001332
RPUSD2	212.6789128	331.4278834	1.55834859	7.69E-05
CHORDC1	764.3167255	1190.896064	1.55811854	4.89E-07
QTRTD1	1423.860352	2218.48534	1.55807789	4.72E-12
GTPBP8	400.1975646	623.5370534	1.55807308	6.08E-07
CDK5RAP1	484.596509	754.8881111	1.5577663	1.06E-07
HAUS8	546.0525194	850.1653778	1.55692969	3.93E-08
MRPS18A	677.9927379	1055.516617	1.55682585	5.02E-09
GGCT	1331.497172	2072.457928	1.55648692	1.07E-11
GBP3	728.6715896	1133.939288	1.55617332	2.70E-09
TIAM1	139.1403662	216.4948097	1.55594538	0.0009597
ZC3HAV1	3559.458317	5536.658013	1.55547769	7.82E-15
QPCTL	500.0531767	777.5410383	1.55491671	9.16E-08
STK10	441.0971732	685.7753429	1.55470355	2.75E-07
C14orf21	295.6452503	459.453813	1.55407135	7.91E-06
RPL23AP82	137.559898	213.7454916	1.55383578	0.0010102
DNAJA3	1853.239484	2879.108607	1.55355454	7.18E-13
GEMIN7	309.3254478	480.4618824	1.55325689	6.41E-06
RPS6KA5	561.754042	872.5186101	1.55320397	4.13E-08
KTI12	271.5111256	421.6950186	1.55314084	1.62E-05
HTRA2	547.722392	850.1111185	1.55208392	5.32E-08
STOML2	2082.298042	3231.33927	1.55181401	3.76E-13

TFPI	247.2566488	383.6860555	1.55177245	3.12E-05
THG1L	354.9148813	550.6389653	1.55146767	2.17E-06
KLHL18	516.243697	800.8335009	1.55127027	8.32E-08
TOP1P1	242.5014898	376.1484617	1.55111815	3.86E-05
NCKIPSD	536.7932217	832.3337724	1.55056685	5.70E-08
C21orf59	870.4391921	1349.612852	1.55049642	7.35E-10
TUBB2C	8305.989203	12878.04979	1.55045347	1.54E-07
TYSND1	297.2532275	460.8556017	1.55038048	8.13E-06
C3orf75	419.5730916	650.4661717	1.55030479	6.08E-07
FAM53C	1485.11621	2302.232037	1.55020329	6.40E-12
FTSJ2	1287.012963	1994.596868	1.54978771	1.90E-11
MARS2	227.3439831	352.247143	1.54940165	6.33E-05
PRMT3	820.0739678	1270.576592	1.54934389	1.31E-09
SIGMAR1	2159.355855	3344.894134	1.54902404	3.53E-13
KARS	4706.200907	7290.00928	1.54902211	3.77E-15
NUP88	2027.791159	3140.713447	1.54883477	6.32E-13
FBXO31	457.0050137	707.8194141	1.54882199	1.48E-06
PTGES2	648.1495291	1003.461369	1.54819424	7.47E-06
THAP4	779.9900272	1207.144171	1.54764052	2.28E-09
UMPS	1183.011542	1830.69069	1.54748337	5.87E-11
LOC727896	193.7373654	299.7718482	1.54731044	0.0001847
ZNF473	574.9796281	889.6398551	1.54725457	4.13E-08
CCT7	7846.543471	12131.18558	1.54605472	7.12E-16
CTU2	234.8260988	363.0485061	1.54603133	0.0026721
GIT1	1972.356437	3048.957132	1.5458449	4.31E-08
ZSWIM4	228.9657149	353.9226803	1.54574531	6.15E-05
SNX11	791.2362636	1222.13442	1.54458848	2.52E-09
TFAP4	194.8494676	300.9282013	1.54441377	0.0001743
PLTP	276.225021	426.5470198	1.54420124	1.83E-05
ZMYND19	568.8159326	877.82607	1.54325155	5.44E-08
OBFC2B	1235.590655	1905.998256	1.54258067	5.47E-11
RPS19	5705.508666	8799.979045	1.54236538	2.60E-05
CCDC107	197.4870198	304.531811	1.54203457	0.0001757
ZC3H8	307.6693297	474.3874362	1.54187431	9.99E-06
NT5C	341.8749814	526.9830823	1.54144968	4.33E-06
KRI1	1357.948344	2092.710758	1.54108274	2.25E-11
NCL	24516.84482	37780.62852	1.54100696	1.28E-16
RGMB	1520.145119	2341.914171	1.54058592	1.06E-11
GNL2	2750.192456	4236.532801	1.54044958	1.34E-13
BST2	7427.119544	11439.59052	1.54024591	1.29E-15
SKA3	864.1620217	1330.837283	1.54003214	1.43E-09
C5orf34	272.461612	419.4011592	1.53930367	2.25E-05
FUS	5855.662865	9011.114416	1.53887179	3.66E-15
SNAPC1	1853.932073	2851.636724	1.53815599	2.48E-12
C9orf114	509.5772493	783.7971947	1.53813224	0.0002465
TPRKB	491.3936248	755.7471358	1.53796691	2.46E-07
RPS6KA4	326.6899657	502.1000211	1.53693126	6.63E-06

CENPO	1215.684866	1868.357446	1.53687645	9.11E-11
NOSIP	967.6152766	1487.094982	1.53686596	5.51E-05
PQLC2	309.90385	476.2470501	1.53675745	1.05E-05
POLQ	1269.021902	1949.189595	1.5359779	6.68E-11
ARPC5L	952.1310998	1462.313425	1.53583201	7.38E-10
UCHL3	484.062809	743.0814256	1.53509299	3.32E-07
JAG1	6722.377896	10317.36833	1.53477958	3.34E-15
PVR	3018.549522	4632.622651	1.53471812	1.29E-13
INPP5F	2185.023019	3353.264807	1.5346588	1.12E-12
CACYBP	2786.343783	4275.778323	1.53454802	1.91E-13
CCT6A	8432.648983	12937.36905	1.53419988	1.89E-15
C15orf23	1653.914099	2536.053382	1.53336463	8.55E-12
PGP	533.6082149	817.7399897	1.53247264	1.37E-07
H2AFX	1331.695901	2040.424273	1.53219986	5.75E-05
ZNF331	475.2478067	727.841008	1.53149788	3.94E-07
SMG5	3298.823359	5050.504037	1.53100166	1.08E-13
APOL6	2935.790215	4494.220138	1.53083831	2.22E-13
CTNNBL1	1122.485386	1718.2473	1.53075249	2.47E-10
C12orf43	418.4541122	640.5403136	1.53073012	1.28E-06
NEDD4L	728.6784669	1114.958429	1.5301103	1.24E-08
ELOF1	1000.051335	1529.904054	1.52982552	7.28E-10
TMEM93	563.5098804	862.0641016	1.52981187	9.68E-08
C12orf44	849.1489377	1298.905133	1.52965525	2.97E-09
NANS	1123.807245	1718.889202	1.52952315	2.62E-10
ZNF530	245.1768669	374.9283831	1.52921598	6.76E-05
DNAJC9	1065.226764	1628.782564	1.52904773	4.77E-10
USP15	1478.984174	2260.555739	1.52845161	3.09E-11
ATP6V0B	2883.629192	4406.603183	1.52814488	3.03E-09
NOP10	1493.40439	2282.092458	1.5281142	3.30E-11
FRMD6	1195.032183	1825.859902	1.52787509	1.87E-10
BCAR3	1280.808004	1956.724822	1.52772688	1.02E-10
GCAT	401.3612463	613.1510034	1.52767864	0.0022276
UBE2M	1931.071701	2950.005516	1.52765198	4.42E-12
RNF144A	127.0647073	194.0590053	1.52724552	0.0025505
SCFD2	280.408655	428.2272901	1.52715432	2.87E-05
MED27	401.2099464	612.5799265	1.52683136	2.14E-06
TAF1A	381.1803673	581.7356707	1.52614279	0.001325
COPS3	3260.262142	4973.591759	1.52551897	2.02E-13
NUDT15	819.622795	1250.278884	1.52543205	5.40E-09
ADCK3	497.3915541	758.1589799	1.52426991	3.99E-07
CALML4	215.3130264	328.1593811	1.5241037	0.0001645
RAD51AP1	914.6372823	1393.660849	1.52373064	2.28E-09
AURKB	1427.775061	2175.357727	1.52359975	5.76E-11
PIF1	235.339167	358.5009329	1.52333731	0.0007391
UTP14A	673.5133813	1025.824067	1.52309382	2.24E-06
SENP3	550.9280306	838.9982278	1.52288172	1.96E-07
DHX33	1829.708544	2786.316376	1.52281979	9.40E-12

LSM4	2110.078662	3212.636807	1.52251992	3.11E-12
NIF3L1	849.510706	1293.349871	1.52246447	4.60E-09
FPGS	1279.943484	1948.51938	1.52234798	1.55E-10
ATP5G1	979.9976857	1491.316914	1.52175555	1.42E-09
MPDU1	1923.548322	2926.790978	1.52155833	8.31E-12
RIOK1	688.7396607	1047.922398	1.52150727	3.25E-08
C19orf52	210.0791856	319.6164649	1.52140948	0.0002239
SLC10A3	487.4782042	741.2524911	1.52058592	0.0001125
MED25	603.0215843	916.8922482	1.52049657	9.23E-06
FTSJ3	2982.408512	4534.305838	1.52035035	4.98E-13
MRPL32	964.0829679	1465.003751	1.51958265	1.90E-09
RABEPK	864.2204785	1312.795655	1.51905178	4.86E-09
CHID1	891.8490869	1354.686709	1.51896406	3.07E-08
RNPS1	1909.788324	2900.699586	1.51885921	8.44E-10
CLSPN	1728.320605	2624.512796	1.51853353	1.78E-11
RBM14	934.2741455	1418.296108	1.51807274	2.63E-09
SNAPC4	338.2594337	513.3922554	1.51774704	9.68E-06
CPSF3L	974.3814447	1478.736143	1.51761525	5.76E-07
ZNF496	440.6528776	668.3118909	1.51664025	1.86E-05
TSPAN9	1353.78878	2052.426868	1.51606137	1.47E-10
SMPD4	967.611838	1466.95067	1.51605284	3.58E-05
RNASEH2C	352.4389448	534.2138816	1.51576291	7.91E-06
TM4SF1	1596.279954	2418.807602	1.51527782	4.46E-11
TWISTNB	1205.348565	1826.169063	1.51505475	4.32E-10
SLC39A8	592.2581801	897.1208231	1.51474619	1.60E-07
TOMM22	1794.114276	2717.456143	1.51465053	2.05E-11
GEMIN6	376.5146127	570.1508411	1.51428609	5.91E-06
ANKRD33B	476.780134	721.5541721	1.51338976	8.65E-07
OSBPL6	642.1412839	971.6590359	1.5131546	7.92E-08
GFER	524.6728605	793.6640605	1.51268366	4.52E-07
PSMD12	2408.37937	3642.648238	1.51248939	3.31E-12
SERPINA3	243.6582942	368.3323862	1.51167596	0.0001084
ZFAND2B	357.0875062	539.6747388	1.5113235	8.01E-06
DUSP1	465.3344568	702.7148777	1.51012861	2.33E-05
KITLG	637.7994728	963.0807072	1.51000549	2.14E-07
COMMD5	475.8193317	718.4792126	1.50998323	1.15E-06
ULK3	533.6254081	805.5651509	1.50960794	4.23E-07
THUMPD2	226.7483877	342.2953385	1.50958224	0.0001877
PLS3	7051.999122	10644.1779	1.50938446	3.48E-14
MRPS18C	305.0730411	460.433189	1.50925558	2.86E-05
DCAF13	1899.08065	2865.602804	1.50894213	2.21E-11
MCM8	1577.473225	2379.580927	1.50847627	3.17E-09
FDX1L	311.4086682	469.6864657	1.50826394	2.75E-05
TUSC2	490.5634905	739.5698543	1.50759253	9.46E-07
AHSA1	3369.780083	5076.515138	1.50648262	8.33E-13
GPANK1	439.6026708	662.0297881	1.50597308	2.27E-06
SMYD4	497.9011836	749.5240253	1.50536703	9.67E-07

TMEM177	232.0991421	349.2523893	1.50475519	0.0001718
TYW3	1048.399385	1577.340904	1.50452292	2.44E-09
RELB	255.2415167	383.9314911	1.50418904	0.0001001
SLC37A4	558.3620054	839.6708093	1.50381079	2.17E-05
SNRPA1	247.8384896	372.6746693	1.50369973	0.0005319
TBC1D30	256.8151076	385.9492355	1.50282917	0.0001063
FAM96B	1153.467494	1733.15261	1.50255869	1.41E-07
COBRA1	1310.561097	1968.628279	1.50212629	1.07E-06
MYO1B	1974.582065	2965.989255	1.50208457	2.57E-11
AMD1	2604.754288	3912.078535	1.50189926	4.96E-12
PPP4C	1360.42013	2043.083919	1.50180365	7.07E-08
METTL13	1321.669788	1984.689942	1.50165341	4.85E-10
NCOA7	1559.347346	2341.397353	1.50152393	1.51E-10
MMACHC	267.847437	402.159563	1.50145011	8.65E-05
TIMELESS	3022.991054	4538.154883	1.50121347	2.34E-12
AP1M2	160.1720113	240.3984949	1.50087704	0.00144
BIRC5	3995.747328	5996.890645	1.50081828	5.90E-13
NET1	2088.007838	3132.956364	1.5004524	2.28E-11
POLE2	322.8921704	484.4572254	1.50036845	2.60E-05
RANBP1	1817.360521	2726.407358	1.50020171	5.96E-11
PNMA1	2201.102791	1467.229152	0.6665882	2.35E-10
CRTAP	10341.73239	6892.288771	0.66645399	7.40E-14
FAM171B	1169.011552	779.075011	0.6664391	0.0011284
LRP4	1109.400784	738.9586317	0.66608807	4.43E-08
SLC25A23	1641.431969	1093.100801	0.66594341	1.83E-09
SC4MOL	2622.563101	1745.516259	0.66557646	5.31E-11
MAN1C1	323.467134	215.2747312	0.66552273	0.0002756
EIF2C1	3259.577142	2169.226655	0.66549327	1.24E-11
TAOK1	10783.668	7174.160555	0.66528018	5.01E-07
DCAF5	2340.270666	1556.651462	0.66515873	1.07E-10
ABCC3	4957.520126	3297.221314	0.66509489	1.05E-12
C7orf73	3378.198931	2246.358422	0.66495741	9.91E-12
ICK	2813.494421	1870.82355	0.6649466	7.00E-10
SH3PXD2A	4081.837489	2714.100335	0.66492121	2.85E-12
KIAA1826	2184.158498	1452.085506	0.66482607	1.97E-10
SACS	6883.990104	4575.474784	0.66465447	1.55E-10
BCL2L11	996.4158676	662.1265595	0.66450825	2.94E-06
KLHL28	575.5030121	382.4022516	0.66446612	7.28E-06
KIAA0564	989.0197177	656.9889771	0.66428299	0.0001088
OSBPL1A	527.6721811	350.4795674	0.66419944	1.07E-05
6-Mar	5453.008692	3621.038412	0.66404413	5.68E-13
TM7SF2	300.3969706	199.4608701	0.66399095	0.0004004
NAB1	3369.711311	2237.41904	0.66397944	8.10E-12
MTRNR2L1	394.2787239	261.7369387	0.66383734	7.59E-05
LOC221710	2768.975826	1836.599907	0.6632777	2.76E-11
MEIS2	2878.276422	1908.372321	0.66302608	1.94E-11
ERO1LB	849.4281788	563.1866907	0.66301861	2.76E-07

LOC202781	208.502156	138.2231698	0.66293401	0.0024162
CHST3	9422.676221	6246.584843	0.66293107	5.57E-14
ICA1	196.409304	130.1994377	0.66289852	0.0027424
HNRNPA3	4956.491263	3285.485369	0.66286516	7.57E-13
VCL	23584.75219	15633.45599	0.66286285	6.38E-15
BCL2	347.5359246	230.354652	0.66282256	0.0001589
ATRN	4105.290052	2719.650864	0.66247472	1.85E-12
ZNF24	2530.53418	1676.368078	0.66245621	1.18E-10
ZNF780A	862.708783	571.1160178	0.66200325	2.65E-07
ZNF397	229.4306422	151.8682559	0.66193537	0.0015081
KIAA0776	2364.122823	1564.429758	0.66173794	6.29E-11
ZMYM2	1895.792484	1254.432357	0.66169286	3.97E-10
NPR3	690.4060946	456.8319458	0.66168585	1.27E-06
SLIT3	1367.957976	904.9274324	0.66151698	5.18E-09
PIK3R1	2547.382191	1685.043178	0.66148032	3.39E-11
MEIS3	365.0104788	241.4061838	0.66136782	0.0001084
APLP2	27660.33172	18292.79418	0.66133676	3.73E-15
LANCL1	5797.253012	3833.219252	0.66121303	3.35E-13
MACF1	21808.40144	14416.33906	0.6610452	9.22E-07
WDR6	5232.047054	3458.536304	0.66102928	4.29E-13
PHLDB1	1604.836347	1060.46539	0.66079348	1.17E-09
FIGN	651.5415657	430.5164164	0.66076585	1.92E-06
PAPSS1	3782.614516	2499.394314	0.6607584	2.37E-12
TNPO1	13448.40786	8882.618914	0.66049595	1.97E-09
IRGQ	940.4619114	621.1511556	0.66047455	1.08E-07
LOC100129	1619.226327	1069.126291	0.66026983	1.14E-09
ZFP36L1	2841.270179	1875.920987	0.66024027	9.20E-11
ZNF260	1851.945846	1222.644138	0.66019432	0.0008314
SYNJ2BP	2110.075936	1392.771145	0.66005736	1.28E-10
OBSL1	1966.667985	1297.777093	0.65988622	0.0017038
C5orf54	491.4589588	324.2230304	0.65971537	1.44E-05
C6orf170	400.0875283	263.9292936	0.65967888	6.43E-05
MTHFR	746.2355482	492.2709344	0.65967232	6.23E-07
ZNF805	632.7306863	417.3315221	0.65957212	2.07E-06
ANAPC13	3059.841135	2018.146599	0.65955927	7.29E-12
RBM41	763.8930618	503.782658	0.6594937	4.63E-07
ZNF37BP	433.7526028	286.0064108	0.65937682	3.10E-05
SLC39A10	1803.880476	1189.435283	0.65937588	2.49E-08
P4HTM	947.4550293	624.5754217	0.65921379	8.04E-08
ARL2BP	5421.208781	3572.796882	0.65904064	1.96E-13
KIFC2	780.5546749	514.2182342	0.65878567	0.0009501
LOC642852	781.395125	514.617067	0.65858751	3.82E-07
RUNX2	1460.028872	961.5302543	0.65856934	2.04E-09
HINT3	2238.731427	1474.044638	0.65842853	5.41E-11
ZMYM5	484.5793158	319.0359218	0.65837709	1.35E-05
ARHGEF40	1682.124548	1107.291018	0.65826934	5.89E-10
CELSR3	1828.74703	1203.658546	0.65818756	4.40E-08

FTH1	9520.918993	6266.233551	0.65815428	2.10E-14
COPB2	17738.9779	11670.01608	0.65787421	3.31E-15
ATP11B	7492.356277	4928.764943	0.6578391	1.16E-10
POMT2	1460.300524	960.5862908	0.65780041	1.60E-09
KIF5C	3343.542106	2199.355817	0.65779217	2.84E-12
B4GALT6	480.3234704	315.9208168	0.65772513	0.0001379
PDIA4	14636.67648	9626.405882	0.65769069	5.08E-15
PREX1	2959.261986	1945.456167	0.65741262	6.94E-12
CTIF	317.0833659	208.3459933	0.65707008	0.0001976
MUDENG	1809.33439	1188.857107	0.65706876	2.71E-10
TEP1	670.5037449	440.4965339	0.65696357	1.19E-06
ACTN4	56440.18187	37076.6486	0.65691937	5.43E-08
VPS8	5338.798199	3505.522429	0.65661265	4.97E-11
WWTR1	24887.4234	16339.23408	0.65652574	1.37E-15
LIG4	987.0293404	647.9858691	0.65650112	4.50E-08
BCL9	2448.173754	1607.106646	0.65645122	2.10E-11
KIAA1737	1011.245992	663.7714173	0.65638966	3.38E-08
SYT11	8714.799011	5719.186448	0.65626143	1.72E-14
IFT172	1029.140772	675.2807744	0.65615977	2.80E-08
TRIM52	251.5056168	165.0272038	0.65615713	0.0007181
NBPF1	595.9218687	391.0183594	0.65615709	2.44E-06
PION	871.5478557	571.5549963	0.65579302	1.02E-07
DCP1B	400.0703352	262.3434282	0.65574327	4.89E-05
WWC1	2796.818342	1831.596875	0.65488589	6.23E-12
ZNF547	220.474656	144.3589749	0.65476449	0.0013804
APPBP2	3844.256924	2516.982851	0.65473846	6.80E-13
RERE	3012.974545	1972.602409	0.65470265	3.47E-12
BMP1	1011.042401	661.6333217	0.6544071	5.27E-07
AKD1	212.1177038	138.7706669	0.65421539	0.0017484
TRIM47	2016.03802	1318.315504	0.65391401	0.0015791
RNF146	1476.863129	965.6223687	0.65383335	8.65E-10
RCOR3	1793.292443	1171.860946	0.65346896	6.05E-06
TSHZ1	802.4989815	524.3847949	0.65343983	1.95E-07
WDFY3	2210.74105	1444.521966	0.65341075	1.67E-08
COMMD6	1344.226883	878.2838953	0.65337474	1.94E-09
ITFG1	4100.576157	2679.147485	0.65335879	3.31E-13
ENTPD4	2107.769204	1377.056422	0.6533241	1.29E-06
COPG	9962.930252	6508.656889	0.65328741	5.87E-15
RAB4A	2369.504525	1547.87959	0.65325032	1.75E-11
YIPF6	3152.814377	2058.900147	0.65303564	1.88E-12
GPM6A	9669.059457	6314.210884	0.65303258	5.85E-15
LOC100507	3019.867943	1971.774063	0.65293387	2.64E-12
C9orf130	354.4912176	231.3930204	0.65274684	8.25E-05
MOBKL1A	1564.309535	1020.960233	0.6526587	0.0002146
FOXP1	3014.679516	1967.261903	0.65256087	2.30E-12
C17orf76	301.9086661	196.9900336	0.65248221	0.0002122
PQLC3	301.9258593	196.987667	0.65243722	0.000229

PON2	6383.188608	4164.352173	0.65239372	2.67E-14
GALNT7	3523.985113	2298.012471	0.65210618	7.61E-13
SLC41A2	748.146837	487.4826586	0.65158687	2.60E-07
CEP68	739.769253	481.8613046	0.65136703	3.14E-07
NT5DC2	5379.416431	3502.926508	0.65117221	1.81E-13
ARL1	5613.439313	3654.62252	0.65104873	1.57E-12
VPS13B	1686.21949	1097.801772	0.65104322	1.26E-06
TMF1	4296.924277	2797.030434	0.6509378	7.42E-07
ALDH6A1	661.0346905	429.9051939	0.65035194	7.38E-07
DNAL1	1157.039052	752.3252362	0.65021594	2.62E-06
CPNE2	1414.539159	919.6439329	0.65013678	8.65E-10
SLC23A2	2705.246759	1758.512343	0.65003769	3.75E-12
C2CD2	823.844254	535.5000521	0.65000156	1.01E-07
FARP1	835.6991286	543.1627303	0.6499501	0.0004259
BRD3	1192.938646	775.3109043	0.64991683	4.16E-09
ROM1	173.2772452	112.5825884	0.64972517	0.0032634
ACBD4	289.9292889	188.3503459	0.64964235	0.0002428
TIMP4	773.9508343	502.7112436	0.64953899	1.79E-07
RBM5	2896.164324	1880.836713	0.64942334	1.89E-12
BACE1	1937.821388	1258.035967	0.6492012	4.39E-11
IFT88	551.7237785	358.1705585	0.64918456	5.36E-05
RIMS3	538.7560899	349.6819016	0.6490542	3.45E-06
DDHD2	2383.680598	1546.713771	0.64887627	8.88E-12
ZNF555	264.5970962	171.6821931	0.64884383	0.0003964
TANK	1822.773883	1182.199751	0.64857181	2.00E-08
ZFYVE9	1422.255814	922.3389917	0.64850429	5.07E-09
TCEA2	930.2211795	602.9278169	0.64815533	0.0001305
PLCB1	1598.449852	1035.981162	0.64811615	8.60E-05
RRM2B	2162.545012	1401.566596	0.64810979	1.59E-11
CTBS	1499.447733	971.7227614	0.64805377	3.81E-10
HBP1	1663.420978	1077.775445	0.64792705	1.41E-10
FLNB	9653.425283	6250.377263	0.64747767	1.77E-15
BCKDHB	1335.663613	864.7520608	0.64743252	1.12E-09
LENG8	1854.075784	1200.356997	0.64741528	4.38E-05
TTC30A	502.4053224	325.2613988	0.64740835	5.09E-06
ATM	2599.92004	1682.319807	0.64706598	2.97E-12
GORAB	1170.398744	756.9365349	0.64673389	3.21E-09
LOC400657	307.2147183	198.6703039	0.64668225	0.0001385
IQSEC1	1476.928463	955.0262957	0.64663003	3.51E-10
MIB1	5717.499191	3696.777857	0.64657252	1.34E-14
WDR26	7679.390081	4965.209254	0.64656297	2.68E-07
CA13	299.8013752	193.7215313	0.64616625	0.0001746
PJA1	528.7945992	341.5637647	0.645929	3.00E-06
GABARAP	7055.715693	4557.315171	0.64590403	3.74E-15
ACVR2B	351.7436291	227.1805546	0.64586971	5.82E-05
FAM13B	3393.22577	2191.438237	0.64582742	3.32E-13
8-Sep	2665.889793	1721.610207	0.64579196	1.92E-12

VPS13A	3482.087589	2247.710685	0.64550665	4.79E-06
NAV2	6622.040164	4273.586054	0.64535792	4.77E-15
EHHADH	908.3807437	586.2030382	0.64532746	2.72E-08
C3orf62	279.2931142	180.2274759	0.64529867	0.0002665
MORF4L1	7138.77357	4604.523066	0.64500198	3.12E-15
ADCY9	823.413713	530.9477458	0.64481285	6.70E-08
SMARCE1	6747.531992	4350.769714	0.64479423	1.50E-14
PFKFB2	948.1476183	611.0860994	0.64450523	2.23E-08
EPC1	860.0402831	554.209529	0.6443995	4.22E-08
DSTN	9934.633837	6401.641396	0.64437618	7.65E-16
ZHX2	1095.193764	705.6530056	0.64431795	4.31E-09
GLG1	12587.66669	8109.658495	0.64425431	3.13E-16
SREBF2	8180.496783	5270.121051	0.64422995	1.55E-15
LRRCC1	3038.751745	1957.633373	0.64422287	3.39E-11
GLIS3	7779.547581	5011.522883	0.64419207	1.61E-15
SLFN5	1617.904468	1042.001349	0.6440438	3.74E-05
PANX2	329.8646565	212.4357413	0.6440088	0.0002443
PPP1R3E	177.4849497	114.2298128	0.64360281	0.0025352
ZBTB44	1606.651354	1033.99883	0.64357387	1.06E-10
TIA1	3257.907269	2096.595216	0.64354048	2.85E-13
WIPI1	1237.563839	796.3213403	0.6434588	1.13E-09
HS6ST1	1169.113999	752.1223127	0.64332675	1.99E-06
NUCKS1	35360.01141	22739.83491	0.64309467	3.07E-17
SIRT5	751.9343164	483.5415749	0.64306358	1.22E-07
KDELR2	15552.40393	9999.441605	0.64295151	1.42E-16
SLC35D1	2251.161977	1446.834672	0.64270572	1.33E-08
MAML2	2680.455855	1722.580117	0.64264446	7.20E-06
C10orf58	296.1101776	190.2713188	0.64256933	0.0001614
ATL1	631.5601273	405.7938522	0.64252608	7.52E-07
TBRG1	1014.889049	652.0779836	0.6425116	7.30E-09
CUX1	2034.859927	1307.372577	0.64248775	9.52E-12
MAN2A2	2115.932169	1358.61596	0.64208862	7.55E-12
FAM149B1	1003.353967	644.2029157	0.6420495	7.59E-09
PDE5A	538.615106	345.7738639	0.64196837	3.60E-06
GOLGA2	4020.433058	2580.500211	0.64184633	4.76E-14
SARM1	1417.334889	909.5175178	0.64170968	2.38E-10
KIAA2026	1421.195291	911.4951165	0.6413581	2.45E-10
DCAF8	4352.715194	2791.321774	0.64128289	2.39E-14
RAB2A	6039.733159	3872.540332	0.64117739	3.46E-15
KATNAL1	1818.634951	1165.814813	0.64103839	2.23E-11
TCEAL1	884.8594076	567.2127132	0.64102015	2.37E-08
RDX	8848.931182	5668.330064	0.64056663	4.13E-11
SNX13	3108.984933	1990.632204	0.64028364	2.91E-08
TNRC6C	547.2505875	350.3521165	0.64020419	1.62E-06
PDXDC2P	237.2848421	151.9013019	0.64016437	0.0004926
YIPF4	1181.964774	756.3559918	0.63991416	1.28E-09
SCP2	3680.114474	2354.513789	0.63979363	5.78E-14

KCTD4	1243.841009	795.6133463	0.63964232	7.46E-10
PTPN21	953.3745818	609.7645164	0.63958546	4.11E-07
GIT2	2391.304409	1529.080441	0.63943362	3.68E-08
IPP	891.99766	569.8157336	0.63880855	1.39E-08
PNMAL1	1484.403701	948.0668783	0.63868534	1.11E-10
LYPD1	7420.037733	4738.137304	0.63855973	6.45E-16
A2LD1	252.0152464	160.843051	0.63822746	0.0003549
VGLL4	6044.811549	3857.380205	0.63813076	1.65E-15
HMGN4	4526.770994	2888.284044	0.6380451	8.70E-15
TK2	964.0829679	614.8949992	0.63780299	7.34E-09
REEP2	952.278961	607.232321	0.63766222	6.24E-05
TMEM136	521.3399925	332.4379388	0.63766054	1.70E-06
KCTD11	499.38537	318.3916533	0.63756704	2.74E-06
HMGCS1	5048.675634	3218.796277	0.6375526	3.95E-15
TUBA1A	23155.8055	14762.30866	0.63752084	1.44E-17
ZNF223	331.2938248	211.1849833	0.63745524	5.41E-05
C9orf95	375.4334582	239.2964011	0.63738699	2.53E-05
N4BP2	1071.455794	682.8844602	0.63734264	4.79E-06
PCYT1B	1213.488171	773.3946645	0.63733185	6.45E-10
PSAP	93239.44413	59421.24761	0.63729732	3.84E-18
LEPR	561.781551	358.0171613	0.63728893	9.32E-07
ZNF638	4851.987801	3092.009273	0.6372665	1.67E-09
GSTZ1	432.6989574	275.7147657	0.63719767	8.60E-06
RNF103	1277.426995	813.6526083	0.63694646	4.21E-10
GABARAPL	2956.951816	1882.550029	0.63665225	1.57E-13
CUEDC1	2928.134032	1864.012797	0.63658725	9.61E-07
EIF4EBP2	5287.916467	3366.050868	0.63655523	2.77E-13
ZFYVE1	817.704629	520.0779242	0.63602174	3.13E-08
LRRC37A4	236.2449512	150.251711	0.63599967	0.0004426
ZNF652	843.0478494	536.1348545	0.63594831	0.0002029
FBXL20	686.2396538	436.3784731	0.63589807	1.41E-07
GPR124	290.4767434	184.6877438	0.63580906	0.0007763
ZYG11B	1989.784274	1264.782994	0.63563825	5.00E-12
IQCE	1293.916677	822.3536549	0.6355538	3.07E-10
LDB1	1302.462754	827.6092221	0.63541872	2.57E-10
RAVER2	1236.992314	785.9423899	0.63536562	3.98E-10
CTBP2	4688.27103	2977.123359	0.6350152	4.65E-15
CAMK2N1	411.1061032	261.0360444	0.63496028	9.45E-06
DYNC1LI2	8957.057781	5686.425952	0.63485422	5.44E-11
ZNF780B	749.1695347	475.5768352	0.63480536	5.23E-08
LRRC27	568.0518441	360.5800361	0.63476607	7.15E-07
IER5L	677.0594446	429.7187507	0.63468393	0.0003407
KBTBD6	967.8842018	614.1610588	0.63453981	5.08E-09
FBLN1	10808.92228	6858.385951	0.63451154	3.62E-11
SMO	1554.581159	986.3779029	0.63449753	1.79E-10
LONRF1	596.2939529	378.3148702	0.63444358	0.0010456
DACT1	226.9168808	143.9648751	0.63443881	0.0006015

SRSF8	1344.075583	852.2090685	0.63404847	1.67E-10
POU6F1	623.2066138	395.0750613	0.63393913	2.92E-07
HERC1	1798.236726	1139.89575	0.63389638	8.51E-12
CHD9	4662.210559	2953.915921	0.63358698	2.14E-06
ANO6	4240.391919	2686.076223	0.63344999	9.75E-10
KIAA0430	2538.409011	1607.83822	0.63340392	2.52E-12
GABRA3	549.7987352	348.1573951	0.63324517	8.10E-07
TCEAL3	829.5980403	524.9653379	0.63279481	1.76E-08
REEP3	4650.585361	2942.59064	0.63273554	4.35E-14
RSPH3	367.5001697	232.4903811	0.6326266	2.11E-05
EZR	54037.59957	34175.19395	0.63243361	1.40E-18
HOXA5	185.3391496	117.1915205	0.6323085	0.0013782
MARVELD1	3005.850047	1900.596391	0.63229914	5.70E-14
NCOA1	2489.63801	1572.821644	0.63174712	2.92E-13
KLHL29	510.9101358	322.6985239	0.63161504	1.33E-06
ZNF251	1237.556962	781.515168	0.63149834	2.43E-10
LAMC1	19743.66107	12460.44241	0.63111104	3.75E-18
WDR91	588.1914596	370.7442303	0.63031216	3.80E-07
HOXB7	965.2363337	608.1810175	0.63008509	2.89E-09
ATP9B	212.1211424	133.6142377	0.6298959	0.0006409
CREBZF	1594.527554	1004.225989	0.62979532	1.78E-11
GSTK1	2531.094677	1593.865126	0.62971375	1.54E-13
PHTF1	2185.522332	1376.228076	0.62970213	8.40E-09
STK36	1315.303213	827.9112835	0.62944519	1.08E-10
RHOBTB1	946.2053818	595.4256355	0.62927737	2.83E-09
NPAS3	463.6611456	291.6820241	0.62908447	2.63E-06
ZNF192	2139.712826	1345.707181	0.62891953	7.71E-06
LOC100505	289.7986209	182.2145407	0.62876262	8.57E-05
ADAMTS15	1061.3698	667.1295914	0.62855528	8.70E-10
NADSYN1	691.6316718	434.6675233	0.62846677	6.85E-08
LPIN1	3915.722566	2460.604251	0.62839085	3.27E-15
CDC42BPA	5506.03969	3458.194097	0.62807286	2.59E-08
LPP-AS2	213.7359969	134.2254602	0.62799651	0.0005584
TUSC3	1371.93873	861.4269327	0.62789024	5.87E-11
NIPSNAP3A	997.1868333	626.0999282	0.62786622	1.58E-09
DOK5	923.5451276	579.5810949	0.62756121	3.05E-09
STARD4	1166.542492	731.935489	0.62744006	2.86E-10
AKAP11	2813.466912	1765.202745	0.62741194	7.26E-11
PLA2G12A	1177.506049	738.5999444	0.62725788	1.43E-05
PJA2	11401.47773	7147.649117	0.6269055	6.92E-18
ARPP19	11731.1471	7352.865891	0.62678149	2.58E-11
APP	39933.33122	25028.60112	0.62675966	3.82E-19
HOXD10	164.8893453	103.3009988	0.6264868	0.0020692
ELOVL6	12961.25977	8115.065093	0.62610157	2.07E-11
STEAP2	389.5098104	243.7259896	0.62572491	3.70E-05
ATP13A2	1447.235842	905.4183037	0.62561904	0.0025188
ASAP3	3097.393409	1937.55752	0.62554453	1.30E-14

RHBDF1	374.0111672	233.8945363	0.62536779	0.001149
FHDC1	259.9107099	162.5233213	0.62530444	0.0001562
PTPLB	4204.72959	2629.225599	0.62530195	2.11E-11
CHM	1650.553722	1031.645978	0.62503023	7.49E-09
GNG7	421.6425576	263.5351938	0.62502039	4.54E-06
MKL2	1458.472475	911.530529	0.62498987	4.89E-11
PPARGC1A	1814.430685	1133.84725	0.62490524	1.98E-12
TMEM220	172.7882475	107.9736563	0.62489005	0.0014466
TASP1	519.2945971	324.496779	0.62487994	7.37E-07
CSRNP3	175.8941657	109.8639498	0.62460258	0.0014333
TMTC2	1209.673183	755.4379747	0.62449758	1.41E-10
AP2B1	19133.84581	11948.94045	0.62449236	1.38E-16
ANKRD6	515.6652948	322.0259425	0.62448636	7.23E-07
ATXN7L3B	3751.067048	2342.003842	0.6243567	2.07E-15
PAM	5037.337267	3144.862187	0.62431043	3.26E-10
NDFIP1	8085.453484	5045.102172	0.62397269	1.44E-17
CEP112	663.0491383	413.6051946	0.62379267	6.29E-08
BICD1	4157.191043	2592.939418	0.6237239	7.55E-16
DPYSL3	14741.03564	9191.320679	0.62351933	1.20E-18
OSBPL7	188.5447882	117.4959485	0.62317261	0.0009491
SPATA20	1445.559092	900.8141047	0.62315965	0.0002898
PDIA3	15091.99182	9399.142669	0.62279007	1.62E-13
KIAA0556	1127.450302	701.9597241	0.62260813	2.16E-10
LEPROT	4773.986379	2972.207718	0.62258404	2.54E-14
HOOK3	2134.995492	1327.899155	0.62196813	9.72E-06
CD109	12829.10908	7978.243626	0.62188602	2.05E-13
TTLL1	415.8268758	258.4731695	0.62158842	3.34E-06
SEC23A	6897.174426	4287.141468	0.62157939	8.01E-16
RAB27A	796.4907363	494.9305807	0.621389	7.15E-09
TNKS	3896.874574	2421.431836	0.62137792	1.31E-07
SLC40A1	224.6995536	139.5046072	0.62084951	0.0003332
NDRG3	1376.693889	854.441569	0.62064746	1.78E-11
NCRNA002	174.3618384	108.1884125	0.62048217	0.0011365
SOX12	1366.559755	847.9045644	0.62046651	5.35E-11
GBAS	2839.82868	1761.695907	0.62035288	9.45E-15
C6orf89	7219.62574	4478.03339	0.62025838	1.07E-17
SEC31B	356.4678404	221.1013753	0.62025616	1.37E-05
WDR52	574.3599622	356.2141731	0.62019325	1.91E-07
CD99L2	2999.318418	1859.628087	0.62001689	5.27E-15
NLGN4X	3308.00013	2050.58382	0.61988626	1.87E-15
GUCY1B3	707.7671739	438.6062405	0.61970413	2.21E-08
ZBTB26	269.3385007	166.7995125	0.61929324	8.38E-05
NEK11	740.2960757	458.4201777	0.61923897	1.24E-08
ANKS1B	1132.990893	701.5632577	0.6192135	1.27E-10
CRISPLD1	770.8449161	477.1910136	0.61904931	8.74E-09
DOCK9	1807.736729	1118.986818	0.61899877	6.97E-13
THBS3	1574.876225	974.4697129	0.61875956	3.28E-12

FAM120B	2353.644825	1456.052536	0.61863732	4.89E-14
RAPGEF4	344.4128132	212.9856049	0.61840209	1.37E-05
DPY19L1	2090.01197	1292.44132	0.61838943	2.39E-10
COA5	887.5347848	548.6235875	0.61814319	1.74E-09
ATG2B	1624.415465	1004.053745	0.61810157	1.91E-12
FBXO36	247.8419282	153.1827393	0.61806628	0.0001477
TOP2B	6954.027289	4297.24667	0.61795079	1.22E-09
NICN1	324.5414112	200.5299179	0.617887	2.19E-05
DIP2B	4850.754635	2996.399265	0.61771817	7.01E-17
VAMP2	1927.714763	1190.020559	0.61732191	2.77E-13
PEAK1	3180.368048	1962.768589	0.6171514	2.25E-09
AHNAK2	3024.623102	1866.552092	0.6171189	2.93E-15
MECP2	2895.895399	1786.300486	0.61683875	3.98E-15
DENND1B	741.7733849	457.4738477	0.61672993	1.23E-06
SLC22A5	363.285588	223.9757778	0.61652811	7.80E-06
FZD6	8610.027335	5306.751806	0.61634552	1.49E-18
MFI2-AS1	139.6775048	86.08534892	0.61631505	0.0030433
C14orf167	704.6990807	434.2686904	0.61624699	1.78E-08
DSC2	244.1576078	150.4051083	0.61601647	0.0001459
SHC3	255.678935	157.4872434	0.61595705	0.0001072
FAT1	8473.687561	5218.740836	0.615876	9.77E-08
HSP90B1	56642.91732	34876.24994	0.61572129	1.20E-20
PGPEP1	1171.948265	721.1624388	0.61535348	5.05E-11
IFT43	676.5154287	416.203482	0.61521654	2.20E-05
GCNT2	4877.977485	3000.682556	0.61514891	3.38E-17
PRKAR1A	19061.63256	11723.21112	0.61501611	6.08E-20
CRBN	1173.504663	721.6226306	0.6149295	4.44E-11
ACSS1	531.9933604	327.1257458	0.61490569	2.32E-07
KIF1B	6323.366069	3887.761903	0.61482474	3.48E-08
CETN2	2111.61514	1298.206605	0.61479319	6.58E-14
ZXDA	157.4931955	96.79940673	0.61462596	0.0017026
C2orf43	1169.868483	718.9983968	0.61459763	4.29E-11
SPECC1	3783.668161	2324.476665	0.61434475	2.37E-16
ARNT2	3096.450512	1902.085485	0.61427931	1.30E-15
MARCKS	19467.13475	11957.10574	0.61422011	4.28E-20
PHIP	4975.295977	3055.746672	0.6141839	1.69E-09
BDH2	282.4987527	173.4521353	0.61399257	5.00E-05
NBR1	8120.772661	4983.888052	0.61372092	7.91E-13
MANBA	984.019704	603.8222541	0.61362821	2.65E-10
DICER1	2856.480689	1752.475676	0.61350867	3.03E-10
GSTT1	1274.079661	781.177694	0.61313097	1.45E-11
SLC24A1	326.0187203	199.8880159	0.61311822	1.63E-05
TRAM1	16699.07099	10234.68832	0.61288968	8.30E-11
GPR137C	310.3034433	190.1509675	0.61279039	2.27E-05
CLSTN1	18753.53529	11490.98511	0.61273701	3.27E-20
EIF2C4	910.9254528	557.8414517	0.6123898	6.62E-10
SCRN3	1257.865782	770.1804215	0.61229142	4.90E-08

SCAMP1	4587.187114	2808.468966	0.61224208	3.26E-17
C11orf63	196.371479	120.2145872	0.61217947	0.0004615
IL6ST	13889.23481	8499.581775	0.61195465	2.60E-06
SYNPO	650.5360611	398.098128	0.61195397	2.42E-08
RCBTB1	1210.895321	740.9999559	0.61194386	2.42E-11
MLEC	20209.5457	12366.1681	0.61189738	1.98E-20
LOXL3	607.6426366	371.8109116	0.61189075	5.62E-08
PHF10	1024.423437	626.8338686	0.61188943	1.53E-10
UBL3	2037.587596	1245.792669	0.6114057	5.19E-14
ID4	1184.636712	723.6615882	0.61087216	2.64E-11
LOC339290	2955.636835	1805.314391	0.61080386	1.00E-15
EBF4	179.6335042	109.679873	0.61057581	0.0008195
DSTYK	2254.956334	1376.806253	0.61056892	1.66E-14
DYRK1B	425.4850551	259.7191944	0.61040733	0.0005463
SLC30A5	3573.761619	2181.061654	0.61029858	2.83E-15
NMNAT3	250.4519714	152.8452653	0.61027775	0.0001013
ACVR2A	700.3366377	427.370632	0.61023601	9.82E-09
CPA6	1505.170572	918.3436487	0.61012597	1.14E-07
LPP	4297.344502	2621.489814	0.61002552	1.48E-06
C5orf30	1165.295572	710.7834884	0.60995983	2.57E-11
LOC678655	460.0043342	280.538454	0.60986046	6.96E-07
PRRC1	4318.643649	2633.669301	0.60983714	3.19E-17
TCN2	1156.215795	704.9473782	0.60970226	3.07E-11
ACYP2	369.1390947	225.0117796	0.60955825	4.70E-06
TRIP11	2335.219785	1423.386445	0.60952997	1.97E-11
CDC42EP3	6945.993569	4233.007116	0.60941708	1.21E-17
TRAPPC9	971.9921859	592.275118	0.60934144	2.19E-10
C20orf194	905.7672619	551.0967905	0.6084309	4.19E-10
PPARD	2647.364321	1610.368048	0.60829106	1.94E-15
EEF2K	1839.025587	1118.498313	0.6082016	9.15E-14
NHLRC3	908.284462	552.4065409	0.60818671	4.22E-10
RWDD3	405.3144919	246.4398952	0.60802143	1.96E-06
ACSL3	22947.68669	13945.56444	0.60771112	8.00E-10
FN1	19024.74465	11557.59873	0.60750349	4.91E-20
ZNF417	381.7656467	231.8815251	0.60739233	2.91E-06
ARL3	2221.001702	1348.303102	0.60706982	8.73E-15
BBS10	1079.922782	655.5541424	0.60703798	5.87E-10
SORBS1	2344.568487	1423.237781	0.60703613	6.72E-09
HHIPL2	167.9643158	101.9582024	0.607023	0.0015664
CAPN1	1422.38577	863.3833181	0.60699659	1.14E-05
EDNRB	239.9499034	145.6120994	0.60684375	0.0001076
MFSD11	827.4632404	501.5501575	0.60612984	9.56E-10
DYNC1I1	2012.61646	1219.422881	0.60588935	1.99E-14
SLC7A11	11964.15961	7248.790807	0.60587547	2.82E-08
CYB5D2	1092.208198	661.449245	0.60560729	3.25E-11
ZNF621	1529.473189	926.05112	0.60547065	5.69E-13
EPHA4	2142.102085	1296.743458	0.60536025	9.35E-15

MAP4	17122.77673	10364.61173	0.60531139	5.02E-21
IQCK	730.2554964	441.9596815	0.6052124	1.69E-07
RAB5B	5704.135348	3451.871934	0.60515253	9.30E-19
SNX30	964.5375793	583.6755759	0.60513513	1.09E-10
LYRM5	714.0787306	432.107015	0.60512517	4.02E-09
DDX17	15293.32751	9251.645181	0.60494652	5.69E-21
NISCH	1544.550896	933.9922799	0.60470152	4.18E-13
CC2D2A	1046.928953	632.8115434	0.60444555	5.04E-11
ATP10D	2776.375415	1677.810012	0.60431669	4.41E-16
NR3C2	700.8840922	423.5263197	0.60427441	4.89E-09
CCDC92	1287.639506	777.8124203	0.6040607	3.89E-12
KIAA1598	3098.189869	1871.177504	0.60395831	1.73E-16
TRAF3IP1	1104.655941	667.1248584	0.60392094	2.34E-11
CXorf23	558.0697216	336.7400764	0.60340144	5.32E-08
ZFC3H1	1539.751746	929.0718202	0.60339066	9.06E-08
SPAG16	813.2459043	490.5906641	0.60325009	8.42E-10
ZDBF2	2225.375173	1341.459303	0.60280141	1.38E-08
PLXNA4	184.3164519	111.0863948	0.60269387	0.0004747
PPIC	1429.32802	861.0635123	0.60242541	7.38E-13
LBH	540.8977671	325.6578651	0.60206916	6.92E-08
FAM168A	1093.165562	657.8196889	0.60175669	1.72E-11
FAM63B	724.9735147	436.1613504	0.60162384	1.08E-06
C14orf19	160.0894841	96.31090201	0.60160667	0.0016191
SOX9	5006.611632	3011.316323	0.60146793	1.26E-18
SECISBP2L	3468.534621	2086.093548	0.60143368	6.45E-09
PLEKHM3	885.706735	532.4769854	0.60118882	2.11E-10
ZFP90	1548.115576	930.5468005	0.60108355	4.39E-07
TMEM2	7611.204304	4572.36196	0.60074093	5.20E-20
MRAS	1161.343038	697.4145174	0.60052413	6.43E-12
SOBP	341.8715428	205.2332549	0.60032272	4.48E-06
PRSS23	14195.89507	8519.825224	0.60016119	2.12E-21
ACAD10	844.3353219	506.5862354	0.59998228	3.71E-10
PCYOX1	5457.926179	3273.211392	0.59971705	3.20E-13
CDK19	2147.394383	1287.827655	0.59971641	3.18E-15
FSTL1	41290.68124	24739.43718	0.59915304	1.13E-22
HIPK3	2589.77974	1551.613017	0.59912934	2.06E-10
ZMYND11	3863.170963	2314.277058	0.59906152	6.18E-18
BPTF	4973.092404	2976.960581	0.59861357	1.20E-08
CLIC4	27478.94594	16446.36519	0.59850786	2.50E-18
MICAL1	1007.988062	603.142573	0.59836281	4.65E-05
ACADSB	2891.739986	1729.513588	0.59808752	1.36E-14
IFT122	2291.41026	1370.309394	0.5980201	1.10E-15
RABL3	1923.497454	1150.213341	0.59798017	2.64E-08
TMEM135	1200.861619	718.0780132	0.59796899	3.15E-12
RNF144B	143.8370684	85.99331056	0.59785222	0.0015286
ZNF532	1818.394247	1086.507171	0.59750913	9.90E-06
LOC100505	125.5461345	74.97482477	0.59718943	0.0030186

PRKAR2B	2772.694533	1655.341162	0.59701534	9.55E-17
CTNNB1	27954.21082	16686.72608	0.59693068	1.12E-22
RECK	977.5499701	583.4254072	0.59682413	3.57E-11
ASH1L	4152.663545	2477.482427	0.59660081	1.11E-10
LOC254128	186.926495	111.5135406	0.59656359	0.0003456
KCTD7	648.9934179	386.8955654	0.59614713	4.87E-09
TUBB2B	375.5091082	223.8506934	0.59612587	1.69E-06
PYROXD2	792.4652794	472.3696918	0.5960762	4.54E-10
SMARCD3	248.3721896	148.0239436	0.59597632	4.91E-05
MOSC2	543.8489468	324.1003126	0.59593811	2.07E-06
CPD	10676.07166	6360.37103	0.59575949	6.09E-10
LOC401321	326.5696135	194.54751	0.59573059	5.19E-06
ZNF84	1549.847344	922.154915	0.59499725	8.32E-14
AKAP9	4478.46907	2663.810296	0.59480377	1.31E-07
TMEM189	3540.801465	2105.947631	0.5947658	4.40E-18
HLCS	1138.331331	676.8666398	0.59461303	3.82E-12
AKT3	2904.779886	1727.198515	0.59460564	3.02E-11
HIPK2	10049.69581	5974.464307	0.59449205	1.75E-21
ZBTB8A	671.9913699	399.4409243	0.59441377	2.75E-09
GNG2	1551.355601	922.121869	0.59439749	7.55E-11
UPRT	1306.281893	776.3516392	0.59432167	6.25E-13
C12orf23	6335.447893	3764.217454	0.59415175	6.07E-20
EIF2AK3	2439.28654	1448.696652	0.59390179	2.45E-16
TSPYL1	4382.286039	2602.480557	0.59386369	5.43E-19
ROBO1	9442.838603	5607.49593	0.59383583	4.78E-16
APBA1	391.179683	232.2779914	0.59378849	8.91E-07
RASGRP1	391.6961898	232.5234271	0.59363209	8.29E-07
DST	17638.85277	10466.05092	0.59335213	2.19E-07
VPS41	3943.633855	2339.785541	0.59330699	1.02E-18
SENP7	727.0739283	431.3400286	0.5932547	1.82E-07
MYO5C	655.3187292	388.7551794	0.59323069	3.48E-09
EGF	1122.970945	665.6050849	0.59271799	5.87E-12
MAPK8IP1	1317.402915	780.8095406	0.59268849	0.0001027
GRAMD4	1175.938624	696.9189131	0.59264905	5.93E-10
IDH1	6497.089625	3850.231978	0.59260872	1.52E-20
CSAD	234.7607648	139.0821945	0.59244224	5.58E-05
C5	712.2644354	421.9404542	0.59239298	0.0010914
GNS	12410.39636	7350.968498	0.59232343	2.42E-19
OFD1	1269.397425	751.8060521	0.59225428	7.89E-09
ACCN2	378.6494127	224.1268085	0.59191115	1.10E-06
MTCP1NB	321.8247704	190.486075	0.59189376	4.87E-06
ZNF827	664.708695	393.3051191	0.59169546	2.35E-09
LRP11	2375.296136	1405.410909	0.59167819	2.08E-16
AHSA2	558.795985	330.5995382	0.59162833	2.09E-08
CBX5	9642.791244	5701.581431	0.59127915	6.52E-15
NAT8L	471.7285408	278.9195426	0.59127129	0.0011548
C1orf173	187.4189314	110.7512874	0.59092903	0.0002583

ABHD4	3953.640048	2334.834402	0.5905531	5.42E-19
CNOT6L	800.4233501	472.4924097	0.59030313	0.0019886
SEC31A	10558.17542	6230.922098	0.59015141	3.22E-20
SAMD15	142.2669162	83.94961987	0.59008533	0.0011678
DOCK1	5773.862344	3406.17198	0.58992954	1.60E-20
ARHGEF17	666.5745697	392.9983246	0.58957893	6.78E-05
EEA1	2794.997169	1647.824781	0.58956224	1.06E-09
ZNF404	184.2923814	108.6438712	0.58951906	0.0002856
PLCD1	194.2951358	114.5011948	0.58931581	0.0020879
LCORL	1140.349218	671.9768596	0.58927287	1.92E-12
ZNF594	631.1158318	371.8156446	0.5891401	3.20E-09
JDP2	359.7594447	211.8811446	0.58895228	5.54E-06
RNF19A	2841.212434	1673.250607	0.58892133	1.42E-17
DAPK2	811.2555269	477.6181594	0.58873948	1.46E-10
PSAT1	8099.474818	4767.950205	0.588674	1.18E-21
EVL	411.2883508	242.1047116	0.58864957	8.52E-05
CDKN1B	3002.927088	1767.057626	0.58844507	6.94E-18
CCDC89	119.2242619	70.14876996	0.58837663	0.003033
SYNGR1	1162.430358	683.882683	0.58832142	8.38E-05
CEBPD	384.9128285	226.4442477	0.58830008	7.91E-07
CRYGS	121.2834119	71.3429021	0.58823297	0.0023254
KRCC1	1850.502924	1088.298326	0.58810949	1.61E-07
C2orf63	343.3797996	201.9364396	0.5880848	1.88E-06
TBC1D19	268.3364347	157.6075947	0.58735071	1.84E-05
ERLEC1	4629.026181	2718.567617	0.58728716	6.52E-20
DDR2	5615.302461	3297.589468	0.58725055	4.88E-11
ZNF772	1481.673306	869.8589638	0.58707878	3.16E-14
CPE	541.311115	317.7568509	0.58701335	1.37E-08
EFHD2	6926.145405	4064.812829	0.58687951	2.32E-05
ZNF323	222.1238968	130.3481019	0.58682611	7.33E-05
ALCAM	18283.51872	10723.02062	0.58648561	1.36E-23
DKK3	8452.078105	4955.328275	0.5862852	4.45E-22
CLN5	1458.138215	854.8073559	0.58623205	3.57E-14
ASS1	1028.22811	602.7413736	0.58619422	5.24E-12
CCNL2	2695.598184	1579.906145	0.58610595	1.29E-17
AP3B2	674.1124153	394.9570766	0.58589201	9.22E-10
SLC22A23	3118.098384	1826.839278	0.5858825	2.15E-18
KBTBD3	163.2779295	95.64068711	0.58575392	0.0004854
PXDN	6832.152312	3998.796319	0.58529086	1.52E-21
ZFHX4	3500.148134	2047.938287	0.58510046	6.10E-05
AXIN2	173.793752	101.6844539	0.58508694	0.0003341
ATAD2B	551.2072717	322.1486603	0.58444196	2.71E-07
SASH1	2630.513583	1536.835158	0.58423388	1.07E-17
MOAP1	876.871101	512.143864	0.58405832	2.84E-11
TMED10	14091.20249	8227.251129	0.58385728	1.59E-23
WLS	10171.14012	5937.642376	0.58377353	7.20E-23
VEZF1	7468.528191	4359.876693	0.58376652	4.60E-22

ZNF32	433.2017097	252.8848614	0.58375776	1.37E-07
TNIK	1902.84062	1110.373077	0.58353446	6.33E-16
KDELR3	1854.985719	1082.384377	0.58350011	8.04E-16
CSRP1	11038.47269	6438.675716	0.58329408	4.07E-23
TMEM221	170.1850816	99.21125086	0.58296091	0.0003627
ACSS3	2093.837274	1220.484829	0.58289383	1.49E-16
NR1D2	2706.452416	1577.489568	0.58286248	6.16E-18
SCGB3A2	197.5179675	115.1171504	0.58281863	0.0014099
ZBTB1	2287.929531	1333.065051	0.58265127	1.41E-12
IGF2R	10581.83359	6161.132016	0.58223671	3.52E-23
ITGB8	12597.71557	7334.739324	0.58222773	8.41E-18
C14orf159	1028.596756	598.6870382	0.58204251	2.38E-12
C10orf118	1382.76818	804.7391721	0.58197692	3.11E-08
OSBPL10	6569.615789	3823.168395	0.58194703	6.30E-22
MLL3	2842.742034	1653.417823	0.58162781	3.39E-09
LYSMD3	3071.018599	1785.710477	0.58147172	1.02E-18
MYL5	176.4484974	102.569425	0.58129951	0.0002664
TRIM66	525.0071198	305.1760795	0.58127989	1.29E-08
PABPC4L	207.9512629	120.8210766	0.5810067	0.0001013
FLJ31306	980.1187497	569.1384191	0.58068313	1.11E-08
C11orf54	656.8991973	381.3686163	0.58055881	8.20E-10
DLG5	5784.803134	3357.649516	0.58042589	3.49E-21
ZNF254	441.0387164	255.9645539	0.58036754	9.69E-08
ZXDB	378.1019581	219.3927921	0.5802477	5.17E-07
MTERFD3	604.3819799	350.5692392	0.58004582	2.08E-09
CCNG2	1581.8563	917.1400504	0.57978721	3.79E-15
PLXNA2	867.9632557	503.2327943	0.57978583	1.70E-11
PPM1H	290.3976549	168.3546985	0.57973849	5.60E-06
PDE4DIP	4032.967479	2338.027346	0.57972879	2.29E-11
AMOTL1	3920.990794	2272.983113	0.57969611	3.94E-20
DHFRL1	700.3572695	405.9708293	0.57966248	3.42E-10
PLD1	561.2237806	325.2590323	0.57955319	5.57E-09
BNIP3L	3961.422036	2295.171115	0.57938061	3.47E-20
GAB1	919.7885959	532.6586956	0.57910992	7.19E-12
CEBPA	448.1143615	259.5068048	0.57910843	0.0024493
KLF13	2128.324894	1232.452011	0.57907137	5.40E-17
SCD5	6135.680939	3552.506187	0.57899135	5.11E-22
VWDE	435.7979983	252.3019518	0.57894243	9.59E-08
DMXL1	2480.482583	1435.846865	0.57885787	1.09E-08
BMPR2	4231.039778	2449.123207	0.57884665	3.34E-16
VAT1	5076.224442	2938.208211	0.57881763	2.85E-21
SLC7A5	20704.06896	11977.61102	0.57851483	5.44E-07
KIAA0895	887.7693237	513.0288351	0.57788529	1.04E-11
WDR63	147.4973184	85.2310573	0.57784818	0.0006531
TXNDC15	2642.661453	1525.80484	0.57737431	2.51E-18
GSTM4	1134.171768	654.8249351	0.57735958	2.96E-13
KIAA1109	2945.042635	1700.09242	0.5772726	5.08E-08

NHSL1	3209.25662	1851.955942	0.57706695	2.00E-19
SOX2	18722.1728	10801.91988	0.57695867	6.86E-25
TBC1D5	3020.850089	1741.778099	0.57658541	8.80E-13
EVI5	1317.916695	759.8368837	0.57654394	2.89E-14
DNALI1	198.4650153	114.4138895	0.57649399	9.75E-05
C8orf83	1533.133439	883.747119	0.57643196	2.95E-15
C20orf177	546.6962557	315.0358457	0.57625389	5.00E-09
OSTM1	7519.48486	4332.83897	0.57621487	5.60E-23
BRWD1	1841.363978	1059.972152	0.5756451	2.09E-16
ZNF708	212.6548424	122.2889573	0.57505842	5.75E-05
GALC	260.3893917	149.7325268	0.57503313	1.13E-05
RAB39B	490.9493293	282.2800832	0.57496786	1.65E-08
ZNF322	190.0324132	109.1937349	0.57460584	0.0001214
ZNF287	615.304273	353.5285804	0.57455896	9.59E-10
ZNF512	1962.179024	1126.307289	0.57400842	6.00E-17
ALK	134.9154685	77.38430236	0.5735762	0.0009978
NUAK1	7438.22622	4265.614215	0.57347197	2.17E-23
LAMP2	29670.21103	17012.95248	0.5734018	2.30E-23
PCDH10	23654.01557	13562.18044	0.57335637	8.22E-26
ZNF449	428.415603	245.6186496	0.57331864	6.61E-08
DAP	12466.08886	7146.492764	0.57327465	9.45E-25
KLHDC2	1722.907243	987.6923863	0.57327078	2.89E-16
SIM1	573.32351	328.6478858	0.57323288	2.20E-09
FBXO32	22844.47817	13086.99771	0.57287357	7.65E-26
FAM122A	624.7079934	357.8024051	0.57275144	2.25E-08
JMY	2326.979746	1332.276851	0.57253479	3.58E-18
LMBRD1	2403.548561	1376.048733	0.57250715	2.43E-18
NUDT16	4002.875977	2291.673743	0.57250681	4.22E-21
HTRA1	10200.60093	5837.697185	0.57228954	1.98E-24
ABCA1	15302.91834	8755.300978	0.57213276	3.39E-16
ZNF33B	537.0930946	307.2811291	0.57211893	4.18E-09
KAT2B	616.7300027	352.7922736	0.57203683	0.0012788
ABCC4	3304.429996	1889.820974	0.57190528	1.61E-16
PPP1R9A	1316.75989	752.1128466	0.57118451	1.16E-14
KREMEN1	779.3497296	444.9284889	0.57089709	2.26E-11
HAS2	418.9568645	239.1784164	0.57089032	6.67E-08
RNF130	2113.777449	1206.594307	0.57082372	1.00E-17
ZFX	1386.156066	790.6150475	0.5703651	3.77E-15
PFN2	29395.47351	16761.13684	0.57019448	1.50E-26
NPHP3	216.3804264	123.3603717	0.57010874	3.46E-05
C7orf41	855.2679309	487.3575743	0.56983029	5.54E-12
ZNF83	478.9355658	272.8474629	0.56969555	1.36E-08
TPBG	5675.400091	3233.22483	0.56969108	7.01E-23
C5orf24	3972.16552	2262.382307	0.56955892	9.80E-15
ARSD	790.8091613	450.2383153	0.56933877	1.57E-11
MLL5	3484.060773	1983.316466	0.56925427	8.26E-07
NPHP3-ACA	105.5509417	60.07661414	0.56917175	0.002878

MMP14	3602.911527	2050.399657	0.5690952	7.81E-21
PBXIP1	1918.617081	1091.776852	0.56904364	2.61E-09
SYNGAP1	885.28651	503.7189325	0.56898973	3.08E-12
PARVA	4238.398103	2407.874055	0.56810946	2.99E-17
TRAPPC2	271.9622984	154.4972227	0.56808324	4.55E-06
SCARB1	2982.376852	1693.829164	0.56794605	5.09E-20
ALDH3A2	9780.777532	5552.943899	0.56774054	5.80E-25
C5orf41	973.9619315	552.5056789	0.56727646	4.85E-13
UBQLN2	1580.137575	895.8653321	0.56695401	3.21E-16
CPEB2	1008.036915	571.4889043	0.56693252	3.11E-13
FAM125B	544.6130352	308.6239255	0.56668479	2.08E-09
WDR5B	657.295352	372.4268672	0.56660505	1.46E-10
IDS	12070.98629	6838.8341	0.56655139	1.06E-25
CALU	43486.86394	24632.76845	0.56644159	5.98E-17
SLC17A5	864.8504602	489.8614568	0.56641174	2.88E-12
LOC100506	267.7786643	151.6582328	0.56635667	4.73E-06
EPHB3	2617.331275	1482.117897	0.56627066	2.25E-06
NBEA	922.3573755	522.0059967	0.56594766	2.91E-08
LRRC8D	10637.8364	6020.176008	0.56592109	1.62E-19
MAN1A2	3876.197108	2193.269538	0.56583024	8.17E-20
KLC4	753.2252276	426.0254691	0.5656017	1.29E-05
CYFIP2	2145.965215	1213.619816	0.56553564	2.30E-18
MYOM1	113.9285256	64.41179769	0.56537024	0.001748
MOXD1	9103.530978	5144.438507	0.56510364	3.91E-25
SLC33A1	3116.3563	1760.985546	0.56507837	1.41E-20
LOC253039	904.6173348	510.9568315	0.56483201	1.09E-12
FADS2	10210.08161	5764.010897	0.56454112	1.57E-25
C17orf28	281.4623005	158.8913986	0.56452107	2.89E-06
FLJ90757	911.720489	514.650113	0.56448234	9.64E-13
EFHA2	164.8377658	93.01645333	0.56429091	0.0001876
ANXA2P1	2202.693575	1242.9159	0.56427091	1.44E-18
LOC100506	3126.797185	1764.072338	0.56417869	3.44E-11
ZNF605	702.36484	395.9907118	0.56379632	2.51E-06
RHOQ	2049.673571	1155.556214	0.56377573	3.11E-18
CAB39L	354.8908108	200.0720926	0.56375676	2.54E-07
PTPRU	160.7194658	90.54325029	0.56336207	0.0002225
APH1B	629.4769069	354.5952617	0.56331735	2.11E-10
HMGN3	5585.296213	3145.761272	0.56322192	1.22E-23
FRG1B	286.0971073	161.1215326	0.56317079	5.41E-06
MAP3K12	1144.332699	644.1698697	0.56292184	2.56E-14
LOC283547	439.4857573	247.3885917	0.56290468	2.03E-08
PHF2	2110.674258	1187.629928	0.56267798	2.02E-18
DENND5B	2111.942522	1188.189258	0.56260492	5.23E-09
ZNF525	129.6816277	72.92876753	0.56236777	0.000802
MYO5A	3278.650069	1843.228949	0.56219142	3.38E-16
FAM200B	1135.611252	638.2771336	0.56205601	2.50E-14
SETBP1	660.8661975	371.3908654	0.56197588	8.76E-05

C10orf32	561.8468851	315.7367401	0.56196225	8.18E-10
SGCB	4686.133503	2632.741903	0.56181539	4.15E-23
SLC5A3	6339.65631	3559.763019	0.56150726	6.83E-10
ARL6IP5	11486.32915	6447.409809	0.5613116	2.57E-26
DOPEY1	1074.141487	602.871191	0.56125864	7.72E-14
MCEE	205.8646038	115.5419296	0.56125204	3.91E-05
COL5A2	30821.5966	17297.75538	0.56122191	5.80E-28
TCF4	3742.355917	2099.77878	0.56108474	4.21E-22
PTPN14	3939.481168	2210.348357	0.56107601	2.24E-22
PRR15	285.6837595	160.234195	0.56087961	2.03E-06
CHRNA9	169.6307498	95.02946458	0.56021367	0.0001446
BTBD19	170.7222202	95.63832057	0.56019843	0.000705
POU3F2	3382.262213	1892.669345	0.55958682	1.28E-21
MTMR10	1913.163879	1070.027828	0.55929753	2.92E-16
ELMOD2	2115.185986	1182.992683	0.55928542	3.38E-12
KLF12	944.5458251	528.2645197	0.55927887	2.51E-13
FAM160B1	1381.459364	772.5332733	0.55921534	6.55E-16
ZNF75D	580.1240644	324.4094737	0.55920706	3.39E-10
PRMT2	4625.08254	2585.732199	0.55906725	2.17E-23
TRAM1L1	225.2745172	125.8312082	0.55856832	1.49E-05
SPATA7	181.6617065	101.318667	0.55773266	7.62E-05
EPB49	686.818056	382.803451	0.55735787	2.77E-11
LOC401320	139.6671889	77.81144818	0.55712046	0.0004734
PTAR1	6082.99594	3384.753245	0.55642865	9.42E-12
PRDM1	655.7423929	364.8279144	0.55635859	4.01E-11
HSPC159	628.4507705	349.5568173	0.55621989	9.94E-11
CLMN	909.9474573	505.2222257	0.55522132	2.05E-13
LRRC17	286.1039846	158.8017268	0.55504899	1.30E-06
ANTXR1	4531.788201	2515.283734	0.55503118	5.02E-14
UBXN4	10342.6588	5737.482978	0.55473966	4.94E-27
ARHGEF12	5698.97858	3160.673682	0.55460354	1.40E-08
ZNF503	841.8593855	466.658666	0.55431902	8.45E-13
IFT81	2094.818709	1161.170468	0.55430595	2.70E-19
TMEM56	922.8841982	511.3202518	0.55404595	8.59E-09
ARHGEF25	567.60411	314.4529361	0.55400046	3.15E-10
B3GNT9	1427.361713	790.5465889	0.55385161	1.29E-16
TSNAXIP1	103.9807894	57.57273165	0.55368623	0.0021017
ZNF767	225.7875854	124.9462371	0.55337957	1.08E-05
GOLGA8B	670.9377245	370.961353	0.55289983	2.02E-11
ARHGAP31	912.8883211	504.6015371	0.55275276	3.30E-07
ACACB	829.6530585	458.4768036	0.55261268	8.42E-13
ZNF362	1290.90704	713.3133173	0.55256753	5.58E-16
GOLGA2B	258.2889781	142.6810711	0.55240867	3.14E-06
NDRG2	158.6087363	87.58390907	0.55220104	0.0005406
ZDHHC1	223.2531922	123.2352873	0.55199787	0.000165
DBP	102.420953	56.5343633	0.55198045	0.0022226
MGC39372	140.1974502	77.32294346	0.55152889	0.0003799

WDR49	316.1363181	174.3394729	0.5514693	3.40E-07
ZNF546	255.1555509	140.6987393	0.55142339	3.03E-06
TMEM8B	244.2676441	134.652606	0.55125028	0.0001965
CD9	7318.977888	4033.857688	0.55115041	2.48E-26
ITGAV	13573.17615	7473.108706	0.55057922	9.29E-14
FADS1	13014.26871	7165.218806	0.55056638	1.53E-25
CEBPG	7708.253871	4239.690419	0.55001956	8.86E-27
PMEPA1	32923.82854	18099.56817	0.54974069	7.02E-30
NCRNA002	823.8614472	452.8342362	0.54964853	5.12E-13
SMARCA2	2227.28575	1223.604667	0.54937031	2.70E-20
PALLD	20802.44632	11416.55787	0.54880843	1.22E-18
MSRB3	3990.521077	2189.647082	0.54871207	5.62E-24
CASC4	8320.435033	4564.250923	0.54855917	2.84E-27
CASD1	2173.067712	1191.976945	0.54852269	3.37E-20
CYP1B1	137.5358276	75.43265003	0.54845818	0.0003341
MRC2	6208.26544	3399.726928	0.54761301	1.82E-05
PLXND1	13305.62005	7285.652797	0.54756207	1.58E-09
C17orf91	495.1776656	270.9524364	0.54718226	1.04E-09
ZFP30	671.5505131	367.3860562	0.54707137	1.10E-11
ST3GAL1	4177.75776	2284.275347	0.54677065	1.85E-24
PDK3	1541.989705	843.0596628	0.54673495	6.44E-18
LOC100130	100.3239781	54.82578006	0.5464873	0.0020091
SAP30L	1323.612024	723.3217477	0.54647565	1.24E-16
DBC1	677.8895788	370.3760769	0.54636638	9.24E-12
TPP1	4293.131936	2344.731947	0.54615884	9.14E-25
DENND2A	2028.613705	1107.836148	0.54610503	7.40E-20
NDRG4	1021.89936	558.0585744	0.54609935	8.24E-15
NCAM1	1455.476593	794.4522602	0.54583651	1.88E-17
NELF	3052.054996	1665.88999	0.54582568	2.80E-10
MAP2K5	574.5490871	313.4452472	0.54554999	1.03E-10
MAGEF1	10785.35494	5882.844909	0.5454475	1.47E-28
TOB1	5011.707928	2733.50834	0.54542451	1.33E-25
MFSD6	3002.827368	1636.570326	0.54500979	8.68E-23
DNAJC10	11010.87645	5998.403404	0.54477075	9.47E-29
VIM	247020.4391	134528.9367	0.5446065	8.69E-32
GTF2I	2580.906281	1405.226832	0.54447031	5.67E-16
COL12A1	4481.757236	2440.094153	0.54445032	2.58E-11
LCTL	573.9431758	312.4682378	0.54442365	9.13E-11
DYNC2H1	604.2375573	328.9216344	0.54435814	9.35E-09
ZNF436	9702.078068	5278.680533	0.54407731	5.94E-17
ZNF514	351.7436291	191.371046	0.544064	6.23E-08
PNRC1	1400.886471	762.1236435	0.54402956	2.62E-17
DLG1	16079.45514	8746.415855	0.54394977	4.77E-19
CRYL1	502.6219563	273.366647	0.54388123	5.40E-10
TCTN1	2039.336557	1108.702273	0.54365831	2.81E-20
FMNL2	6663.415727	3621.283848	0.54345759	1.86E-19
MAP9	3024.04885	1642.538535	0.54315873	5.82E-18

CASC2	277.2374028	150.556139	0.54305854	8.87E-07
AHNAK	7006.595374	3802.349135	0.54268142	1.44E-27
SUMF1	1154.184154	626.0692488	0.54243445	5.55E-16
RUFY3	1023.276949	554.9458359	0.54232223	3.99E-15
DDIT4	3928.265168	2130.073086	0.54224269	7.76E-24
ZNF302	1644.163077	891.4121638	0.54216773	2.67E-08
DISP1	1311.918765	710.8967401	0.54187558	1.88E-09
TWSG1	12766.71563	6916.121546	0.54173068	1.37E-29
MR1	1273.752991	689.4450447	0.5412706	6.96E-17
TUG1	9489.939732	5126.134963	0.54016518	7.74E-17
PRR5L	1897.300029	1024.471805	0.53996299	4.84E-20
C15orf17	1374.696635	741.917973	0.53969578	1.40E-17
SLC35E2B	1273.332766	687.2101776	0.5396941	6.34E-17
GATM	568.6749486	306.6392271	0.53921705	5.31E-11
CMTM4	3039.680888	1638.861819	0.53915588	1.33E-23
CCDC40	162.795809	87.73257326	0.53891174	8.25E-05
LOC155060	193.8336471	104.400726	0.53860992	4.93E-05
SFT2D3	634.7898363	341.8375132	0.53850502	8.10E-12
1-Mar	1200.303849	646.2843855	0.53843399	1.04E-16
MCC	11649.65321	6270.977033	0.53829731	6.54E-30
AGXT2L2	289.4402912	155.7149347	0.53798638	0.0001124
YPEL3	231.6857943	124.6394426	0.53796756	0.002829
JUB	1111.666252	597.7997006	0.53775106	4.91E-16
SYNE1	4100.810696	2204.299857	0.53752783	4.38E-18
TMEM50B	1965.705167	1055.518984	0.53696709	1.17E-20
ZNF471	255.1039714	136.9134194	0.53669654	6.65E-05
MYO16	153.3061228	82.26934954	0.53663447	0.0001091
FNDC4	1392.081784	746.4395998	0.53620384	4.84E-18
VMAC	244.1851169	130.928645	0.53618602	1.99E-06
SPEF2	274.1005369	146.9548958	0.53613502	5.99E-07
C14orf101	599.0346642	321.1409714	0.53609748	1.47E-11
PSD4	1821.612216	976.3906858	0.53600359	1.01E-06
IL13RA1	3959.242534	2122.122546	0.53599206	1.23E-25
TGFB2	3923.063698	2101.30092	0.53562753	1.30E-25
YPEL1	267.2690348	143.1082169	0.5354463	7.62E-07
NMNAT2	1516.223533	811.2454973	0.53504347	8.08E-19
MUC20	553.3902125	296.0502537	0.53497559	4.11E-11
TMCO3	4661.172683	2493.105112	0.5348665	1.12E-26
ABI3BP	175.8632179	94.05245514	0.53480458	3.54E-05
SSBP3	496.7581337	265.5529381	0.5345719	2.19E-10
SGCG	152.2834251	81.38201193	0.53441149	0.000118
ACACA	15045.98489	8038.825311	0.53428376	2.58E-31
KIAA1407	290.8694595	155.3491478	0.53408546	2.75E-07
SH3BP2	992.527956	529.8527517	0.53384164	1.62E-15
HSPG2	2866.353367	1529.250319	0.53351772	0.0019561
LOC146880	311.4464932	166.0655721	0.53320739	1.09E-07
TNS3	11882.83824	6328.228857	0.53255197	5.90E-31

SOS2	2020.360623	1075.727021	0.53244307	2.37E-21
STXBP6	860.0884239	457.7782757	0.5322456	1.74E-14
LOC100506	878.4584464	467.0881783	0.53171346	1.12E-14
IL18BP	217.3996855	115.5419296	0.53147239	5.01E-06
BAZ2B	1482.124479	787.065697	0.53103886	5.56E-19
BIVM	652.1405997	345.7148715	0.53012322	1.68E-12
PDIA5	1445.063929	766.0364143	0.53010555	6.71E-19
KIAA1370	752.761012	398.983099	0.53002625	2.58E-07
C14orf28	99.75933038	52.87176119	0.52999314	0.0013084
DNHD1	349.2814471	185.1148896	0.52998775	1.97E-08
4-Sep	1058.016301	560.6497622	0.52990654	2.78E-16
ADARB1	3322.341256	1759.312376	0.52953994	2.26E-25
DPY19L3	3547.444554	1877.454959	0.52924152	1.74E-17
GALNT2	6938.233515	3671.590368	0.5291823	1.53E-29
DTNA	1290.446263	682.4573144	0.52885373	4.46E-15
RBM43	733.9673259	388.120377	0.5287979	1.55E-13
ZNF497	87.16372601	46.03506168	0.52814472	0.0022904
UST	1878.524248	991.8080806	0.52797193	3.01E-21
DKFZP586I	284.1204845	149.947283	0.52775949	2.15E-07
ZNF704	365.8268585	192.959278	0.52746066	0.0005426
UBE2H	12650.25887	6668.881704	0.52717354	4.32E-32
CACNG4	5138.564894	2708.476615	0.52708814	2.19E-28
CDO1	487.2203067	256.5757764	0.52661142	1.34E-10
LACC1	183.734611	96.73804782	0.52650966	1.71E-05
KLHL5	6633.390271	3492.273809	0.52646892	6.72E-18
DCLK2	777.9762912	409.5768056	0.52646438	2.19E-08
ECE1	19248.91928	10127.48875	0.52613285	3.13E-33
PPP1CB	36554.00916	19231.62198	0.52611526	2.94E-34
GOLGB1	7971.930125	4193.140906	0.52598817	9.62E-09
LGALS3	4795.698162	2522.13928	0.52591702	3.71E-28
TUB	1152.46614	605.8305322	0.52568185	2.26E-17
PDCD4	1804.968508	948.6993142	0.52560436	1.55E-16
LOC400027	301.3474571	158.2825427	0.52524931	8.04E-08
NBR2	165.4127294	86.84760219	0.52503578	3.84E-05
RPRM	338.8137655	177.8793572	0.52500629	2.33E-05
TRPC1	1152.955138	605.2476226	0.52495332	2.13E-17
PIR	748.1984165	392.6301712	0.52476744	8.08E-14
ZNF425	192.1809677	100.8277957	0.52465027	1.10E-05
ADRBK2	1505.211835	789.4515948	0.52447873	8.13E-20
BTBD7	2443.384208	1281.422834	0.5244459	2.20E-18
AGL	2060.230657	1080.033892	0.5242296	1.84E-22
SPHAR	270.3577598	141.6427028	0.52390841	3.04E-07
NEK8	205.3584129	107.57719	0.5238509	4.84E-06
DLX6	241.0448124	126.2559875	0.52378637	1.17E-06
EGR1	1683.026894	881.545298	0.52378563	9.28E-21
SMPDL3A	1648.232524	863.1119361	0.52365909	1.17E-20
PIPOX	7673.73803	4014.867449	0.52319579	5.33E-31

DIP2C	1256.474439	657.083382	0.52295802	2.47E-18
SALL4	393.810358	205.9294161	0.52291518	2.53E-09
KDM5B	2843.23032	1486.729195	0.52290143	4.76E-25
CERCAM	1768.643826	924.7130568	0.52283735	1.03E-05
SPARC	134591.6732	70355.96818	0.52273641	8.72E-36
FAM55C	6116.013128	3194.057147	0.52224498	4.77E-30
ATP7A	770.1771094	402.1312501	0.52212828	7.40E-14
MBD5	681.9356676	355.9734706	0.52200448	4.29E-10
KIAA0232	3281.963017	1713.088504	0.52197069	3.42E-08
EFHC1	1539.417487	803.306704	0.52182511	3.21E-20
GPLD1	119.7545232	62.45777882	0.52154839	0.0003292
FBXL17	1524.69396	794.3980009	0.52102128	2.75E-20
ST5	2680.730234	1396.185945	0.52082299	7.58E-25
SORL1	734.1530121	382.2842668	0.5207147	5.64E-14
AHCYL2	1442.264761	750.6803784	0.52048722	8.29E-20
APLP1	1228.538368	639.1007458	0.52021228	1.39E-08
BBS2	3806.373118	1979.200772	0.51997025	1.16E-27
ANK2	1041.464724	541.3904216	0.51983558	3.86E-07
RBL2	5124.930822	2662.266942	0.51947373	1.31E-29
AASS	2134.258201	1108.362432	0.51931975	2.51E-23
LONRF2	2240.701172	1163.608258	0.51930542	4.89E-17
ENOX1	408.49606	212.1289467	0.51929252	9.07E-10
KSR2	294.4953231	152.9089908	0.51922383	7.81E-08
MUC1	108.1953711	56.10721749	0.51857318	0.0005972
CTSO	849.3662834	440.4399081	0.5185512	2.31E-08
JHDM1D	316.0125273	163.8661176	0.51854311	2.13E-07
ARHGEF37	261.0365666	135.294508	0.51829715	2.90E-07
ATP1B1	8071.987193	4180.692318	0.51792603	3.94E-32
LOC440104	368.6157106	190.8849079	0.51784257	3.09E-09
C6orf57	221.0599354	114.4138895	0.51756954	1.72E-06
UNC5C	1341.961415	694.3607713	0.51742231	1.69E-19
TMEM168	1641.115615	849.0326046	0.51735088	3.90E-14
KIAA1244	2393.422016	1237.731158	0.5171387	8.76E-15
NPY1R	686.6942652	354.3545591	0.51602959	8.60E-14
GALNT1	3721.137874	1918.781951	0.51564387	4.29E-28
KLHL9	2836.874061	1462.40783	0.51549974	4.31E-26
TRIM45	680.0243788	350.4134754	0.51529546	1.09E-13
TMEM167A	11314.118	5829.036198	0.51520023	8.87E-25
ZKSCAN1	2343.996962	1207.38724	0.51509761	1.61E-11
YPEL2	383.3082899	197.292095	0.51470866	1.56E-09
PCDHB2	148.0447729	76.19490329	0.51467473	5.42E-05
EML1	3703.201831	1902.670761	0.51379073	2.18E-28
CCPG1	1324.490299	680.4395701	0.51373692	8.55E-11
ARHGEF10L	850.8703898	436.3430605	0.51281966	1.77E-09
DHRS1	313.5090818	160.7557457	0.51276264	1.75E-08
KIRREL	6105.908518	3128.583401	0.51238622	1.15E-31
THAP2	442.6260618	226.6896834	0.51214717	1.27E-10

ERMP1	1240.6113	635.320159	0.51210251	2.89E-19
GALNT11	1822.522151	932.6777965	0.51175114	8.66E-23
TMTC1	4511.096981	2307.416779	0.51149793	4.96E-30
ORAI3	753.6867162	384.5710266	0.51025316	2.09E-11
RAB11FIP4	144.4464183	73.66034134	0.50994924	5.74E-05
PLXNC1	383.8076036	195.6141912	0.50966732	8.77E-10
RCN2	12167.51247	6200.287866	0.50957728	9.83E-35
WDR61	7505.387164	3822.13476	0.50925218	2.42E-33
TTC28	6203.231161	3156.05765	0.50877641	2.23E-32
EPM2AIP1	1759.857757	894.6711999	0.508377	7.52E-23
C12orf76	328.7387998	167.101574	0.50831108	7.73E-09
PLCD3	6812.696273	3462.257899	0.5082067	1.48E-11
GABRQ	117.079146	59.49607106	0.50816967	0.000244
ATRX	4776.259436	2425.863791	0.50790034	3.47E-13
GPR155	390.1260376	198.0873943	0.5077523	5.50E-10
LAMB2	6486.732571	3293.33449	0.50770314	1.57E-09
HIP1R	659.7396289	334.8804624	0.50759489	1.80E-06
SH3BGRL	3905.881428	1981.386027	0.50728269	1.19E-29
PLEKHG1	12448.17902	6305.986595	0.50657904	7.78E-36
LOC100129	2037.239582	1030.973397	0.50606389	1.78E-24
HSD17B14	192.7559313	97.53334707	0.50599401	3.37E-06
TMEM183A	184.8054496	93.44359915	0.50563227	4.68E-06
ZNF33A	1054.204751	532.3212216	0.50495051	2.31E-18
SIAE	1694.386605	855.2038223	0.50472768	5.93E-23
KIAA0182	2076.940411	1048.236292	0.50470215	5.39E-25
LOC729970	685.1172357	344.9549848	0.50349775	1.12E-12
ZFP112	707.2162808	356.0300964	0.50342463	8.20E-15
PLSCR4	1120.525957	563.7035083	0.50307046	4.01E-19
GPR64	91.34048279	45.94302332	0.50298643	0.0009105
PIK3C2B	213.7016106	107.3010749	0.502107	9.48E-07
MSI1	761.5065298	382.0105183	0.50165101	1.24E-15
SORT1	9663.873757	4846.509793	0.50150798	7.19E-36
ZNF528	206.8769857	103.6077933	0.50081836	1.21E-06
IFT140	928.0966955	464.7943189	0.50080376	3.05E-09
SSPN	1085.721271	543.4010663	0.50049776	5.05E-19
SOX21	6272.723618	3137.725707	0.50021743	7.64E-34
FKBP9L	112.8920734	56.44469148	0.49998808	0.0002139
HHAT	387.5091171	193.6294929	0.49967726	2.83E-10
ZNF345	173.7696816	86.81692273	0.49960915	7.09E-06
WSCD1	2334.733514	1166.088561	0.49945253	1.30E-26
CREBL2	3397.250515	1696.604428	0.49940516	8.40E-30
ING4	1420.547404	709.1952565	0.49924082	7.60E-22
LMLN	3930.783791	1961.789213	0.49908347	5.87E-26
ABCA2	1516.012353	756.3512587	0.49890837	1.97E-05
COL4A3	609.5367322	304.0739857	0.49886081	7.92E-14
NT5DC3	3226.79722	1608.270098	0.49841065	1.24E-29
FAM69A	1521.061219	758.1070872	0.49840669	9.53E-23

PPAP2B	3692.069781	1839.842462	0.49832278	1.07E-30
LOC729678	610.0704322	303.8026037	0.49797956	5.80E-14
ODZ2	11377.08843	5663.680987	0.49781462	3.86E-37
CCDC103	866.902733	431.4603799	0.49770333	4.36E-17
C1orf63	1834.339201	912.8284467	0.4976334	2.06E-24
KIAA1671	3776.041623	1877.693295	0.49726499	5.06E-31
GPR173	266.2153893	132.3634797	0.49720446	4.92E-08
SPAG17	271.4354757	134.9287211	0.49709317	3.54E-08
TRPS1	15579.65714	7744.207525	0.49707176	5.00E-17
MFAP3L	2721.878136	1352.428262	0.49687319	2.02E-28
SLC25A1	3079.794353	1527.216094	0.49588249	4.46E-17
KIF27	142.2909866	70.54760285	0.49579811	3.22E-05
NACC2	4595.187039	2278.085283	0.49575464	2.57E-12
MPDZ	5742.036347	2845.508019	0.4955573	2.68E-24
MXRA7	6118.784075	3031.783995	0.49548799	1.22E-34
APPL2	2809.613387	1390.356934	0.49485703	7.92E-29
AGBL2	156.43955	77.38430236	0.49465945	1.45E-05
CYP2U1	1165.051429	576.2842797	0.49464278	2.23E-20
PLXNB1	1119.864315	553.7540703	0.49448318	9.98E-08
MSI2	5604.242622	2768.28658	0.49396266	1.48E-15
HEXIM1	9576.576515	4726.262074	0.49352314	3.21E-37
SFRP4	105.5337485	52.04814902	0.49318962	0.0002776
NFIB	4279.769516	2109.114629	0.49281033	1.08E-18
SOX4	3401.189294	1674.444739	0.49231154	6.24E-31
TTC30B	310.2862501	152.724914	0.49220652	3.88E-09
PPAPDC2	471.997466	232.2756249	0.49211202	4.17E-12
FZD7	2754.716515	1355.503222	0.49206632	3.92E-29
SPIRE2	889.142762	436.9283367	0.49140403	6.52E-18
ETV6	3507.329665	1723.476921	0.49139291	2.10E-31
C17orf108	95.56881907	46.9507122	0.49127647	0.0004772
PRKCA	5092.254059	2500.196713	0.49098036	2.66E-34
ZNF862	606.5099026	297.7517373	0.49092642	3.26E-14
PRRX1	6494.855104	3187.357365	0.49075111	6.32E-36
PNMA2	1665.459497	817.2892641	0.49072899	2.04E-24
KBTBD7	295.6658821	145.0315564	0.49052517	7.10E-09
ZMAT3	2772.24336	1359.533977	0.49040932	1.85E-29
BBS1	749.3930459	366.8645055	0.48954885	2.76E-16
PIK3R2	5584.743896	2733.772622	0.48950725	2.52E-28
CA8	1313.729622	642.4046606	0.48899305	3.49E-22
VASH1	109.7586461	53.63638099	0.48867568	0.0001709
ZNF671	222.1342127	108.5187869	0.48852802	3.13E-07
INSIG1	14428.09394	7043.800621	0.48820036	5.57E-40
LOXL4	893.1854121	435.8876018	0.48801469	3.37E-18
KIAA1377	321.3529658	156.814662	0.48798262	1.66E-09
TIMP2	28438.9585	13871.17489	0.48775256	8.87E-42
ZFAND5	16116.29693	7860.514075	0.48773699	1.92E-40
ZNF596	155.3583955	75.76539094	0.48768134	0.0007472

ICA1L	439.9506846	214.2670423	0.48702514	7.19E-12
MOSC1	502.4259542	244.6416401	0.48692079	5.66E-13
C3orf47	116.621096	56.77979893	0.48687417	0.0001099
GSTA4	1792.352272	872.2353954	0.48664284	1.62E-20
MIR3064	161.2153408	78.45335016	0.486637	7.09E-06
KATNAL2	111.3563075	54.18624461	0.48660238	0.0001396
PBLD	321.3117022	156.1444471	0.48595942	1.26E-09
SLC35D2	1536.621758	746.7038822	0.48593864	1.19E-12
GAD1	309.304816	150.1903521	0.48557392	2.39E-09
SNTB1	943.5437591	457.9906654	0.4853942	5.00E-19
EIF4E3	774.8738117	376.0847362	0.48534965	2.21E-13
GSN	6162.539173	2990.435704	0.48526032	1.76E-11
PPARA	1732.979482	840.529834	0.48502007	8.16E-13
THRA	2300.145462	1114.009733	0.48432143	1.49E-28
PDE1C	13138.43524	6359.214677	0.48401614	8.66E-18
TMTC4	886.2816986	428.8951385	0.48392643	1.94E-18
PDGFRA	8166.613115	3949.540001	0.48362031	2.83E-28
PRLR	4726.055828	2285.117891	0.48351479	2.69E-18
ZBTB46	151.2538501	73.05148535	0.48297273	9.83E-06
RNF170	989.5843654	477.8659615	0.48289563	6.99E-14
BCHE	5394.521055	2602.825131	0.4824942	9.59E-28
FGFR1	5247.060849	2530.809647	0.48232901	4.91E-36
DSCR8	68.74900131	33.15222883	0.48222124	0.002797
ATP9A	3951.488055	1901.004605	0.48108575	5.55E-34
GPR137B	1435.563927	690.2993363	0.48085587	5.18E-24
PLCB4	98.18917813	47.10174294	0.47970401	0.0003067
SDC2	5049.787737	2421.759843	0.47957656	3.26E-36
TSPYL4	1510.483501	724.1831389	0.47943797	9.84E-25
FUCA1	1106.983304	530.6739973	0.47938753	2.77E-21
TRPM3	1030.748749	493.8615329	0.47912892	1.38E-20
HS6ST2	4127.23092	1976.618965	0.47892134	7.42E-34
MAGI2	397.4740465	190.2736853	0.47870719	1.66E-11
LSS	4896.513865	2343.759585	0.47865883	4.93E-16
ULK2	2083.311136	996.874838	0.47850502	2.60E-28
RAB31	20199.65441	9661.207787	0.4782858	1.28E-32
JAKMIP2	2191.647491	1047.922398	0.47814368	5.51E-29
L3MBTL1	99.29096444	47.46752984	0.47806495	0.0018745
LOC10049	4034.351233	1927.851151	0.47785903	7.67E-35
TMCC1	4300.518481	2054.774987	0.47779704	2.47E-35
FAM172A	1772.611538	846.8638295	0.47774925	1.30E-26
VPS13C	2507.794837	1197.739863	0.4776068	1.71E-13
MEGF9	2478.516276	1183.639318	0.47755963	1.73E-20
ZNF608	1026.506658	489.5546622	0.47691328	1.04E-20
PTPRG	3631.364816	1731.708309	0.47687533	8.06E-18
KLHDC7A	1062.802407	506.593335	0.47665806	3.32E-21
TNFRSF19	1637.076404	779.6460879	0.47624295	5.91E-26
WDR60	684.6866947	326.0236521	0.47616473	1.72E-16

C5orf42	1603.872818	763.3248753	0.47592606	1.21E-17
SAMD11	133.3934571	63.40174227	0.47529874	2.68E-05
SEMA3B	2064.474763	981.1152361	0.47523721	2.59E-05
PTK7	9708.006392	4612.131399	0.47508533	4.27E-27
GPRC5B	2580.261833	1225.074914	0.47478705	2.96E-31
POLI	482.499534	228.8301455	0.47425983	2.15E-13
LOC644172	111.8934461	53.05583791	0.47416395	8.96E-05
SEPP1	101.3501144	47.95603456	0.47317198	0.0002036
SLC22A15	198.4271904	93.74566061	0.47244362	3.66E-07
LSAMP	2674.047305	1262.302692	0.47205698	7.02E-32
OGFRL1	2495.151092	1177.411475	0.47187983	3.27E-12
TMEM106B	5438.499072	2564.964832	0.47163101	1.24E-22
C4orf3	3149.842566	1484.237145	0.47120995	9.95E-34
MAGED2	5402.208776	2544.8512	0.47107606	2.20E-38
LOC100499	90.34185549	42.55416974	0.47103493	0.0003759
PSD3	8767.700763	4129.085429	0.47094279	1.28E-15
PCDHB9	202.6383335	95.33389257	0.47046327	2.36E-07
LOC100506	238.8996966	112.3371528	0.47022727	2.76E-08
CALCOCO1	1791.573717	842.2336841	0.47010831	6.83E-28
PPAPDC1B	1246.037705	584.9239673	0.46942718	9.22E-24
ZNF248	655.8008496	307.7389544	0.46925672	1.37E-16
KIAA0825	89.25726235	41.88158831	0.46922331	0.0004107
RFX3	756.5141051	354.9657817	0.46921238	1.80E-17
SMPD1	1283.001261	601.8611356	0.46910409	4.90E-24
FANK1	93.4615282	43.83560718	0.46902301	0.0002936
TACR1	2320.127612	1087.875914	0.46888624	6.85E-31
C10orf140	234.6954307	110.017347	0.46876646	3.10E-08
HECTD2	1267.382977	594.106419	0.46876629	6.21E-24
LOC338799	197.962263	92.67661281	0.46815293	3.01E-07
TRIM13	607.4260027	283.4081234	0.46657226	5.55E-16
ZNF860	123.8590687	57.78748782	0.46655839	0.0007015
PELI2	1351.878203	630.6805474	0.46652172	1.66E-22
NES	20862.00811	9731.854357	0.46648694	6.15E-46
SEL1L3	9150.175481	4259.780471	0.46554085	4.46E-21
ODZ3	3979.127691	1852.201378	0.46547925	2.99E-34
BHLHE40	4599.830147	2137.129275	0.46461048	2.72E-38
THSD7A	157.4588091	73.11047772	0.46431494	9.79E-05
ANKRD1	2657.130521	1232.124003	0.46370473	2.32E-29
RAPGEF1	7981.746361	3692.983071	0.46267858	9.56E-43
CRIPAK	224.7752035	103.7895035	0.46174801	3.42E-08
CD24	5335.606315	2461.99894	0.46142815	6.67E-24
YPEL5	2493.790696	1148.20033	0.4604237	1.95E-26
PARD3B	582.3551461	268.1134464	0.46039508	5.82E-16
RFTN2	182.6981588	84.1006506	0.46032566	4.11E-07
TMEM80	345.0840586	158.8300397	0.46026478	4.58E-11
HGSNAT	2845.916014	1307.452782	0.45941369	1.24E-34
COL1A2	29311.74284	13459.64161	0.4591894	1.44E-48

RRAGB	630.1894158	289.3055925	0.4590772	4.79E-17
RAB11FIP1	63.5392309	29.15215273	0.45880556	0.0021825
C17orf100	185.4079223	85.01630113	0.45853651	3.16E-07
ZBED6	1237.326573	567.1230413	0.45834548	5.87E-12
TOM1L2	8380.235516	3838.215184	0.45800803	4.46E-32
CCDC30	148.5922274	68.0130409	0.45771601	3.54E-06
ZIC4	89.27101689	40.81490705	0.45720222	0.0002414
MALAT1	10496.5489	4799.018683	0.45719967	6.51E-12
TMEM117	548.6900716	250.7160864	0.45693571	1.14E-15
KIAA1161	1068.573387	488.2094994	0.45687971	3.48E-23
ZNF624	250.45541	114.4138895	0.45682339	4.44E-09
CTSK	218.976715	99.97587066	0.45655937	2.82E-08
NFIX	4472.014394	2041.004816	0.45639496	1.10E-39
IFT80	3913.463976	1785.651485	0.45628412	2.09E-31
MID1	8605.80315	3921.553667	0.45568712	1.17E-22
SAMD4A	1528.381719	696.2817442	0.45556796	7.09E-28
LRP1B	782.789907	356.5233342	0.45545213	1.01E-19
ALPK2	3583.767812	1631.921248	0.45536467	3.30E-33
ABCA5	1060.375324	482.805268	0.45531545	3.72E-09
RCCD1	59.34528095	27.01642367	0.45524131	0.0025778
GFRA1	6262.198192	2848.986544	0.45494992	7.92E-43
PCDHB16	288.2009595	131.1127217	0.45493506	3.75E-10
ALDH5A1	1849.830967	840.8861547	0.45457459	2.17E-30
ACCS	151.2057092	68.68562234	0.45425284	2.26E-06
CYYR1	83.51379196	37.91455819	0.45399158	0.0003505
COL24A1	104.4491553	47.37785802	0.45359733	6.70E-05
SOX5	924.6331594	418.7923032	0.45292806	7.95E-22
RALGPS1	443.2250958	200.2844822	0.45187983	7.58E-14
BOC	1355.906387	612.3344908	0.45160529	7.16E-27
PMP22	16158.41796	7294.063615	0.45140952	6.50E-49
FAM13C	605.8799209	273.4563189	0.45133748	4.55E-17
APOLD1	1735.713316	781.8785884	0.45046528	3.36E-30
ZNF181	636.3634272	286.4618695	0.45015451	9.89E-18
NUAK2	1722.008336	775.1646067	0.45015148	3.73E-30
NAP1L5	880.5863691	396.3258193	0.45007035	1.57E-21
PALM	153.4058432	69.02072979	0.44992243	0.0002575
C1orf226	1626.725636	731.6334275	0.44975834	1.74E-29
FKBP9	4726.420323	2124.326733	0.44945785	9.38E-42
RHOJ	2952.300528	1326.52568	0.44931932	9.56E-37
SATB1	180.1190633	80.74484302	0.44828594	1.93E-07
ARMCX3	2870.026068	1284.490694	0.44755367	9.79E-37
DMGDH	63.52547636	28.42057892	0.44738868	0.0014995
DNAH7	91.89137595	41.08865559	0.44714376	0.00015
SULF2	3728.127553	1666.659343	0.44704998	8.58E-40
NRXN3	424.2491621	189.2943094	0.44618664	9.33E-14
C20orf108	3357.494668	1497.365414	0.445977	1.17E-34
HEATR5A	4546.828082	2027.362097	0.44588492	4.93E-42

TRIM16	3912.777552	1741.421778	0.44506026	1.03E-40
NOS1AP	399.0785851	177.3247605	0.44433544	3.41E-13
CYP4V2	2035.61785	902.3976036	0.44330403	2.44E-33
GLI3	3775.669539	1673.477195	0.44322661	8.31E-41
RAB40B	293.5207662	130.0436739	0.44304761	8.95E-11
CCDC144C	75.04680349	33.24426719	0.44298046	0.0019366
WWC3	2645.085098	1170.572409	0.44254622	9.67E-37
SALL2	334.0139042	147.7808745	0.44243929	6.93E-12
PCMTD2	2727.07759	1206.322925	0.44235006	3.25E-37
BDNF-AS1	152.8583887	67.61657455	0.44234782	1.10E-06
C17orf103	388.1769238	171.6515137	0.44219917	1.87E-08
POPDC3	3031.503457	1338.709985	0.44159936	1.56E-38
DDR1	9708.700996	4284.908968	0.4413473	5.79E-16
C17orf69	178.9966452	78.88049597	0.44068142	1.48E-07
NTRK3	1201.247458	528.965414	0.44034675	1.12E-26
LGR4	20044.31736	8822.660199	0.44015768	5.30E-29
PLEKHH1	278.3598209	122.501347	0.44008272	2.04E-10
ARHGEF4	4165.742573	1831.868257	0.43974591	3.43E-32
SRGAP3	2051.016061	900.53089	0.43906574	4.83E-34
PLB1	85.55574879	37.54877129	0.43888075	0.000169
ACO1	1902.38257	834.8754339	0.4388578	3.43E-33
PAR5	235.1775512	102.9965708	0.43795239	2.41E-09
KIFAP3	3740.854537	1635.862332	0.43729643	7.41E-42
NDST3	80.8418534	35.34931679	0.43726505	0.0002732
TXNRD3	382.7780286	167.3163301	0.43711059	2.44E-13
C11orf75	260.4272167	113.7436746	0.43675802	3.57E-10
KLHDC1	69.28957856	30.25187998	0.43660072	0.0006967
TPPP3	79.28201704	34.58706353	0.43625358	0.0002802
TMEM170I	1537.152019	670.4547196	0.43616683	1.85E-18
ST6GAL1	65.09562862	28.38989947	0.43612605	0.000985
COL1A1	227.4127558	99.17820487	0.4361154	4.35E-09
ASAH1	9193.832282	4009.003026	0.43605353	5.36E-50
TYRP1	90.31434642	39.38007235	0.43603341	0.0001058
ASB1	9127.71182	3977.696212	0.43578241	6.64E-50
ZNF792	66.13551952	28.81704528	0.43572721	0.0009042
ERMAP	892.128328	387.9056209	0.43480922	2.26E-23
CHN1	23532.57682	10225.11659	0.43450901	9.92E-55
FZD5	1189.733008	516.6300783	0.43424035	3.00E-27
CAT	3916.69985	1698.287065	0.43360153	4.22E-43
AFF2	1078.796926	467.7324468	0.43356858	6.29E-13
COL4A6	2259.507901	979.1635838	0.43335258	3.06E-36
SERINC5	33852.69845	14618.29172	0.43182058	2.22E-56
SLC16A4	2543.428945	1097.308534	0.43142881	2.82E-38
CHURC1	3262.218133	1405.821574	0.4309404	1.15E-41
PCMTD1	1696.938192	731.0434183	0.43080144	1.12E-32
CLIP4	2579.704063	1111.130597	0.43072018	1.69E-38
SERTAD4	96.09564179	41.33409122	0.43013492	5.03E-05

SIDT2	651.716936	280.2953849	0.43008762	1.18E-19
GATS	227.2958422	97.71505725	0.42990253	2.15E-09
PROS1	1024.847101	439.6469754	0.42898787	6.66E-26
C14orf37	279.91278	119.9408386	0.42849361	4.33E-11
ARL4C	12511.83718	5358.453014	0.42827068	8.26E-54
PODXL	21514.7334	9207.531006	0.42796398	1.38E-53
SLC15A2	60.91199457	26.03941424	0.42749239	0.0011433
ACTG2	1620.572256	692.7678063	0.42748344	1.35E-32
MIR181A2I	53.0096538	22.65056067	0.42729124	0.0024665
KCND3	89.80471687	38.33933747	0.42691897	0.0001273
C1orf101	49.90373563	21.27708487	0.42636257	0.0032218
SYNC	1263.681464	538.034614	0.42576759	2.54E-25
CCDC146	501.9232019	213.4410636	0.42524646	6.83E-17
HMCN1	3606.022308	1533.200869	0.42517787	6.61E-16
C1orf183	633.2265613	268.3305691	0.42375129	6.76E-20
ENC1	16656.62933	7049.1058	0.42320122	9.94E-57
KIF26B	1484.720056	627.9076494	0.42291316	2.17E-23
RHOB	9429.112872	3984.915264	0.42261826	7.23E-35
NR2E1	638.9562772	269.7960833	0.42224498	3.74E-20
CYBRD1	11587.82167	4890.350048	0.42202497	4.47E-55
MT1F	286.1761959	120.7314048	0.42187787	1.80E-11
PGCP	1702.8364	717.3747523	0.42128225	2.33E-34
HOGA1	122.3233028	51.52896485	0.42125224	3.90E-06
ALDH3B1	1637.113517	688.3170045	0.4204455	8.40E-17
TSHZ2	594.8200824	250.0458715	0.42037228	2.02E-19
VCAN	54037.70179	22714.99909	0.42035465	6.06E-20
FAIM2	655.9590268	274.954879	0.41916472	9.47E-21
PCDHB14	543.2911764	227.6666928	0.41905097	7.94E-09
STON2	429.3592121	179.8333761	0.41884131	8.35E-07
NCRNA002	181.7236019	76.07218548	0.41861478	2.61E-08
LIMCH1	11413.84982	4775.973937	0.41843672	3.83E-37
H6PD	3066.118306	1281.750842	0.41803698	2.12E-43
KLHL3	73.54198528	30.74038469	0.41799775	0.0002708
HS3ST1	275.6810051	115.1147838	0.41756516	2.31E-11
NPY2R	211.5874424	88.31311634	0.41738354	2.34E-09
FAM120C	1260.114057	525.4845221	0.41701346	2.05E-30
ABCA13	5670.219965	2363.658547	0.41685482	3.60E-16
ZNF25	964.4963157	401.6970046	0.41648371	2.29E-26
CRELD1	711.1309896	296.0431541	0.41629905	8.67E-17
ZNF385B	64.55505137	26.86302641	0.41612586	0.0006222
PREP	10903.03526	4532.295193	0.41569114	1.34E-56
SOX2OT	22298.00543	9262.715474	0.41540556	2.36E-53
PAQR6	158.6052977	65.87494531	0.41533887	1.52E-07
PLXNB3	383.4355194	159.254819	0.41533664	1.43E-09
SH3YL1	400.5902806	166.3086412	0.41515895	4.30E-15
EFNB3	138.5929117	57.51137274	0.41496619	7.42E-07
FGF1	321.3495272	133.2814968	0.41475554	5.49E-13

RNF175	81.35148295	33.70209245	0.41427754	0.0001067
CUBN	311.8426478	129.1893823	0.41427747	1.11E-12
CHST9	61.93125367	25.64294788	0.41405504	0.0006903
LOC100507	131.2655345	54.30659589	0.41371557	1.25E-06
ZFP3	194.2401177	80.31533067	0.41348477	6.46E-09
APCDD1	794.5175521	328.3741373	0.41330004	6.47E-24
SPARCL1	842.2245924	347.7255162	0.41286555	9.44E-25
GPM6B	8730.074854	3604.316	0.41286198	6.72E-44
FAM92B	58.82189687	24.23879263	0.41207091	0.0008929
COL9A3	115.526187	47.59024765	0.41194338	4.79E-06
ITGA11	69.84391035	28.75568637	0.41171358	0.000329
RAB26	252.7243167	103.8815419	0.41104688	0.0009668
TCP11L2	468.7815115	192.4400938	0.41051127	7.81E-13
TBCK	789.6626727	323.9162359	0.4101957	3.88E-24
LOC646903	149.6940137	61.36041811	0.40990562	1.68E-07
WNT5B	464.3358295	190.3043648	0.40984209	9.19E-10
GPR1	475.0689978	194.6678612	0.40976755	4.57E-11
BMPR1B	1224.734408	501.0899657	0.40914174	9.34E-14
DPYSL2	4815.494626	1969.152196	0.40892003	3.56E-51
PBX1	6344.441704	2586.746987	0.40771862	3.37E-48
CELSR2	5495.775479	2239.155936	0.40743221	4.22E-22
TP53INP2	1355.094158	551.9487156	0.40731392	6.36E-33
PDE9A	368.5606924	150.1006803	0.40726177	9.43E-15
NEFL	49345.3709	20083.91844	0.40700714	7.26E-65
PIGZ	395.4871078	160.6920202	0.40631418	1.63E-15
SALL1	711.9576852	288.7864084	0.40562299	2.76E-23
LOC100272	46.72216752	18.92659964	0.40508822	0.0027496
CDRT1	78.24556477	31.68671468	0.40496499	0.00011
TARSL2	193.7304881	78.45335016	0.4049613	3.37E-09
DCAKD	3763.967979	1524.195394	0.40494377	3.78E-38
CAPN3	108.7015621	43.98900444	0.40467684	5.79E-06
PCDHGA9	537.0759014	216.7685583	0.4036088	2.10E-19
ADAM12	10891.5112	4395.688568	0.40358849	1.84E-41
IPW	740.2754439	297.9098677	0.40243111	4.50E-24
GPC4	12110.93268	4867.248761	0.40188885	1.49E-34
TMEM150C	232.0578785	93.0754457	0.4010872	1.07E-10
SEMA5A	249.3880101	99.8838323	0.40051578	2.35E-11
ERV3-1	274.5792188	109.9583547	0.40046131	3.11E-12
MID2	489.8647361	196.1640549	0.40044535	1.70E-18
GSC	131.7854799	52.71836392	0.40003166	5.92E-07
ZNF606	1112.875348	444.1308231	0.39908407	5.95E-12
PDE8B	506.1033973	201.9317066	0.39899299	5.67E-19
FAM8A1	2342.181955	934.0229593	0.39878326	1.82E-43
NUPR1	2961.234459	1178.973761	0.3981359	1.61E-16
HRCT1	358.3405924	142.6197122	0.39800044	0.0010689
COL4A4	865.2810012	344.3437622	0.397956	4.55E-27
NDP	268.3983302	106.7205318	0.39761995	4.45E-12

ARID5B	6761.625537	2684.575296	0.39703105	2.27E-36
CUL7	1273.518452	505.2458056	0.39673222	1.53E-33
PPIEL	104.4491553	41.30341177	0.39544036	1.26E-05
HIST2H2BE	525.6474174	207.8551221	0.39542689	8.42E-20
LIX1	79.27170114	31.25956887	0.39433453	6.54E-05
ADAM23	1302.187663	512.9344302	0.39390208	2.83E-34
C6orf112	180.5839906	71.12577939	0.39386537	4.74E-09
SLC35E2	306.6053683	120.7337714	0.39377579	1.28E-13
MYLK	8489.439239	3341.498181	0.39360647	3.90E-61
WBP1	49.38722881	19.41510436	0.39311994	0.0015665
GRIA2	56.16027421	22.07001759	0.39298272	0.0008034
LOC729013	286.6651936	112.3678323	0.39198282	5.52E-13
ADAMTS16	326.555859	127.8442194	0.39149265	2.58E-14
TCF7L1	1032.962637	404.3259715	0.39142362	9.39E-31
C14orf45	270.3302507	105.7458889	0.39117298	2.79E-08
LPGAT1	6836.521041	2660.376649	0.38914188	2.42E-27
SCD	202988.4985	78967.03053	0.38902219	2.24E-44
TTC18	85.04955787	33.06019047	0.38871678	3.02E-05
SDK1	3752.491354	1458.155219	0.38858323	1.02E-29
PRSS12	40673.53315	15783.35839	0.38804985	4.86E-49
LOC100329	128.6348595	49.91241996	0.38801628	3.08E-07
SLC46A3	321.3632817	124.4553658	0.38727314	2.28E-14
FOXO1	708.2424171	273.39496	0.3860189	4.38E-25
AMT	85.57294196	33.02951102	0.38598078	2.60E-05
C11orf87	202.156213	78.02620435	0.38596986	3.16E-10
GSTM2	246.3130396	94.96810567	0.38555858	6.89E-12
CFI	4960.317279	1910.298027	0.3851161	1.98E-38
SPATA6	1068.94891	411.5591374	0.38501292	3.17E-32
FAM134B	1431.872729	551.1840958	0.38493931	1.97E-37
ZDHHC15	722.9521896	278.1903353	0.38479769	1.23E-25
FRY	603.1667187	232.0018764	0.38463972	1.07E-13
CBLB	1689.993214	649.1186423	0.38409541	2.79E-33
HIST1H3E	51.9800788	19.96496799	0.38408884	0.000887
SLC2A10	2658.521153	1020.502408	0.38386093	9.24E-49
CDH11	24187.54066	9276.601348	0.38352809	4.25E-71
SEMA6A	720.53471	275.8705295	0.38286917	8.69E-26
ST7-AS1	48.82601976	18.68116402	0.38260674	0.0014717
ZFP14	380.5847719	145.5531071	0.38244596	1.88E-11
DCDC2	59.33152642	22.68124012	0.38227973	0.0003905
ALDH3A1	179.1101201	68.44018671	0.38211234	2.00E-09
TTC39A	133.9168412	51.10181904	0.38159367	1.39E-07
NEAT1	15192.11008	5794.161273	0.38139279	1.90E-69
ATRNL1	1766.014575	672.6258612	0.3808722	1.92E-19
TOX	4035.108444	1535.94782	0.38064598	2.93E-56
BEND6	414.6769487	157.5792818	0.38000492	5.87E-09
C21orf34	461.4438184	175.1300391	0.37952624	2.99E-16
LRP1	986.8945219	374.4681913	0.37944095	1.41E-18

LOC283624	430.998137	163.4389718	0.37921039	2.54E-17
GTF2IRD2	40.94774942	15.50706662	0.37870376	0.0031754
MAF	1378.897462	521.180018	0.37796865	5.16E-38
PLCD4	287.1851391	108.3701227	0.37735282	7.74E-14
PLEKHB1	1791.54277	675.7079202	0.37716539	3.52E-43
SCN3A	211.0709356	79.52239796	0.37675674	5.71E-11
HEY1	2309.308478	870.0335744	0.3767507	7.60E-48
ITIH5	2820.7867	1062.332104	0.37660845	1.22E-51
KCTD12	3068.42231	1154.513112	0.37625626	5.18E-53
GPR98	2692.211009	1011.13588	0.37557824	8.61E-32
DHCR24	98996.35282	37064.20036	0.37439966	9.31E-78
MXD4	921.0547249	344.0062882	0.37349169	0.0003885
JAKMIP3	351.8914903	131.4171497	0.3734593	2.55E-16
LOC100192	267.7752257	99.94519121	0.37324286	2.34E-13
PABPN1	96.12315086	35.86850096	0.37315153	4.77E-06
SLC26A9	348.2106085	129.7368794	0.37258164	3.37E-16
NCRNA000	333.4802042	123.9975406	0.37182879	1.01E-15
PCDHB10	213.6809788	79.4303596	0.37172405	2.84E-11
CALD1	27251.55381	10089.29097	0.3702281	4.23E-45
KLRAP1	70.33978537	26.00873478	0.36975852	7.21E-05
RBMS3	610.0738708	225.5309638	0.36967812	1.79E-24
C20orf112	1275.240616	471.2982775	0.36957596	2.92E-34
3-Mar	402.2601532	148.359051	0.36881369	2.47E-18
FOS	1422.695959	523.8939236	0.36824026	2.92E-40
MIR5047	1296.175979	477.1296547	0.36810561	4.06E-22
FABP7	73848.52273	27131.81143	0.36739816	2.88E-80
ZNF117	536.0360105	196.8625827	0.36725626	1.65E-22
C1RL	2299.674369	844.0861985	0.36704597	5.28E-50
LOC90246	193.1899109	70.79067194	0.36643048	1.30E-10
GNRH1	57.24142871	20.84993906	0.36424561	0.0002814
SLC27A6	146.5640251	53.32958645	0.36386546	3.73E-07
ESRRG	380.0545105	138.2231698	0.36369301	4.09E-12
MEGF10	228.3941899	83.00092335	0.36341084	3.31E-12
ALDH1L2	3166.274502	1150.151982	0.36325087	3.33E-32
C10orf114	227.9017535	82.63513645	0.36259105	3.03E-12
DGCR5	78.25244204	28.29786111	0.36162272	2.19E-05
PCDHGB2	2311.440551	833.9928294	0.36081085	6.66E-19
GALNT5	1728.754584	621.6798059	0.35961137	4.26E-21
DACH1	2799.400876	1002.250756	0.35802331	2.72E-49
LOC646851	319.2181659	114.1708204	0.35765765	3.07E-16
COL2A1	38.32395173	13.70644501	0.35764696	0.002688
NRBP2	2543.228793	909.5387311	0.3576315	2.55E-54
MAGED1	11918.80045	4218.899472	0.35397014	8.89E-78
PRUNE2	2149.990671	760.9672904	0.35393981	7.05E-52
COPZ2	309.8900955	109.3778116	0.35295678	5.14E-16
LOC647979	10369.95315	3660.038499	0.35294648	7.83E-77
SDC3	7504.938718	2648.7941	0.35294014	2.30E-73

COL14A1	14715.19038	5183.379601	0.35224686	3.99E-80
NCRNA000	484.1762839	170.0349688	0.35118401	1.29E-22
KLHL24	5062.273305	1775.822398	0.35079544	2.20E-56
PPIL6	193.2002268	67.70861291	0.35045825	2.48E-11
GABRE	362.7243789	127.1126456	0.35043866	7.05E-12
EHF	2919.02817	1022.371488	0.35024379	3.12E-36
ITSN1	2292.202569	802.1786638	0.34995976	3.18E-43
LOC100131	346.568245	121.250589	0.34986064	1.04E-17
MEGF8	4008.937816	1402.262758	0.34978411	8.82E-25
EPB41L4A	507.6426019	177.4828908	0.34962174	1.26E-18
MAPRE3	1871.950643	653.2650161	0.34897556	5.70E-50
ZDHHC8P1	1803.25251	629.1206284	0.34888105	6.70E-12
LOC158376	1283.803886	447.611715	0.34866051	7.33E-42
SREBF1	41832.47624	14575.75631	0.34843159	1.99E-08
ITGA9	448.9376185	156.0830882	0.34767211	1.07E-21
SPTLC3	458.4032343	159.287865	0.34748416	5.08E-22
SEMA3C	7226.957267	2510.920152	0.34743808	1.66E-25
LAMA2	4909.766368	1702.376813	0.34673275	5.24E-32
ABCC1	19232.5369	6628.873843	0.34466976	4.60E-85
LOC100507	307.1081206	105.68453	0.34412809	1.17E-16
COL11A1	39798.19366	13684.07285	0.34383653	3.87E-66
CADM1	36394.67428	12511.35542	0.34376885	1.20E-41
PCDHB7	225.8219717	77.50702018	0.34322178	4.03E-13
PKD1L2	76.69604432	26.1314526	0.34071448	9.75E-06
LOC441869	172.7504225	58.76213072	0.34015622	1.11E-10
AMOT	4852.889435	1650.175267	0.34003974	1.25E-71
KRT15	1146.577535	389.7345554	0.33991121	1.30E-19
FAM114A1	3808.329821	1294.142804	0.33981899	4.23E-33
TTC3	5815.791528	1966.763932	0.33817648	3.35E-75
SLC6A6	21332.05101	7194.722547	0.33727289	5.58E-89
ALS2CR8	521.8255516	175.9560178	0.33719318	1.72E-22
CAPS2	84.03029877	28.32854056	0.33712293	3.52E-06
RNF207	56.69741283	19.04931746	0.33598213	0.0001146
COL9A2	204.3460311	68.59358398	0.33567368	1.93E-08
SLCO4C1	63.54266953	21.24640542	0.33436438	4.74E-05
WBSCR17	8623.842943	2877.527474	0.33367114	1.92E-82
SCRG1	1959.912844	650.9877225	0.33215136	1.63E-54
PRRT2	263.1507348	87.12135073	0.33107014	4.32E-13
NEDD9	18224.98496	6032.011092	0.33097482	6.88E-91
XKR4	335.494652	110.9023181	0.33056359	1.08E-18
LOC729723	34.65338587	11.44799815	0.33035728	0.0023448
FLJ10038	211.6115129	69.90570087	0.33034923	4.26E-13
LOC100129	589.610312	193.2047136	0.32768205	1.24E-28
ZC3H6	414.7560372	135.4785847	0.32664644	2.20E-22
GLT25D2	4565.838403	1489.66259	0.32626266	2.29E-75
LPHN3	306.5984911	99.76111449	0.32538032	7.26E-18
PRICKLE1	1104.235716	357.6159618	0.32385835	5.96E-43

GSX2	69.86454215	22.61988121	0.32376769	1.34E-05
ABLIM1	11958.24966	3862.421016	0.32299217	5.56E-50
LOC728392	520.4857878	167.986545	0.32274953	3.66E-25
SSBP2	1061.184114	339.487028	0.31991341	7.28E-43
ME1	105.0378735	33.54869519	0.31939618	7.98E-08
PLEKHA7	2475.485296	790.1194431	0.31917759	2.11E-64
PLCH1	1988.262262	632.5377948	0.318136	1.37E-57
MATN2	1927.360584	612.9716598	0.31803683	2.05E-58
PCDHGA1	55.62657422	17.5838033	0.31610437	0.0002173
SYNM	9583.927372	3026.894214	0.31583025	3.49E-81
EGR3	52.51377878	16.57611441	0.31565267	0.0001142
CCDC160	99.19812133	31.29024832	0.31543186	4.82E-07
NEBL	5365.748684	1675.88194	0.31232956	6.46E-67
OLFML2B	956.7005726	297.8154628	0.31129433	8.85E-42
C5orf53	322.3791022	100.2189398	0.31087294	1.14E-19
FLG	77.69811025	24.02403646	0.30919718	2.21E-06
RNF150	806.9652952	246.6546514	0.30565707	1.32E-38
CDON	3062.975274	927.1838933	0.30270695	7.72E-64
SGPP2	79.79164659	24.14675427	0.30262258	1.06E-06
CTNND2	5553.287256	1677.930364	0.30215083	6.98E-89
NALCN	178.9760134	53.54434263	0.2991705	1.52E-07
VIT	276.1975119	82.54546462	0.2988639	2.26E-18
WDR78	359.0710063	106.8125702	0.29746921	5.37E-20
KLKB1	33.6169336	9.982483993	0.29694808	0.001354
PACRG	77.70498752	22.98566812	0.29580686	1.20E-06
PLEKHA6	138.0970366	40.66150978	0.29444158	1.15E-10
DOK6	955.3718364	281.180356	0.2943151	7.08E-15
COL4A5	5170.003749	1519.459011	0.29389902	6.33E-91
SSC5D	686.452849	201.4432019	0.29345526	6.89E-28
PTCH1	952.5753953	278.1006635	0.29194609	1.40E-45
C20orf132	57.23455145	16.69646569	0.29172004	2.94E-05
CHI3L2	73.55230118	21.39980269	0.29094675	9.96E-06
PCDH20	5300.529265	1538.942574	0.29033753	3.55E-54
EFHD1	108.636228	31.13685105	0.28661572	5.59E-06
KCNE1	94.4807873	27.07778258	0.28659565	3.98E-08
MST1	29.42298365	8.424931479	0.28633845	0.0024874
C13orf15	57.23455145	16.24100696	0.28376228	1.47E-05
KIAA1908	380.742949	108.0019692	0.28366111	7.32E-25
SYCP2L	33.06947908	9.371261462	0.28338098	0.001194
PARK2	124.4581027	35.22659898	0.28303982	3.80E-10
DFNB59	38.33770626	10.83677561	0.28266625	0.0004422
KIAA1549	1517.752421	427.4650369	0.28164346	1.16E-36
SCN1A	43.58530164	12.27161031	0.28155387	0.0001666
TMOD2	1324.641599	372.4245006	0.28115114	1.35E-57
COL5A1	7736.275075	2173.951205	0.28100749	4.50E-21
BEAN1	252.0702645	70.8213514	0.28095877	3.16E-18
SESN3	2743.034284	768.9674426	0.28033461	1.69E-31

METTL7A	5485.805807	1534.61449	0.27974277	4.86E-99
PCDHGA4	139.0991026	38.86088818	0.27937555	4.17E-11
TTC3P1	749.6482166	208.7731392	0.27849481	5.63E-23
TGFB3	1153.667647	320.2253209	0.27757155	2.97E-54
TMEM63C	220.0097286	60.96158522	0.27708586	1.31E-16
FGF18	159.6658204	44.17308117	0.27665959	6.92E-13
HSD17B3	103.4023872	28.57397619	0.27633768	3.05E-08
HFM1	47.2661834	12.85215339	0.27191012	5.71E-05
DCAF8L2	52.47595381	14.25630864	0.27167317	0.0001379
KRT23	63.03303998	17.09529859	0.27121171	3.35E-06
CLIP3	592.306321	160.5999819	0.27114345	2.50E-36
WNT5A	3848.20602	1040.226674	0.2703147	2.28E-94
KIAA1683	127.6327936	34.49502517	0.27026773	6.34E-11
PCDHGB1	212.1005106	57.17626529	0.26957156	1.32E-16
SCARF1	32.05365861	8.578328746	0.26762401	0.0008746
CPNE4	1146.660774	306.8209373	0.26757777	2.15E-56
CDH8	270.936162	72.4119499	0.26726573	1.34E-20
C5orf4	464.2533023	122.7184697	0.26433516	1.95E-31
PKI55	258.3233645	68.25847653	0.26423656	5.93E-20
GALNTL1	2066.462413	543.7385403	0.2631253	1.18E-77
ARSF	25.1877701	6.624309875	0.26299708	0.0031594
GDA	609.557364	157.6052281	0.25855684	6.22E-39
LOC645323	10039.88906	2574.369139	0.2564141	4.78E-124
PRSS35	15883.83673	4066.979238	0.25604514	3.19E-102
ITGB4	33368.21398	8533.271949	0.25573056	4.65E-12
ELMOD1	727.0773669	184.8718205	0.25426705	2.25E-24
SAMD5	4913.850282	1244.202071	0.25320309	1.13E-35
FGFR3	4799.586665	1212.812684	0.25269107	1.78E-08
VLDLR	7069.019064	1779.704489	0.25176117	2.78E-100
CYP46A1	128.1630549	32.20589885	0.25128848	8.72E-12
PCDHGA2	43.04128576	10.80609616	0.25106351	6.89E-05
CNR1	6412.219588	1604.81042	0.25027378	1.88E-103
PCDHB15	26.77855416	6.685668782	0.24966504	0.0016309
NELL2	4919.451345	1225.669571	0.24914762	7.48E-112
OLFML2A	4120.074763	1020.382057	0.24766105	1.92E-107
ENPP2	1449.485541	356.4006164	0.24588077	2.15E-36
NANOS1	303.4788184	73.87509752	0.24342752	1.20E-24
NAALADL2	315.051725	76.16422384	0.24175149	1.54E-25
SLC38A3	277.8054891	67.15874928	0.24174738	4.44E-23
GLUL	46877.00904	11317.57075	0.24143116	2.34E-151
PI15	1393.256494	336.1878462	0.24129645	3.82E-27
KRT9	327.1445771	78.81913706	0.24093059	2.46E-26
BBOX1	117.0722688	28.17514329	0.24066454	2.75E-11
CCDC67	38.32739036	8.974795104	0.23416139	9.70E-05
SNCAIP	259.8247441	60.50375995	0.23286373	4.09E-13
PCDHGA3	22.58116558	5.250834082	0.23253158	0.0028716
C5AR1	374.4210764	86.54317419	0.23113863	1.48E-30

KCNN3	337.6879087	77.75245581	0.23024945	2.43E-28
FGFBP3	1040.727433	239.2633552	0.22990011	6.95E-63
FEZF1	61.44569456	14.10291137	0.2295183	6.26E-07
MMP15	759.6158728	173.2680586	0.22809958	4.67E-11
LOC375190	256.2160736	56.84115784	0.22184852	1.85E-23
TMC1	75.08462846	16.42271715	0.21872276	1.84E-08
CXCR4	64.5688059	13.76780392	0.21322686	1.06E-07
APC2	182.3088814	38.06558892	0.20879723	3.06E-07
C18orf1	2840.186298	590.9087418	0.20805281	1.60E-115
MIAT	156.4876909	32.35929611	0.20678493	1.41E-16
SLC2A12	11168.88342	2292.735691	0.20527886	6.33E-59
GALNT3	23.11486557	4.700970457	0.20337434	0.0014491
GAS1	157.5378977	31.92978377	0.20268002	9.57E-17
ST6GALNAC	2991.970409	606.2317317	0.20261956	5.64E-121
LDB3	54.09768557	10.95949343	0.2025871	6.23E-07
CACNA2D3	419.9967554	84.19268896	0.20046033	4.77E-38
LOC100130	82.41200565	16.48407605	0.20002032	8.09E-10
ADAMTS7	326.2181611	65.23540986	0.19997479	4.24E-13
UACA	8371.668928	1641.894266	0.19612508	2.04E-59
PTPRQ	80.84873066	15.75250225	0.1948392	5.96E-10
ELMO1	66.17678312	12.85215339	0.1942094	2.05E-08
NCALD	1581.343232	306.7949909	0.19400911	2.68E-75
IRS4	22.0577815	4.181786286	0.18958327	0.0014568
LOC100505	29.953245	5.64730044	0.18853718	0.0001958
C10orf81	2312.352501	434.2403775	0.1877916	3.80E-117
FP588	23.09423377	4.273824647	0.18506025	0.0008755
CES5AP1	56.18434464	10.3482709	0.18418424	1.49E-07
FRZB	3074.286133	563.6728289	0.1833508	1.74E-134
STON1	1712.181664	313.905439	0.18333653	7.65E-104
BVES	57717.42212	10508.54119	0.18206879	1.51E-62
GRIK4	120.2503983	21.70423068	0.18049197	1.39E-14
C9orf44	27.82532233	4.793008818	0.17225349	0.0001511
UG0898H0	433.132937	71.86208628	0.16591231	1.60E-45
LOC440173	74.55092848	12.30228977	0.1650186	2.57E-10
LIPG	4276.618184	694.5991073	0.16241784	5.95E-168
MAP6	123.8900164	19.84225017	0.1601602	3.01E-16
SCUBE3	27730.00168	4399.780682	0.158665	1.14E-233
PAPLN	38.85421308	6.135805157	0.15791866	3.81E-06
FAM198B	6394.286983	971.1422183	0.15187655	1.22E-126
ECM2	933.5685138	139.5069737	0.1494341	1.27E-85
ESRG	1601.338425	239.114691	0.14932177	1.11E-118
TRIM2	17839.15057	2655.182441	0.14884018	7.32E-114
LEFTY2	185.4079223	26.89370586	0.14505155	1.76E-24
NTN4	6178.092243	877.8874289	0.14209685	5.93E-174
NWD1	1203.953783	165.5157085	0.1374768	6.88E-107
ABCB11	329.1555862	44.41615026	0.13493968	6.02E-40
TP53INP1	2937.060494	396.2691934	0.13492034	4.68E-171

LOC729177	17.86383155	2.350485229	0.13157789	0.0011846
RPE65	183.7105406	21.55083342	0.11730864	1.98E-16
GFAP	129064.1243	14897.23493	0.11542506	5.85E-124
NKAIN2	164.9340476	18.43809493	0.11179071	2.70E-25
C10orf107	159.1218045	15.66046389	0.09841809	1.75E-26
LOC154860	498.3042155	43.89696608	0.0880927	2.09E-73
GLDN	2375.103573	199.7959775	0.08412095	2.95E-96
PGM5	459.3090185	37.85319929	0.08241336	1.18E-30
MMRN1	130.2084504	9.493979275	0.0729137	4.58E-25
PCDHB12	14.17951116	0.946329982	0.06673925	0.0008496
LOC284798	16.79643158	0.457825264	0.02725729	4.36E-05
